# Improving Reading Through Videogames and Digital Apps: A Systematic Review

**DOI:** 10.3389/fpsyg.2021.652948

**Published:** 2021-09-16

**Authors:** Mikel Ostiz-Blanco, Javier Bernacer, Irati Garcia-Arbizu, Patricia Diaz-Sanchez, Luz Rello, Marie Lallier, Gonzalo Arrondo

**Affiliations:** ^1^Mind-Brain Group, Institute for Culture and Society (ICS), University of Navarra, Pamplona, Spain; ^2^IE Business School, IE University, Madrid, Spain; ^3^Basque Center on Cognition, Brain and Language, San Sebastian, Spain

**Keywords:** computer-based intervention, dyslexia, first-language, PEDro, PRISMA

## Abstract

**Background:** The use of electronic interventions to improve reading is becoming a common resource. This systematic review aims to describe the main characteristics of randomized controlled trials or quasi-experimental studies that have used these tools to improve first-language reading, in order to highlight the features of the most reliable studies and guide future research.

**Methods:** The whole procedure followed the PRISMA guidelines, and the protocol was registered before starting the process (doi: 10.17605/OSF.IO/CKM4N). Searches in Scopus, PubMed, Web of Science and an institutional reference aggregator (Unika) yielded 6,230 candidate articles. After duplicate removal, screening, and compliance of eligibility criteria, 55 studies were finally included.

**Results:** They were research studies on improving first-language reading, both in children and adults, and including a control group. Thirty-three different electronic tools were employed, most of them in English, and studies were very diverse in sample size, length of intervention, and control tasks. Risk of bias was analyzed with the PEDro scale, and all studies had a medium or low risk. However, risk of bias due to conflicts of interest could not be evaluated in most studies, since they did not include a statement on this issue.

**Conclusion:** Future research on this topic should include randomized intervention and control groups, with sample sizes over 65 per group, interventions longer than 15 h, and a proper disclosure of possible conflicts of interest.

**Systematic Review Registration**: The whole procedure followed the PRISMA guidelines, and the protocol was registered before starting the process in the Open Science Framework (doi: 10.17605/OSF.IO/CKM4N).

## Introduction

Reading is a multifaceted ability involving the decoding of letters and words and language comprehension, which can be further broken into other components and precursors including orthography and alphabetics, phonics, phonemic awareness, vocabulary, comprehension, fluency, and motivation and attention.

Reading acquisition is one of the main keys for school success and a crucial component for empowering individuals to participate meaningfully in society. Yet, for a significant number of children, it is still a challenging skill to be acquired by. Globally, around 250 million children are unable to acquire basic literacy skills (UNESCO). Similarly, many students will not be able to acquire grade-level proficiency to adequately study or learn when they enter high school, which will, in turn, influence their risk of early dropping from the educational system and will possible result in future underemployment and economic success (Polidano and Ryan, 2017). Many different aspects have been related to poor reading outcomes such as prenatal and perinatal risk factors (Liu et al., 2016), gender, socio-economic factors (Linnakyla et al., 2004), or several mental health problems (Francis et al., 2019). Specific Learning Disabilities (SLD) are one of the main challenges. Among SLD, dyslexia is one of the most common, accounting for up to 80% of diagnosed learning disabilities (Shaywitz, 1998).

There is an extensive number of interventions for reading difficulties, given the social relevance and long-term consequences of this problem. Most of them aim to improve skills in five key areas, namely (i) phonemic awareness, (ii) phonics, (iii) fluency, (iv) vocabulary, and (v) comprehension (National Reading Panel, 2000). While traditional assessments rely on paper-based materials, normally used with the supervision of a professional therapist, the number of computer-based intervention tools to improve reading is growing rapidly (see Franceschini et al., 2015; Rello et al., 2017 for some examples). Computer-based interventions have several advantages over more traditional methods. Importantly, they typically require less human resources, and they can provide an attractive environment for children to work with. Additionally, they ease the application of a reading instruction method systematically to all students, reducing the influence of individual differences among teachers. Finally, they are usually programmed to adapt their pace of instruction to the advances of the students, hence facilitating an individualized attention.

Given the novelty and the heterogeneity of electronic interventions, their efficacy has not been systematically evaluated. It has been noted that much of the published research aiming at evaluating these interventions follow unrandomized, small, single-sample, pre and post training protocols (Brooks, 2016). However, in order to be able to evaluate the soundness of these programs, especially in the case of rapidly maturing individuals such as children, it is critical to take into account age-related improvements. Such age-related improvements can only be separated from the effects of interest through the inclusion of experiments with a control group. More generally, a systematic approach to the evidence supporting these interventions must evaluate the risk of many other biases that derive from design decisions as the only way of guiding future research, such us the extent to which all those involved in the experiment were blinded to the treatment condition, or the a-priori statistical power of the studies. Finally, in the case of a rapidly evolving field, it is of paramount importance that the evaluation of the evidence is up to date, and includes the most recent literature.

Hence, we present a systematic review of the electronic interventions aimed to improve first-language reading skills. This systematic review seeks to compare any kind of intervention aimed at improving reading or any of its core components under the same standardized criteria, in order to determine guidelines for assessing the reliability of computer-based interventions, and discriminating which of those are effective. First, we present how we selected the research papers to be included in the review. Second, we attempt to analyze the quality of the selected interventions, proposing key aspects that could be improved in future studies. Finally, we present an overview of the efficacy of those interventions, taking into account the risk of bias of the studies. We believe that this work can benefit professionals who are developing technology-based training and researchers who are evaluating their interventions.

## Materials and Methods

The design and reporting of results of this systematic review was carried out following the guidelines for Preferred Reporting Items for Systematic review and Meta-analysis (PRISMA) (Moher et al., 2009, 2015). A protocol was written and registered before starting data extraction in the Open Science Framework (Ostiz-Blanco and Arrondo, 2018). It was uploaded on May 14, 2019, and it is available in the following URL: https://osf.io/ckm4n/ Searches were carried out in Scopus (Elsevier), PubMed (Medline Plus) and Web of Science (core collection). Additionally, we used an institutional reference aggregator (Unika) based on the EBSCO service to combine references from 61 external databases (psychology profile) (EBSCO Discovery Service; University of Navarra, Búsqueda básica: UNIKA). A full list of databases included the psychology profile of Unika can be found in the Supplementary Material. Initially, searches were limited to the period between 2008 and September of 2017, date in which these searches were carried out. The rationale for the time limit was the fast pace at which computer technologies advance. Hence, any program created over 10 years ago was likely to be outdated. No other limitations were imposed during the search phase. The search was updated on March 2, 2020. Search terms were adapted for each database and limited to abstract, title or keywords.

The general query was *(dyslexia OR reading OR “reading disorder” OR “reading difficulties”) AND (computer-based OR videogame OR “mobile application”)*. The references section of all included articles was used to find further articles of interest.

The PICO (Participants, Intervention, Controls, Outcomes) framework was used to define the key characteristics of our systematic review as follows.

### Participants

Samples considered to be drawn from the general population, that is, without specific disabilities or learning disorders were accepted. Therefore, the fact that a minor percentage of the sample had some of these problems was not a reason for exclusion. Additionally, participants with dyslexia or reading disorders/difficulties were also considered as a valid population. There were no age limitations. Articles were excluded if they were carried out in populations with specific disorders or disabilities other than dyslexia, although if the sample had a proportion of participants with such difficulties the article was not necessarily excluded.

### Interventions

Articles had to deal with any technologically-based intervention aimed at improving reading skills. In this regard, our definition of reading intervention was atheoretical as we relied on the descriptions provided by the authors of the primary papers. However, interventions were broadly classified as supporting reading at the word level (decoding, i.e., phoneme-grapheme mapping), its precursors (phonological awareness -the sound structure of words- or vocabulary learning), or other related skills such as rhythm or attention.

Studies with participants of any age were included, although a majority of articles in children were expected. Whenever an article indicated that their technological intervention was aimed at improving reading or any of its core components, it was accepted. Interventions aimed at learning a second language were excluded.

### Controls

All studies had to include a control group and between group comparisons. Participants of the control group had to fulfill the same criteria than those described in the participants section. Any intervention in the control group was accepted. Hence, we included articles using passive controls such as “Treatment as Usual” (normal classroom) or wait-list, and also articles with an active control (another learning or even reading task).

### Outcomes

At the methodological level, all studies had to be randomized or non-randomized longitudinal interventions (i.e., RCTs or quasi-experimental designs), but any duration or control task was permitted. We included both randomized and non-randomized studies, since the focus of our study was to show how current research is carried out in the field, and not the efficacy of the specific tools. Any outcome measuring an improvement in any of the reading components was accepted, including word reading accuracy, text reading accuracy, reading rate and fluency and phonological skills.

Any type of research format was accepted (articles, thesis, congress proceedings, etc.). Reviews found were used to identify additional references.

Search results were imported to Mendeley. Duplicates were removed automatically using the function provided by this software, and also manually in the cases it was not successful. Two researchers independently reviewed all titles and abstracts. Any article deemed potentially appropriate was downloaded, and the full text was reviewed for further consideration on whether it fulfilled inclusion criteria, or it had to be excluded detailing the reasons for exclusion.

Two researchers extracted data independently and differences were solved by consensus. Data analysis was carried out employing tables and narrative synthesis. The following data was extracted from each article: trained skills (direct reading or other skills); hardware modality; language, country and duration of the intervention; type of control task; sample sizes and age; and results. Risk of bias was evaluated using the Physiotherapy Evidence Database tool (PEDro) (Blobaum, 2006; Physiotherapy Evidence Database, 2012). This scale evaluates 11 items: inclusion criteria and source, random allocation, concealed allocation, similarity at baseline, subject blinding, therapist blinding, assessor blinding, completeness of follow up, intention-to-treat analysis (the analysis of the results of a study according to the initial intervention assignment instead of according to the group at the end of the intervention time), between-group statistical comparisons, and point measures and variability (whether the study includes adequate measure of the size of the treatment effect and its variation, e.g., mean effect in each of the groups and its confidence interval). Each item is rated as “yes” or “no,” and the total PEDro score is the number of items met. Afterwards, studies were divided into three groups: high (less than four points), medium (between four and seven) or low risk of bias (between 8 and 11). Finally, studies were divided into four groups according to their combined sample size and the smallest effect size that they would be able to detect (assuming a two-sample *t*-test between two equally sized groups and 0.8 power): very large effects (Cohen's d over 1, combined sample size under 53), large effects (Cohen's d over 0.8, combined sample between 53 and 128), medium effects (Cohen's d over 0.5, combined sample size between 128 and 786), and small effects (Cohen's d over 0.2, combined sample size over 786) (Cohen, 1988). This categorization was driven by the fact that low power combined with a high proportion of statistically significant results could indicate a high proportion of false positives in the literature (Szucs and Ioannidis, 2017). Conflicts of interest declared in the included articles were also extracted (Cristea and Ioannidis, 2018).

## Results

**Figure 1:**
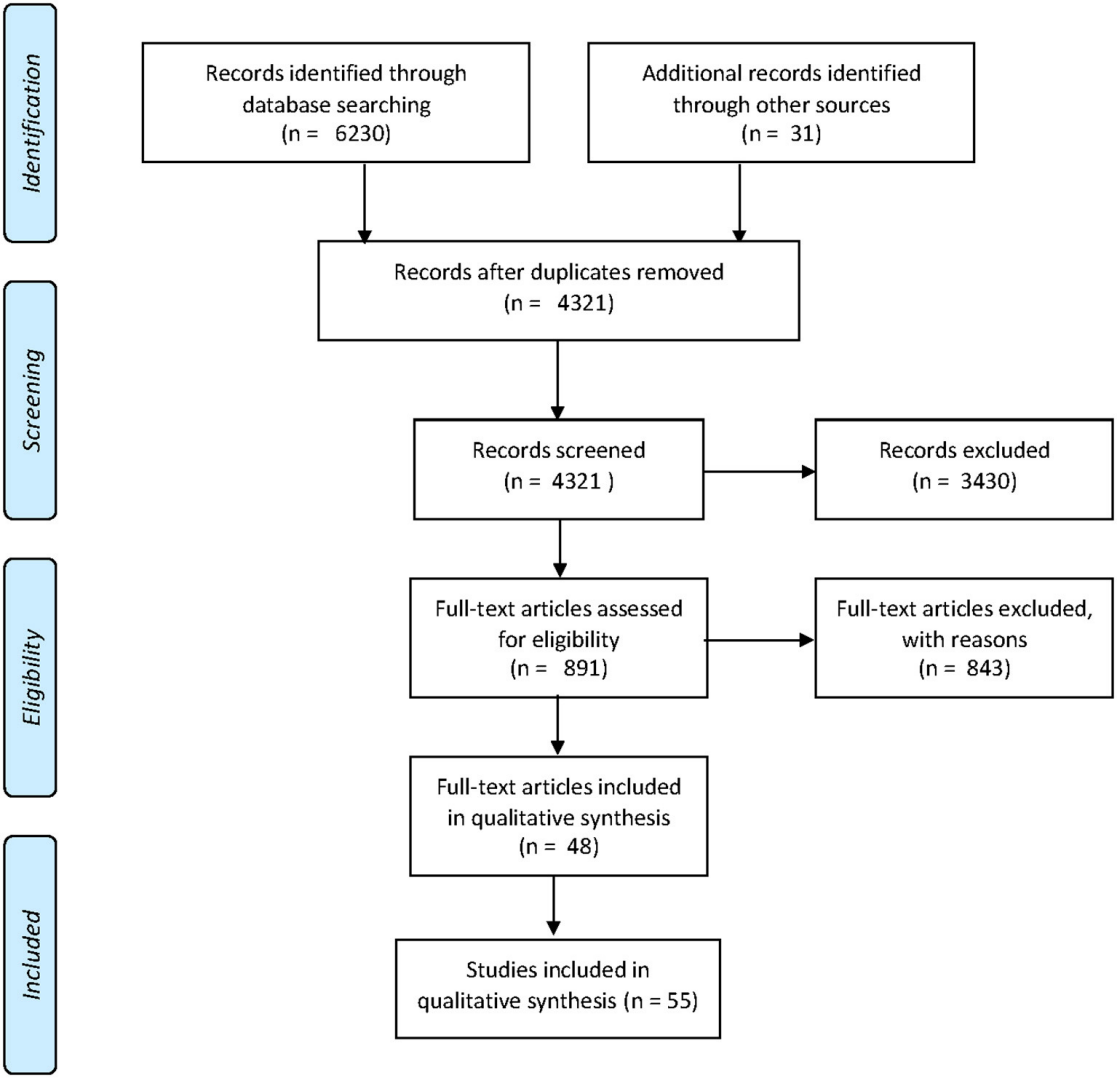
Review flowchart: a flowchart of the screening and inclusion of articles in our systematic review.

The search in four databases and a reference aggregator yielded 6,230 results. After elimination of duplicates, screening and full text evaluation, 48 articles fulfilled inclusion criteria (Borman et al., 2008; Given et al., 2008; Jiménez and Rojas, 2008; Macaruso and Walker, 2008; Watson and Hempenstall, 2008; Deault et al., 2009; Ecalle et al., 2009; Macaruso and Rodman, 2009, 2011; Pindiprolu and Forbush, 2009; Shelley-Tremblay and Eyer, 2009; Wild, 2009; Arvans, 2010; Huffstetter et al., 2010; Jimenez and Muneton, 2010; Rasinski et al., 2011; Rogowsky, 2011; Saine et al., 2011; Soboleski, 2011; Tijms, 2011; Wolgemuth et al., 2011, 2013; Di Stasio et al., 2012; Falke, 2012; Ponce et al., 2012, 2013; Williams, 2012; Franceschini et al., 2013; Heikkilä et al., 2013; Hill-Stephens, 2013; Mcmurray, 2013; Reed, 2013; Savage et al., 2013; Kamykowska et al., 2014; Plony, 2014; Rello et al., 2015; Shannon et al., 2015; Beaudry, 2016; De Primo, 2016; Jackson, 2016; O'Callaghan et al., 2016; Deshpande et al., 2017; Messer and Nash, 2017; Moser et al., 2017; Nee Chee et al., 2017; Rosas et al., 2017; Daleen et al., 2018; Flis, 2018). Since some of the articles included more than one study, 55 studies were finally analyzed. A flowchart of the screening and inclusion of articles in our review is depicted in Figure 1. Excluded articles and the exclusion criteria they fulfilled are detailed in Supplementary Table 1; a Venn diagram summarizing the reasons for exclusion and the number of articles excluded for each of them is shown in Supplementary Figure 1.

The key characteristics of all included studies are summarized in Table 1. Study methods and results were very heterogeneous, and 33 different training programs were included. The most employed tools were *Fast ForWord* (in seven studies), Abracadabra (in six) and Graphogame (in four). Most tools were present in only one study, and seven did not provide the name of the software being evaluated. Most tools were in English (69% of the studies), followed by Spanish (15%) and six other languages with only one study each. In fact, nearly half of studies had been carried out in the United States (49%). The vast majority (91%) used computers as hardware, instead of laptops/tablets (7%) or videogame systems (2%).

**Table 1:** Articles included in the systematic review, and main characteristics of the computerized training used in each of them.

**ID**	**Training name**	**Training skill**	**Tech**	**Lang**.	**Country**	**Popul**.	**Training duration (h)**	**Control task**	**Sample size**	**Age**	**Sig. results**	**Detectable ES**	**CoI**
Arvans (2010)	Read Natu-rally	R	PC	English	USA	D	25	SE	82	7–10	No	Large	NR
Beaudry (2016)	The First 4,000 Words	R,M	PC	English	USA	G	10	NTLT	37	7–8	No	Very large	NR
Borman et al. (2008)	Fast For Words	R,L,M,A	PC	English	USA	D	50	NTLT	141	7–8	No	Medium	NR
	Fast For Words	R,L,M,A	PC	English	USA	D	50	NLT	274	12–13	Yes	Medium	NR
Daleen et al. (2018)	No name	V	Tablet	isiXhosa	South Africa	G	2	SE	65	6–8	Yes	Large	No
De Primo (2016)	Fast For Words	R,L,M,A	PC	English	USA	D	50	SE	318	11–15	Yes	Medium	Yes*
Deault et al. (2009)	Abracadabra	R,L,Ph	PC	English	Canada	G	13	SE	144	6–7	Yes	Medium	NR
Deshpande et al. (2017)	Tara Akshar	R,Ph	PC	Hindi	India	A	43,	SE	717	33,55	Yes	Medium	NR
Di Stasio et al. (2012)	Abracadabra	R,L,Ph	PC	English	Canada	G	10	ATT	49	6–7	Yes	Very large	NR
Ecalle et al. (2009)	No name	Ph	PC	French	France	D	10	ATT	26	No info	No	Very large	NR
Falke (2012)	Success-maker	R	PC	English	USA	G	75	SE	108	10–11	Yes	Large	NR
Flis (2018)	Abracadabra	R,L,Ph	PC	English	Canada	D	13	SE	82	6–7	No	Large	NR
Franceschini et al. (2013)	Action video games	A	Wii	English	Italy	D	12	ATT	20	7–13	Yes	Very large	NR
Given et al. (2008)	Fast For Words	R,L,M,A	PC	English	USA	G	44	SE	28	11–12	No	Very large	NR
	Success-maker	R,V,Ph	PC	English	USA	G	44	SE	24	11–12	No	Very large	NR
Heikkilä et al. (2013)	No name	Ph	PC	Finish	Finland	D	1	NLT	152	7–9	Yes	Medium	NR
Hill-Stephens (2013)	Ear-obics	R,V,Ph	PC	English	USA	D	75	SE	8,055	7–8	No	Small	No
Huffstetter et al. (2010)	Head-sprout Early reading	R	PC	English	USA	D	20	NLT	62	4–5	Yes	Large	NR
Jackson (2016)	Nova–Net	R	PC	English	USA	D	No info	SE	149	7–8	Yes	Medium	NR
Jimenez and Muneton (2010)	No name	R	PC	Spanish	Spain	D	No info	SE	43	8–10	Yes	Large	NR
	No name	R	PC	Spanish	Spain	D	No info	SE	42	8–10	Yes	Large	NR
	No name	R	PC	Spanish	Spain	D	No info	SE	42	8–10	Yes	Large	NR
Jiménez and Rojas (2008)	Tradislexia	Ph	PC	Spanish	Spain	D	9	SE	62	9–12	Yes	Very large	NR
Kamykowska et al. (2014)	Grapho-game	Ph	PC	Polish	Poland	G	6	NLT	24	6–7	No	Very large	NR
Macaruso and Walker (2008)	Early Reading	R,V,Ph	PC	English	USA	G	24	NLT	94	4–5	Yes	Large	NR
Macaruso and Rodman (2009)	Lexia Reading	R,Ph	PC	English	USA	G	21	NLT	47	11–13	Yes	Very large	NR
Macaruso and Rodman (2011)	Early Reading	Ph	PC	English	USA	G	3	NLT	38	4–5	No	Very large	NR
Mcmurray (2013)	Lexia Reading	Ph	PC	English	Ireland	D	20	NTLT	106	6–7	No	Large	NR
Messer and Nash (2017)	Trainer-text	M,Ph	PC	English	UK	D	31	SE	78	7–8	No	Large	NR
Moser et al. (2017)	No name	Ph	Tablet	English	USA	G	12	SE	29	9–10	Yes	Very large	NR
Nee Chee et al. (2017)	Chinese-Skills	Ph	Smartphone	Chinese	Malaysia	G	No info	SE	48	6–7	Yes	Very large	NR
O'Callaghan et al. (2016)	Lexia Reading	Ph	PC	English	Ireland	D	17	SE	98	4–6	Yes	Large	NR
Pindiprolu and Forbush (2009)	Funnix	Ph	PC	English	USA	D	223	ATT	25	6–8	No	Very large	NR
Plony (2014)	Read 180	R,V	PC	English	USA	D	225	NTLT	228	11–14	No	Medium	NR
Ponce et al. (2012)	e-PELS	R	PC	Spanish	Chile	D	45	SE	1,041	9–10	Yes	Small	NR
Ponce et al. (2013)	No name	R	PC	Spanish	Chile	D	21	SE	1,562	6–14	Yes	Small	NR
Rasinski et al. (2011)	Reading Plus	R	PC	English	USA	D	36	SE	1,6143	9–16	Yes	Small	NR
Reed (2013)	Fast For Words	R,L,M,A	PC	English	USA	D	35	SE	51	6–9	No	Very large	NR
	Sonday	R,Ph	PC	English	USA	D	35	SE	42	6–9	No	Very large	NR
Rello et al. (2015)	Dys-Eggxia	Ph	Tablet	Spanish	Spain	D	4	ATT	48	6–11	No	Very large	NR
Rogowsky (2011)	Fast For Words	R,L,M,A	PC	English	USA	G	31	SE	81	11–12	Yes	Large	NR
Rosas et al. (2017)	Grapho-game	Ph	PC	Spanish	Chile	D	13	SE	87	6–7	No	Large	No
Saine et al. (2011)	Grapho-game	Ph	PC	Finish	Finland	D	66	NTLT	50	7	Yes	Very large	NR
	Grapho-game	Ph	PC	Finish	Finland	D	66	SE	141	7	Yes	Medium	NR
Savage et al. (2013)	Abracadabra	R,L,Ph	PC	English	Canada	G	22	SE	1,067	5–8	Yes	Small	NR
Shannon et al. (2015)	Accele-rated Reader	R	PC	English	USA	G	No info	SE	346	6–10	Yes	Medium	NR
Shelley-Tremblay and Eyer (2009)	Reading Plus	R	PC	English	USA	G	40	SE	49	7–8	Yes	Very large	NR
Soboleski (2011)	Fast For Words	R,L,M,A	PC	English	USA	G	75	SE	360	7–8	No	Medium	NR
Tijms (2011)	LEXY	R	PC	Dutch	The Netherlands	D	66	NTLT	96	9–12	Yes	Large	NR
Watson and Hempenstall (2008)	Funnix	Ph	PC	English	USA	G	No info	SE	16	4–5	Yes	Very large	NR
	Funnix	Ph	PC	English	USA	G	No info	SE	15	6–7	No	Very large	NR
Wild (2009)	Rhyme and Analogy	Ph	PC	English	UK	G	7	NLT	84	5–6	Yes	Large	NR
Williams (2012)	CompassLearning Odyssey	R	PC	English	USA	G	90	SE	188	11–13	Yes	Medium	NR
Wolgemuth et al. (2011)	Abracadabra	R,L,Ph	PC	English	Australia	G	20	SE	166	5–9	Yes	Medium	NR
Wolgemuth et al. (2013)	Abracadabra	R,L,Ph	PC	English	Australia	G	30	SE	308	5–9	Yes	Medium	NR

Training Skill: A, attention; L, listening; M, working memory; Ph, phonology; R, reading; V, vocabulary. Population: A, illiterate adults; D, reading disorders or difficulties; G, general population (children). Control task: ATT, another technological training; NLT, non-literacy tasks; NTLT, non-technological literacy task; SE, standard education. Detectable ES: smallest effect-sizes the study could potentially detect assuming a two-sample t-test. CoI, Conflict of interest; NR, Not reported. *Adequately managed.

Regarding the reading-related skill trained, 60% of the interventions directly aimed to improve reading, 58% worked on phonology and the remaining studies addressed indirect skills such as oral comprehension (24%), working memory (15%), attention (15%) or vocabulary (13%). Studies were mainly carried out in two different kinds of population: children/adolescents either with (54%) or without (44%) reading difficulties. Only one study (Deshpande et al., 2017) included illiterate adults (2%). Consequently, the median age of the participants was 8.6 years old. Duration of the interventions was highly variable, ranging between 1.25 and 225 h.

The most common control task against which the interventions were compared was standard education (65% of the cases), whereas the remaining studies used active tasks: 15% used a non-linguistic task such as mathematics or art, 11% a non-technological reading intervention, and 9% a different technological reading training. Sample sizes were also heterogeneous, ranging between 15 and 16,243, with a median of 82. Only 9% of the studies had a sample size big enough to be able to consistently detect small effects and 18% of the studies could identify medium-sized effect-sizes. Conversely, 24% would only have been able to detect large effects, and 38% of the studies were only capable of consistently showing statistically significant very large effects.

**Figure 2:**
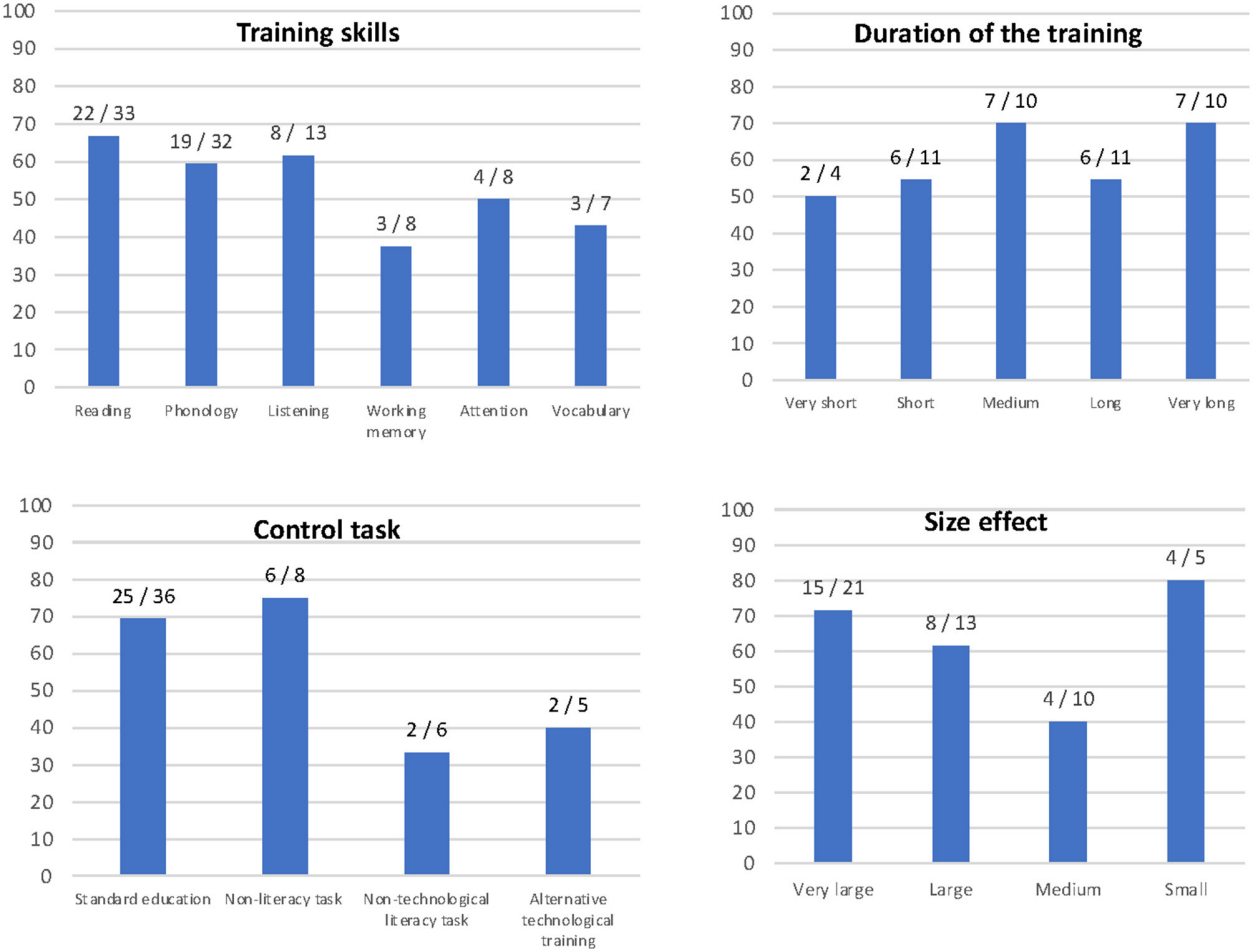
Statistically significant studies: proportion of studies with statistically significant results in relation to four study characteristics: training skill, duration of the training, control task used and effect size that studies would have been able to consistently detect.

Thirty-four studies (64%) reported statistically significant effects. Figure 2 shows the proportion of studies with significant results in relation to the different study characteristics, namely training skill, duration of the training, control task used and effect size that studies would have been able to consistently detect.

**Figure 3:**
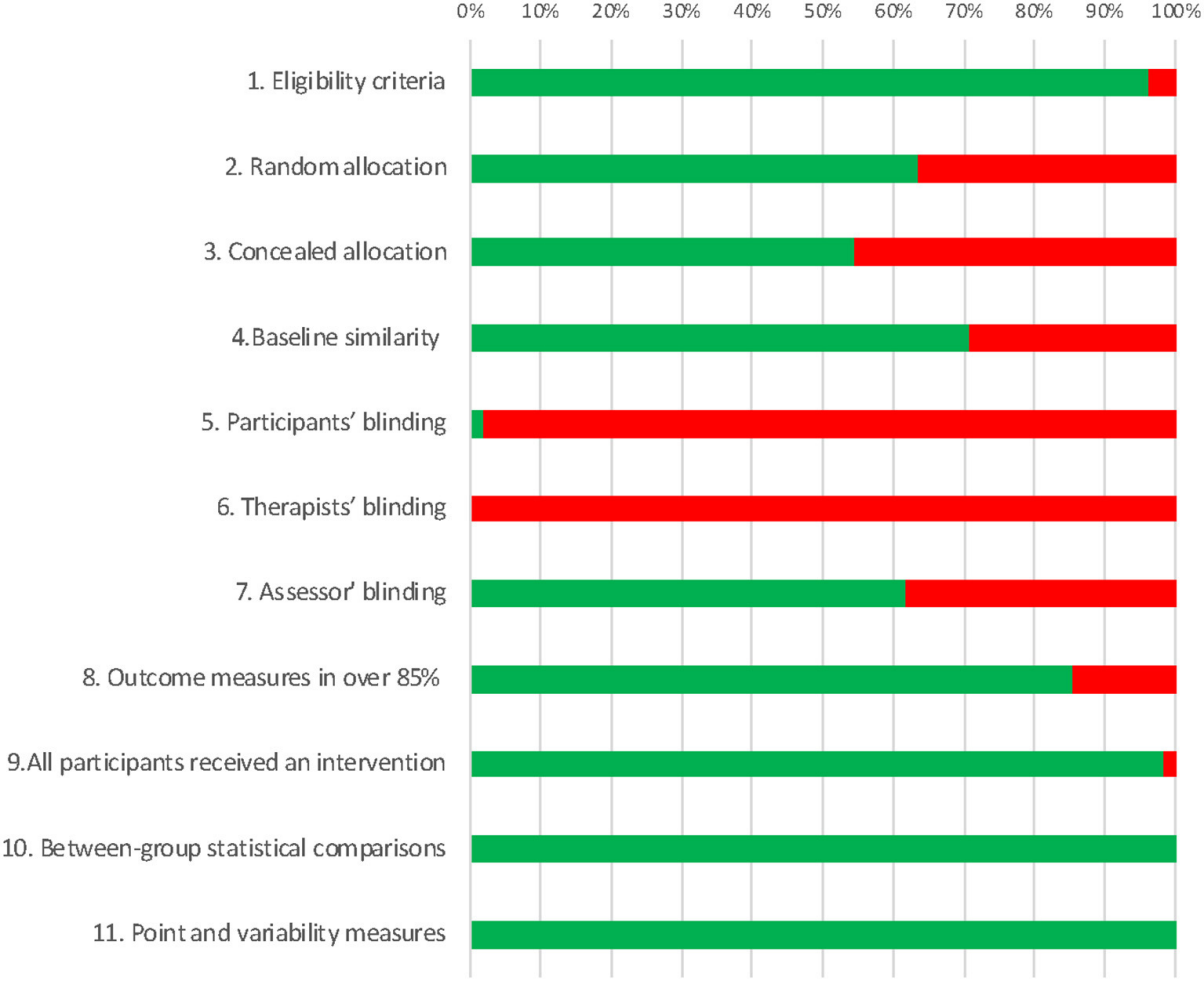
Risk of bias assessment: percentage of included articles fulfilling each of the PEDro scale items.

Using the PEDro tool, we assessed the risk of bias for each of the studies included in the review (Figure 3; Supplementary Table 2.2). Twenty-six and 29 studies had a medium and low risk of bias, respectively. Regarding conflicts of interest, only one study reported competing interests that could suppose a risk of bias for their conclusions, but such conflicts were adequately managed. Three studies declared no conflicts of interest. Importantly, 51 studies did not include a conflict of interest statement, and therefore the risk of bias due to this issue cannot be estimated.

Next, we report in detail the characteristics of those studies with the highest quality, since their results should be the more reliable for the research questions on this topic. Five studies were considered to have the highest quality in terms of a low risk of bias, at least a medium treatment length (over 15 h), and a sample size allowing the detection of medium-sized effects. Two of them were included within the same article by Borman et al. and used a similar methodology: they evaluated the effectiveness of *Fast ForWord* (a computerized reading intervention that uses the principles of neuroplasticity to improve reading and learning) in samples of individuals with low reading skills against an active control condition of arts and gymnastics activities, and utilized the *Comprehensive Test of Basic Skills, Fifth Edition* (CTBS/5). The first study tested 248 children between seven and 8 years and the second 453 between 12 and 13 years. Only the second study reported statistically significant differences. Another study evaluated *Graphogame* (a computer game designed to provide intensive training in rapid recognition of grapheme-phoneme associations and further reading skills) in Finnish (Saine et al., 2011), in 50 seven-year-old children at risk of developing reading problems randomized to either a regular reading intervention or a computer assisted intervention. The training took 66 h and performance was compared to usual classroom activities. Significant training-induced improvements were found on letter naming, reading fluency and spelling. Additionally, these groups were compared to the mainstream reading group. The other two studies used Abracadabra (a free access, web-based literacy tool that contains texts and strategies to support word reading, phonics, reading and listening comprehension, and reading fluency) on Australian samples of children between 5 and 9 years old recruited from the average population of Canada and Australia, respectively, utilizing the normal classroom curriculum as the control task (Savage et al., 2013; Wolgemuth et al., 2013). One of the studies found statistically significant results in phonological awareness and reading after 30 h with a sample size of 308 participants, whereas the other study reported differences in phonological skill and letter knowledge after 22 h of training, and a sample size of 1,067 participants. It is important to highlight that none of these articles have a conflict of interest statement, so the possibility of undeclared competing interests cannot be completely discarded. Also, the variety of designs (randomized at the individual level or the classroom level, comparing computerized trainings against other remediation measures or the normal classroom dynamic, among others), statistical analyses (such as ANCOVA, ANOVA, hierarchical linear models and linear regression) and completeness of reporting precluded the calculation of any meaningful common effect size from these studies.

## Discussion

Technologies evolve at a very fast pace, and educational digital interventions are not an exception neither at the school level (Hubber et al., 2016), nor at the level of University (Arrondo et al., 2017) or non-formal education (Ostiz-Blanco et al., 2016). The current systematic review provides an overview of the characteristics of published research using digital tools and interventions aimed at improving reading processes. The overarching objective of our analysis is to provide a description of the research available on this topic, in order to guide future investigations on this topic. Organizations such as What Works Clearinghouse provide guidance on which specific interventions have a greater evidence-based support (U.S. Department of Education, Institute of Education Sciences, 2009, 2010, 2013a,b, 2017), and therefore we did not intend to evaluate the efficacy of existing tools. Conversely, we present an overview on how research is carried out on this field of study, showing the strengths and weaknesses to potentiate the former and mitigate the latter.

From a methodological point of view, the protocol of this systematic review was pre-registered to reduce the risk of bias (Ostiz-Blanco and Arrondo, 2018), international guidelines were followed throughout its development and reporting (Moher et al., 2009, 2015), and searches were carried out over three different databases and an aggregator that combines results from over sixty additional databases. After screening over four thousand initial articles, our final review comprised 55 studies that included a control group and inter-group comparisons. Indeed, among the most frequent reasons for exclusion was the fact that many studies did not include such a group or only evaluated intraindividual changes between pre and post-intervention phases. However, without proper control groups and comparisons, studies can hardly assess efficacy, especially when dealing with populations developing very fast such as children.

As stated above, our systematic review was not designed to evaluate the efficacy of interventions. Moreover, the very different characteristics of the studies and tools reviewed hamper the possibility of adequately comparing research outcomes, even if effect sizes had been calculated. In any case, our review highlights a number of features that, from a methodological point of view, are shared by the highest quality studies included in our analysis. Five studies fulfilled criteria to be considered as with a high quality (Borman et al., 2008; Saine et al., 2011; Savage et al., 2013; Wolgemuth et al., 2013): a low risk of bias, a treatment duration over 15 h and a combined sample size over 128, and hence capable of detecting at least medium-sized effects. These studies showcase the experimental design that future studies should try to emulate; additionally, their results are the most informative regarding effectiveness evaluation.

Most intervention programs were implemented on computers, whereas we found few studies using *smartphones, tablets* or videogame systems. This might seem surprising since mobile technologies offer important advantages over desktop computers regarding usability and motivation, including the fact that they are touch- and movement-responsive or that children associate them to leisure activities. As there are big delays between the creation of a program, its testing and the publication of results, it is likely that this proportion would change over time, and upcoming studies will reflect an integration of this hardware within educational interventions. Similarly, the majority of the published interventions were carried out in English, some in Spanish, and very few in other languages. It is unknown whether the underrepresentation of other languages derives from a lack of tools for language training in those languages or a lack of publication of research results in international journals. In this regard, their potential world-wide audience could make digital systems especially suited for the implementation of programs that train language-independent reading-related skills, since such programs could be distributed with only minor changes (Burgstahler, 2015). Regarding the type of language skill trained, most studies provided either a direct reading training or phonological training. Nevertheless, the number of studies centered on the improvement of other skills, such as hearing or visual attention, was still relevant. Studies that directly trained reading skills had a higher proportion of statistically significant results in our review, and similar findings have been reported in the literature. This could indicate that direct language training has higher efficacy than other approaches and should be recommended as the default approach. However, indirect training could also have advantages in some cases. For example, it could be useful as an early intervention for very young children at risk of later developing reading problems (Lyytinen et al., 2009; Snowling, 2013). Furthermore, it could increase motivation, as the training does not focus on an area where the individual may feel impaired (Wouters et al., 2013). Remarkably, all high-quality studies included in our review used these direct-training approaches, which indicates both a higher level of evidence for such interventions and the need for further high-quality research on the effectiveness of non-direct trainings. Similarly, studies were typically aimed at primary school students that were either acquiring or consolidating their language skills. Research on other age segments, including preschoolers, secondary school students, or adults is lacking. Duration of interventions was highly variable. Whereas, it is not clear from our results if longer interventions lead to better outcomes, this seems a reasonable assumption. Without any doubt, very short interventions were related to a lower rate of positive results. The creation of engaging games that children can use independently for extended periods could be an effective strategy to obtain reading improvements without individuals feeling an increase in their educational workload over time.

Among the most useful aspects to take into account when developing future studies is the risk of bias of previous published research. Moreover, it has been recently proposed that reviews should only be considered systematic if they evaluate the risk of bias of included studies (Krnic Martinic et al., 2019), a step that is rarely carried out in systematic reviews in psychology (Leclercq et al., 2019). Risk of bias was assessed by using the PEDro scale (Blobaum, 2006; Physiotherapy Evidence Database, 2012). Results indicated that none of the studies here had a high risk of bias. This is partly explained by some of our a-priori criteria of inclusion which to some extent were more stringent than the options provided by the scale. For example, by requiring that studies had to include inter-group comparisons, the item 10 and the item 11 of the scale were satisfied in all cases. However, other items of the scale, such as whether assessment agents were blinded to the treatment group of the participants require complex organization when carrying school-based research and were very rarely fulfilled. Future studies should be designed to try to overcome previous limitations and should improve randomization and blinding to all those involved in the research project (participants, therapists and assessors). While we required that all studies included a control group, not all research involved the same type of controls; and this could greatly influence the interpretation of results. Two thirds of the interventions were carried out in addition or instead of the standard classroom education and compared to the latter, whereas only a few studies used active linguistic tasks as controls. Relatedly, the few studies using active linguistic tasks had a reduced proportion of significant results. However, studies without such controls would at most be able to conclude that the methodology tested works, but would not be able to evaluate if the training provides an improvement over any existing methodology. In this regard, since the development and implementation of newer methodologies have important associated costs, it would be hard to justify the expenses without evidence of their differential effectiveness. In addition to risk of bias, we evaluated sample sizes and their power to detect given effect sizes. The median sample size was 82 participants, whereas only one third of the studies could detect medium effect sizes and <10% small ones. While in theory larger sample sizes do not lead to a lower bias, it has been shown that small studies are more prone to publication bias (e.g., only to be published if positive) and have more unstable results (e.g., are more dependent of analytic choices made by the researcher) (Kühberger et al., 2014; Rubin, 2017). Hence, sample sizes seem a key factor for improvement in future research.

The most researched interventions were the commercial programs *Fast ForWords* and *Abracadabra*. Four out of the five studies fulfilling our excellence criteria comprised these tools. As it occurs in the case of biomedical research, partnerships between universities and publishers seems a promising way to carry out well-powered and designed studies and manage their costs. However, such kind of research has its own conflicts of interest that should also be taken into account in future research. Relatedly, another venue for improvement is an increase of mandatory declarations of competing interests on articles. The great majority of the articles in our systematic review did not include one, even in the cases when they were dealing with commercial applications.

To conclude, we found an increasing number of studies that use computer games or apps for the improvement of reading skills and they seem a promising alternative in education. However, research is still in its infancy and studies up to date have important limitations that hinder their usefulness to guide decisions in the educational domain. Future studies should be better-designed randomized controlled trials, with larger sample sizes, and that are able to answer the question on whether a computerized intervention adds any value to existing methods. Partnerships between universities and publishers or other entrepreneurial initiatives could be a potential way of moving forward, but conflicts of interest in such cases should be outlined.

## Data Availability Statement

The raw data supporting the conclusions of this article will be made available by the authors, without undue reservation.

## Author Contributions

MO-B and GA: conceptualization. MO-B, GA, and JB: funding acquisition and formal analysis. GA: methodology and supervision. MO-B, GA, JB, IG-A, and PD-S: data extraction. MO-B, GA, JB, and LR: writing–original draft. MO-B, GA, JB, IG-A, PD-S, LR, and ML: writing–review and editing. All authors contributed to the article and approved the submitted version.

## Funding

This research was supported by the Institute for Culture and Society (ICS, University of Navarra), Obra Social La Caixa, Fundación Caja Navarra, Fundación Banco Sabadell, and the Severo Ochoa program grant SEV-2015-049.

## Conflict of Interest

MO-B, ML, and JB have developed a videogame to improve reading in people with dyslexia (Jellys). LR's research is also focused on computer-based tools to improve reading in people with dyslexia (DytectiveU). None of these computer-based tools are expected to yield any present or future direct economic profits. The remaining authors declare that the research was conducted in the absence of any commercial or financial relationships that could be construed as a potential conflict of interest.

## Publisher's Note

All claims expressed in this article are solely those of the authors and do not necessarily represent those of their affiliated organizations, or those of the publisher, the editors and the reviewers. Any product that may be evaluated in this article, or claim that may be made by its manufacturer, is not guaranteed or endorsed by the publisher.
