# Supplementary material for: Improving Reading Through Videogames and Digital Apps: A Systematic Review
Source: Front Psychol. 2021 Sep 16;12:652948. doi: 10.3389/fpsyg.2021.652948 (PMC8481589; doi:10.3389/fpsyg.2021.652948)
Supplement: Supplementary file 1 [file Data_Sheet_1.DOCX]

Improving reading through videogames and digital apps: a systematic review

Mikel Ostiz-Blanco^1^, Javier Bernacer^1^, Irati Garcia-Arbizu^1^, Patricia Diaz-Sanchez^1^, Luz Rello^2^, Marie Lallier^3^, Gonzalo Arrondo^1*^

^1^ Mind-Brain Group, Institute for Culture and Society (ICS), University of Navarra, Pamplona, Spain

^2^ IE Business School, IE University, Madrid, Spain

^3^ BCBL (Basque Center on Cognition, Brain and Language), San Sebastian, Spain

Supplementary Material

**Index of contents**

[Supplementary methods 1: List of databases included in the psychology profile of Unika 2](#_Toc46738311)

[Supplementary figure 1: Venn diagram summarizing the reasons for exclusion of articles 3](#_Toc46738312)

[Supplementary table 1. Excluded articles with reasons 4](#_Toc46738313)

[Supplementary table 2.1 Items of the PEDro scale 71](#_Toc46738314)

[Supplementary table 2.2. Risk of bias of included studies 71](#_Toc46738315)

[Supplementary table 3. PRISMA 2009 Checklist 75](#_Toc46738316)

# Supplementary methods 1: List of databases included in the psychology profile of Unika

1. Academic Search Index
2. ACLS Humanities E-Book
3. APA PsycArticles
4. APA PsycBooks
5. APA PsycInfo
6. Aphasiology Archive
7. Archive of European Integration
8. arXiv
9. Atla Religion Database with AtlaSerials
10. BDSL (Bibliographie der Deutschen Sprach-und Literaturwissenschaft)
11. BioOne Complete
12. Books at JSTOR
13. British Library EThOS
14. Cairn.info
15. Catálogo de la Biblioteca de la Universidad de Navarra
16. Center for Research Libraries
17. Communication & Mass Media Complete
18. Complementary Index
19. Credo Reference
20. DADUN
21. Dialnet Plus
22. Digital Access to Scholarship at Harvard (DASH)
23. DigiZeitschriften
24. Directory of Open Access Journals
25. eArticle
26. eBook Academic Collection (EBSCOhost)
27. eBook Collection (EBSCOhost)
28. ECONIS
29. EconLit
30. Emerald Insight
31. ERIC
32. Fuente Académica Premier
33. Gale eBooks
34. Harvard Library Bibliographic Dataset
35. HathiTrust
36. HBO Kennisbank
37. HeinOnline
38. JoVE Science Education Database
39. JSTOR Journals
40. MEDLINE
41. Minority Health Archive
42. OAIster
43. OAPEN Library
44. OpenAIRE
45. ORBi
46. Oxford Handbooks Online
47. Oxford Medicine Online
48. Oxford Reference
49. Oxford Scholarship Online
50. Persée
51. ProjectMUSE
52. Psychology and Behavioral Sciences Collection
53. Research Starters
54. SAGE Knowledge
55. SciELO
56. ScienceDirect
57. Shamaa
58. Social Sciences Citation Index
59. SSOAR – Social Science Open Access Repository
60. Supplemental Index
61. University Press Scholarship Online

# Supplementary figure 1: Venn diagram summarizing the reasons for exclusion of articles

Numbers refer to the reasons for exclusion and are detailed below

# Supplementary table 1. Excluded articles with reasons

| **Reference** | **Reason for exclusion** |
| --- | --- |
| (ED), N. C. for E. S. (2013). PISA 2012 Data Tables, Figures, and Exhibits. NCES 2014-024. National Center for Education Statistics. National Center for Education Statistics. Retrieved from http://search.ebscohost.com/login.aspx?direct=true%7B&%7Ddb=eric%7B&%7DAN=ED544505%7B&%7Dsite=ehost-live | 10. Educational-related but it is not about reading |
| 2018 10th International Conference on Virtual Worlds and Games for Serious Applications, VS-Games 2018 - Proceedings. (2018). 2018 10th International Conference on Virtual Worlds and Games for Serious Applications, VS-Games 2018 - Proceedings. https://www.scopus.com/inward/record.uri?eid=2-s2.0-85057331929&partnerID=40&md5=80b75372a3b62739a1b8a9c28c8d894e | 11. Type of study excluded (review, non-primary references…) |
| Abdellah, A. S. (2009). Eye vs. text movement; which technique leads to faster reading comprehension? Source: CDELT Occasional Papers (Vol. 48). Online Submission. Retrieved from http://ezproxy.si.unav.es:2048/login?url=http://search.ebscohost.com/login.aspx?direct=true&AuthType=ip,url&db=eric&AN=ED536735&lang=es&site=eds-live&scope=site | 9. Training oriented to a second-language learning |
| Abdurrodjak, M., Mud’Is, M. H., Qodim, H., Khaerani, I. F. S. R., Rosidin, U., & Busro, B. (2019). Sound matching on the translation of Al-Quran ayat as a learning media for children using mobile-based fast fourier transform and divide conquer algorithm. In W. I. D. A. A. A. C. U. Abdullah A.G. Nandiyanto A.B.D. (Ed.), Journal of Physics: Conference Series (Vol. 1402, Issue 7). Institute of Physics Publishing. https://doi.org/10.1088/1742-6596/1402/7/077060 | 10. Educational-related but it is not about reading |
| Ablyaev, M., Abliakimova, A., & Seidametova, Z. (2019). Design of mobile augmented reality system for early literacy. In Y. V. Y. V. M. H. C. S. A. Ermolayev V. Mallet F. (Ed.), CEUR Workshop Proceedings (Vol. 2387, pp. 274–285). CEUR-WS. https://www.scopus.com/inward/record.uri?eid=2-s2.0-85068799696&partnerID=40&md5=6ec1be236fd5c746a6825f1b45d1c10a | 2. It is a reading-related technology but it is not a training (test, support…) |
| Ablyaev, M., Abliakimova, A., & Seidametova, Z. (2020). Developing a Mobile Augmented Reality Application for Enhancing Early Literacy Skills. Communications in Computer and Information Science, 1175 CCIS, 163–185. https://doi.org/10.1007/978-3-030-39459-2_8 | 2. It is a reading-related technology but it is not a training (test, support…) |
| ADHI, A. M. K., NURKAMTO, J., & DRAJATI, N. A. (2019). Assessing the Reading Competence Needs of Senior High School Students in National Examination: Teachers’ Perspective. International Journal of Educational Research Review (IJERE) VO  - 4, 4, 644. https://doi.org/10.24331/ijere.628470 | 10. Educational-related but it is not about reading |
| Adiani, D., Lewis, D., Serao, V., Barrett, K., Bennett, A., Hambly, D., Shenoda, M., West, S., Coulter, G., Shagal, S., Biala, T., Sarkar, M., Wade, J., & Sarkar, N. (2019). Design of a Novel Web Utility that Provides Multi-lingual Word Definitions for Child E-Book Applications. Lecture Notes in Computer Science (Including Subseries Lecture Notes in Artificial Intelligence and Lecture Notes in Bioinformatics), 11591 LNCS, 3–12. https://doi.org/10.1007/978-3-030-21817-1_1 | 5. Technology but it is not neither a training or reading-related |
| Agredo-Delgado V., R. P. H. (Ed.). (2019). 4th Iberoamerican Workshop on Human-Computer Interaction, HCI-Collab 2018. Communications in Computer and Information Science, 847. https://www.scopus.com/inward/record.uri?eid=2-s2.0-85058539745&partnerID=40&md5=d927d9d26c952b122af8091c61870873 | 11. Type of study excluded (review, non-primary references…) |
| Aguilar, M. (2017). Read, Then Write. Counterman, 35(1), 46. Retrieved from http://ezproxy.si.unav.es:2048/login?url=http://search.ebscohost.com/login.aspx?direct=true&AuthType=ip,url&db=edb&AN=120944356&lang=es&site=eds-live&scope=site | 4. Reading-related but it is not neither a training or technological |
| Aharonson, V., Aharonson, E., Raichlin-Levi, K., Sotzianu, A., Amir, O., & Ovadia-Blechman, Z. (2017). A real-time phoneme counting algorithm and application for speech rate monitoring. Journal of Fluency Disorders, 51, 60–68. https://doi.org/10.1016/j.jfludis.2017.01.001 | 2. It is a reading-related technology but it is not a training (test, support…) |
| Ahmed, S. T. S. (2019). Chat and learn: Effectiveness of using whatsapp as a pedagogical tool to enhance efl learners’ reading and writing skills. International Journal of English Language and Literature Studies, 8(2), 61–68. https://doi.org/10.18488/journal.23.2019.82.61.68 | 9. Training oriented to a second-language learning |
| Ahn, D.-K., Cheong, Y.-G., & Bae, B.-C. (2019). Design Considerations for Developing a Mobile Storytelling Game Application. Communications in Computer and Information Science, 1088, 3–8. https://doi.org/10.1007/978-3-030-30712-7_1 | 2. It is a reading-related technology but it is not a training (test, support…) |
| Ahn, J. Y., & Han, K. S. (2017). Exploring the Shape of Digital Textbook for the Classroom in the Mobile Age. In Tian, F and Gatzidis, C and ElRhalibi, A and Tang, W and Charles, F (Ed.), E-LEARNING AND GAMES, EDUTAINMENT 2017 (Vol. 10345, pp. 299–302). https://doi.org/10.1007/978-3-319-65849-0_34 | 2. It is a reading-related technology but it is not a training (test, support…) |
| Aitchanov, B., Nussipbekov, A., & Zhaparov, M. (2012). Microlearning of web fundamentals based on mobilelearning. IJCSI Press. Retrieved from http://ezproxy.si.unav.es:2048/login?url=http://search.ebscohost.com/login.aspx?direct=true&AuthType=ip,url&db=edsair&AN=edsair.doajarticles..8a6f255cc29c64955413bf003662f422&lang=es&site=eds-live&scope=site | 10. Educational-related but it is not about reading |
| Alali, I. K. (2015). Investigating University Instructors’ Experiences And Uses Of Mobile Technology In Teaching And Learning In Saudi Arabia. Journal of Chemical Information and Modeling. ProQuest Information & Learning, US. https://doi.org/10.1017/CBO9781107415324.004 | 5. Technology but it is not neither a training or reading-related |
| Alaqeel, A., & Aldoghmy, O. (2018). A LITERATURE REVIEW ON EFFECTIVENESS OF COMPUTERIZED TRAINING PROGRAMS ON WORKING MEMORY CAPACITY AND READING ABILITY OF STUDENTS WITH DISABILITIES. https://ezproxy.unav.es/login?url=https://search.ebscohost.com/login.aspx?direct=true&AuthType=ip,url&db=edsbas&AN=edsbas.18D9DAE4&lang=es&site=eds-live&scope=site | 11. Type of study excluded (review, non-primary references…) |
| Al-Barhamtoshy, H., Abdou, S., & Rashwan, M. (2014). Mobile technology for illiterate education. Life Science Journal, 11(9), 242–248. Retrieved from https://www.scopus.com/inward/record.uri?eid=2-s2.0-84901754958&partnerID=40&md5=173497904fac507cc69bedb9d8eb5351 | 2. It is a reading-related technology but it is not a training (test, support…) |
| Alejandro Quezada-Sarmiento, P., Espinoza, V., Ulehlova, E., & Enciso-Quispe, L. (2017). Application of cloud, knowledge and innovation model for improving linguistic in writing skill. 2017 12TH IBERIAN CONFERENCE ON INFORMATION SYSTEMS AND TECHNOLOGIES (CISTI). | 3. Technological training that is not focus on improving reading |
| Alghabban, W. G., Salama, R. M., & Altalhi, A. H. (2017). Mobile cloud computing: An effective multimodal interface tool for students with dyslexia. Computers in Human Behavior, 75, 160-166. https://doi.org/10.1016/j.chb.2017.05.014 | 2. It is a reading-related technology but it is not a training (test, support…) |
| Alghabban, W. G., Salama, R. M., & Altalhi, A. H. (2017). Mobile cloud computing: An effective multimodal interface tool for students with dyslexia. Computers in Human Behavior, 75, 160–166. https://doi.org/10.1016/j.chb.2017.05.014 | 2. It is a reading-related technology but it is not a training (test, support…) |
| Allington, R. L. (2011). What At-Risk Readers Need. Educational Leadership, 68(6), 40–45. Retrieved from http://search.ebscohost.com/login.aspx?direct=true&db=aph&AN=58688610&site=ehost-live | 4. Reading-related but it is not neither a training or technological |
| Alobaydi, E. K., Mustaffa, N., Alkhayat, R. Y., & Arshad, M. R. H. M. (2017). U-Arabic: Design perspective of context-aware ubiquitous Arabic vocabularies learning system. Proceedings - 6th IEEE International Conference on Control System, Computing and Engineering, ICCSCE 2016, 1–6. https://doi.org/10.1109/ICCSCE.2016.7893535 | 7. Technological reading training that do not fulfill methods requirements |
| Al-Razgan, M. S. (2017). Afaneen: The Design and Evaluation of an Interactive Mobile Game to Enhance Arabic Spelling. JOURNAL OF UNIVERSAL COMPUTER SCIENCE, 23(12), 1172–1199. | 7. Technological reading training that do not fulfill methods requirements |
| Alsaif, A., Albadrani, N., Alamro, A., & Alsaif, R. (2017). Towards intelligent Arabic text-to-speech application for disabled people. 2017 International Conference on Informatics, Health and Technology, ICIHT 2017. https://doi.org/10.1109/ICIHT.2017.7899133 | 8. Training oriented to special population (other than dyslexia) |
| Alsaif, A., Albadrani, N., Alamro, A., & Alsaif, R. (2017). Towards intelligent Arabic text-to-speech application for disabled people. 2017 International Conference on Informatics, Health and Technology, ICIHT 2017. https://doi.org/10.1109/ICIHT.2017.7899133 | 8. Training oriented to special population (other than dyslexia) |
| Alshumaimeri, Y. A., & Almasri, M. M. (2012). The effects of using WebQuests on reading comprehension performance of Saudi EFL students. Turkish Online Journal of Educational Technology, 11(4), 295–306. Retrieved from http://ezproxy.si.unav.es:2048/login?url=http://search.ebscohost.com/login.aspx?direct=true&AuthType=ip,url&db=psyh&AN=2013-28852-028&lang=es&site=eds-live&scope=site | 9. Training oriented to a second-language learning |
| Alsunaidi, N., Alzeer, L., Alkatheiri, M., Habbabah, A., Alattas, M., Aljabri, M., & Altassan, M. (2018). Abjad: Towards Interactive Learning Approach to Arabic Reading Based on Speech Recognition. In S. K. El-Beltagy S.R. (Ed.), Procedia Computer Science (Vol. 142, pp. 198–205). Elsevier B.V. https://doi.org/10.1016/j.procs.2018.10.476 | 7. Technological reading training that do not fulfill methods requirements |
| Alsunaidi, N., Alzeer, L., Alkatheiri, M., Habbabah, A., Alattas, M., Aljabri, M., & Altassan, M. (2018). Abjad: Towards Interactive Learning Approach to Arabic Reading Based on Speech Recognition. In S. K. El-Beltagy S.R. (Ed.), Procedia Computer Science (Vol. 142, pp. 198–205). Elsevier B.V. https://doi.org/10.1016/j.procs.2018.10.476 | 5. Technology but it is not neither a training or reading-related |
| Alt, M., Hogan, T., Green, S., Gray, S., Cabbage, K., & Cowan, N. (2017). Word learning deficits in children with dyslexia. Journal of Speech, Language, and Hearing Research, 60(4), 1012-1028. https://doi.org/10.1044/2016_JSLHR-L-16-0036 | 2. It is a reading-related technology but it is not a training (test, support…) |
| Alt, M., Hogan, T., Green, S., Gray, S., Cabbage, K., & Cowan, N. (2017). Word learning deficits in children with dyslexia. Journal of Speech, Language, and Hearing Research, 60(4), 1012–1028. https://doi.org/10.1044/2016_JSLHR-L-16-0036 | 4. Reading-related but it is not neither a training or technological |
| Amanda, M. (2012). Research-based design of a medical vocabulary videogame. International Journal of Pedagogies and Learning, 7(2), 122-134. https://doi.org/10.5172/ijpl.2012.7.2.122 | 9. Training oriented to a second-language learning |
| Amelia, L. C. H., & Abidin, M. J. Z. (2018). Young ESL learners’ perception on the effects of using digital storytelling application in English language learning. Pertanika Journal of Social Sciences and Humanities, 26(T), 179–198. https://www.scopus.com/inward/record.uri?eid=2-s2.0-85061127585&partnerID=40&md5=a672b43a56e87569df423a8c8ef8eb2d | 9. Training oriented to a second-language learning |
| Amelia, L. C. H., & Abidin, M. J. Z. (2018). Young ESL learners’ perception on the effects of using digital storytelling application in English language learning. Pertanika Journal of Social Sciences and Humanities, 26(T), 179–198. https://www.scopus.com/inward/record.uri?eid=2-s2.0-85061127585&partnerID=40&md5=a672b43a56e87569df423a8c8ef8eb2d | 9. Training oriented to a second-language learning |
| An, H., Alon, S., & Fuentes, D. (2014). Tablets in K-12 education: Integrated experiences and implications. Tablets in K-12 Education: Integrated Experiences and Implications. IGI Global. https://doi.org/10.4018/978-1-4666-6300-8 | 3. Technological training that is not focus on improving reading |
| Andreasen, H. (2009). Combining Comprehension Reading Instruction with Video Anchors with Middle Level Learners. ProQuest LLC. ProQuest Information & Learning, US. Retrieved from http://proxy.lib.uiowa.edu/login?url=http://search.ebscohost.com/login.aspx?direct=true&db=eric&AN=ED532877&site=ehost-live%5Cnhttp://gateway.proquest.com/openurl?url_ver=Z39.88-2004&rft_val_fmt=info:ofi/fmt:kev:mtx:dissertation&res_dat=xri:pqdiss&rft_dat | 1.Non technological reading training |
| Andreev, R., Terzieva, V., & Kademova-Katzarova, P. (2009). An approach to development of personalized e-learning environment for dyslexic pupils’ acquisition of reading competence. In Proceedings of the International Conference on Computer Systems and Technologies and Workshop for PhD Students in Computing - CompSysTech ’09 (Vol. 433, p. 1). Institute of Computer and Communication Systems, Bulgarian Academy of Sciences, Bulgaria. https://doi.org/10.1145/1731740.1731829 | 2. It is a reading-related technology but it is not a training (test, support…) |
| Angelidis, G., Valotassiou, V., Alexiou, S., Tsougos, I., Psimadas, D., Tzavara, C., Ziaka, A., Baniora, E., Theodorou, E., Ziangas, C., Tzitzani, A., Kournouti, M., Asproudi, M., & Georgoulias, P. (2019). Comparison between computer-based analysis and expert reading in the interpretation of MPI studies. EUROPEAN JOURNAL OF NUCLEAR MEDICINE AND MOLECULAR IMAGING, 46(SUPPL 1, 1, SI), S347. | 5. Technology but it is not neither a training or reading-related |
| Archibald, L. M. D., Orange, J. B., & Jamieson, D. J. (2009). Implementation of computer-based language therapy in aphasia. Therapeutic Advances in Neurological Disorders, 2(5), 299-311. https://doi.org/10.1177/1756285609336548 | 8. Training oriented to special population (other than dyslexia) |
| Arena, D. A., & Schwartz, D. L. (2014). Experience and explanation: Using videogames to prepare students for formal instruction in statistics. Journal of Science Education and Technology, 23(4), 538-548. https://doi.org/10.1007/s10956-013-9483-3 | 3. Technological training that is not focus on improving reading |
| Arifani, Y. (2018). The Effectiveness of Pamanpintermu E-reading Program on EFL Learners’ Reading Performances. In Inam, A and Sayogo, DS and Widayat and Isomudin and Latipun and Zulfatman (Ed.), PROCEEDINGS OF THE 5TH INTERNATIONAL CONFERENCE ON COMMUNITY DEVELOPMENT (AMCA 2018) (Vol. 231, pp. 21–24). | 9. Training oriented to a second-language learning |
| Arikan, A., & Khezerlou, E. (2010). Prospective English language teachers’ views on computer and paper-based instructional materials in developing language components. Procedia - Social and Behavioral Sciences, 2(2), 4006-4009. https://doi.org/10.1016/j.sbspro.2010.03.631 | 5. Technology but it is not neither a training or reading-related |
| Arnbak, E., & Elbro, C. (2000). The Effects of Morphological Awareness Training on the Reading and Spelling Skills of Young Dyslexics. Scandinavian Journal of Educational Research. https://doi.org/10.1080/00313830050154485 | 1.Non technological reading training |
| Aroca, R. V., Burlamaqui, A. F., & Gonçalves, L. M. G. (2012). Method for reading sensors and controlling actuators using audio interfaces of mobile devices. Sensors, 12(2), 1572–1593. https://doi.org/10.3390/s120201572 | 5. Technology but it is not neither a training or reading-related |
| Arrieta, A. M. (2012). Transmedia : A proposal for the production of educational contents. Kepes, (8), 199–220. Retrieved from http://search.ebscohost.com.ezproxybib.pucp.edu.pe:2048/login.aspx?direct=true&db=fua&AN=110231880&lang=es&site=ehost-live | 10. Educational-related but it is not about reading |
| Arteaga, J. M., & Pinedo Rivera, D. I. (2019). A process model to develop educational applications for children with dyslexia. In G. H. G. P. R. B. L. F. J. S. C. J. A. G. C. A. G. R. S. V Juarez-Ramirez R. Fernandez y Fernandez C. (Ed.), Proceedings - 2018 6th International Conference in Software Engineering Research and Innovation, CONISOFT 2018 (pp. 79–87). Institute of Electrical and Electronics Engineers Inc. https://doi.org/10.1109/CONISOFT.2018.8645896 | 11. Type of study excluded (review, non-primary references…) |
| Arteaga, J. M., & Pinedo Rivera, D. I. (2019). A process model to develop educational applications for children with dyslexia. In G. H. G. P. R. B. L. F. J. S. C. J. A. G. C. A. G. R. S. V Juarez-Ramirez R. Fernandez y Fernandez C. (Ed.), Proceedings - 2018 6th International Conference in Software Engineering Research and Innovation, CONISOFT 2018 (pp. 79–87). Institute of Electrical and Electronics Engineers Inc. https://doi.org/10.1109/CONISOFT.2018.8645896 | 11. Type of study excluded (review, non-primary references…) |
| Arvanitis, P., Krystalli, P., & Panagiotidis, P. (2016). Applicatons for Mobile Assisted Language Learning: A current field research. In Chova, LG and Martinez, AL and Torres, IC (Ed.), The 10th annual International Technology, Education and Development Conference (pp. 7645–7651). | 9. Training oriented to a second-language learning |
| ASSETS 2017 - Proceedings of the 19th International ACM SIGACCESS Conference on Computers and Accessibility. (2017). ASSETS 2017 - Proceedings of the 19th International ACM SIGACCESS Conference on Computers and Accessibility. https://www.scopus.com/inward/record.uri?eid=2-s2.0-85041393588&partnerID=40&md5=665656c31a534f9ed2f00538b225694e | 11. Type of study excluded (review, non-primary references…) |
| Asutay, H. (2017). New Types of Writing Developed Via New Media. In Pereira, PAD and Titrek, O and SezenGultekin, G (Ed.), 3RD INTERNATIONAL CONFERENCE ON LIFELONG LEARNING AND LEADERSHIP FOR ALL (ICLEL 2017) (pp. 232–237). | 2. It is a reading-related technology but it is not a training (test, support…) |
| Atkins, A. (2013). An Investigation into Multi-level Components of Online Reading Fluency. Research-publishing.net. Research-publishing.net. Retrieved from http://ezproxy.si.unav.es:2048/login?url=http://search.ebscohost.com/login.aspx?direct=true&AuthType=ip,url&db=eric&AN=ED565085&lang=es&site=eds-live&scope=site | 4. Reading-related but it is not neither a training or technological |
| Auer, N. (2014). Promoting Strategic Reading Using the iBooks Author Application. In Advancing Higher Education with Mobile Learning Technologies: Cases, Trends, and Inquiry-Based Methods (pp. 179–194). Institute of Learning Innovation, University of Leicester, United Kingdom: IGI Global. https://doi.org/10.4018/978-1-4666-6284-1.ch010 | 2. It is a reading-related technology but it is not a training (test, support…) |
| Auer, N. (2014). Reading on tablets :  Students awareness and use of foreign language reading strategies. None;  CO  - Awareness#2; Book#1; Classroom#1; Cognitive#1; Colloquium#1; Comprehension#4; Conference#1; E Book#5; Fl#1; Foreign Language#1; Ibook#1; Infer#1; Interview#1; Ipad#1; Language#2; Learner#6; Log#5; Metacognitive#1; Metacognitive Think#1; Mobil, 624. Retrieved from http://ezproxy.si.unav.es:2048/login?url=http://search.ebscohost.com/login.aspx?direct=true&AuthType=ip,url&db=edb&AN=99239735&lang=es&site=eds-live&scope=site | 2. It is a reading-related technology but it is not a training (test, support…) |
| Auer, N. (2016). Scaffolding foreign language learners’ reading strategies using tablet computers at two secondary schools in Denmark. University of Leicester. Retrieved from http://ezproxy.si.unav.es:2048/login?url=http://search.ebscohost.com/login.aspx?direct=true&AuthType=ip,url&db=edsble&AN=edsble.700937&lang=es&site=eds-live&scope=site | 9. Training oriented to a second-language learning |
| Auphan, P. ( 1 ), Ecalle, J. ( 1 ), Magnan, A., Magnan 2 ), A. ( 1, & Magnan, A. (2018). Computer-based assessment of reading ability and subtypes of readers with reading comprehension difficulties: a study in French children from G2 to G9. European Journal of Psychology of Education, 34(3), 641–663. https://doi.org/10.1007/s10212-018-0396-7 | 2. It is a reading-related technology but it is not a training (test, support…) |
| Ávila Clemente, V., Gil Pelluch, L., Gilabert, R., Maña, A., & Vidal-Abarca Gamez, E. (2016). ‘Método de Evaluación Dinámica Automatizado’ de Competencias Lectoras para Educación Secundaria. (EdiLEC). Universitas Psychologica, 15(1), 219-232. https://doi.org/10.11144/Javeriana.upsy15-1.meda | 2. It is a reading-related technology but it is not a training (test, support…) |
| Avishka, I., Kumarawadu, K., Kudagama, A., Weerathunga, M., & Thelijjagoda, S. (2018). Mobile App to Support People with Dyslexia and Dysgraphia. 2018 IEEE 9th International Conference on Information and Automation for Sustainability, ICIAfS 2018. https://doi.org/10.1109/ICIAFS.2018.8913335 | 7. Technological reading training that do not fulfill methods requirements |
| Avtzon, S. A. (2007). Effect of Neuroscience-based Cognitive skill Training on Growth of Cognitive Deficits Associated with Learning Disabilities in Children Grades 2-4. Learning Disabilities. ProQuest Information & Learning, US. Retrieved from http://ezproxy.si.unav.es:2048/login?url=http://search.ebscohost.com/login.aspx?direct=true&AuthType=ip,url&db=psyh&AN=2013-99111-397&lang=es&site=eds-live&scope=site | 3. Technological training that is not focus on improving reading |
| Azmi, A. Z., Nasrudin, N. H., Wan, A. W. N., & Ahmad, J. R. (2017). MOBILE APPLICATION TO ENHANCE WRITING SKILLS ABILITY AMONG DYSLEXIC CHILDREN: CiNTA. JOURNAL OF FUNDAMENTAL AND APPLIED SCIENCES, 9(5, SI), 195–209. https://doi.org/10.4314/jfas.v9i5s.15 | 3. Technological training that is not focus on improving reading |
| Bachtiar, F. A., Sulistyo, G. H., Cooper, E. W., & Katsuari, K. (2017). Affect, Personality, and Learning Styles in Online Reading Comprehension. En Proceedings of the 5th International Conference on Information and Education Technology (pp. 78-83). Faculty of Computer Science, Brawijaya University, East-Java, Indonesia: Association for Computing Machinery. https://doi.org/10.1145/3029387.3029422 | 2. It is a reading-related technology but it is not a training (test, support…) |
| Bachtiar, F. A., Sulistyo, G. H., Cooper, E. W., & Katsuari, K. (2017). Affect, Personality, and Learning Styles in Online Reading Comprehension. ICIET’17: PROCEEDINGS OF THE 5TH INTERNATIONAL CONFERENCE ON INFORMATION AND EDUCATION TECHNOLOGY, 78–83. https://doi.org/10.1145/3029387.3029422 | 2. It is a reading-related technology but it is not a training (test, support…) |
| Bachtiar, F. A., Sulistyo, G. H., Cooper, E. W., & Katsuari, K. (2017). Affect, Personality, and Learning Styles in Online Reading Comprehension. ICIET’17: PROCEEDINGS OF THE 5TH INTERNATIONAL CONFERENCE ON INFORMATION AND EDUCATION TECHNOLOGY, 78–83. https://doi.org/10.1145/3029387.3029422 | 2. It is a reading-related technology but it is not a training (test, support…) |
| Badia, A. (2015). Research trends in technology- enhanced learning / Tendencias de la investigación en el aprendizaje favorecido por la tecnología. Infancia y Aprendizaje/Journal for the Study of Education and Development, 38(2), 253–278. https://doi.org/10.1080/02103702.2015.1016744 | 3. Technological training that is not focus on improving reading |
| Bailloud, N., Auphan, P., Magnan, A., & Ecalle, J. (2017). COMPUTER-BASED ASSESSMENT OF READING IN 7-TO 10 Y-O CHILDREN: THE ROLE OF VOCABULARY IN WORD READING AND COMPREHENSION. In Chova, LG and Martinez, AL and Torres, IC (Ed.), 9TH INTERNATIONAL CONFERENCE ON EDUCATION AND NEW LEARNING TECHNOLOGIES (EDULEARN17) (pp. 421–426). | 2. It is a reading-related technology but it is not a training (test, support…) |
| Bamasag, O., Tayeb, M., Alsaggaf, M., & Shams, F. (2018). Nateq reading arabic text for visually impaired people. Lecture Notes in Computer Science (Including Subseries Lecture Notes in Artificial Intelligence and Lecture Notes in Bioinformatics), 10907 LNCS, 311–326. https://doi.org/10.1007/978-3-319-92049-8_23 | 8. Training oriented to special population (other than dyslexia) |
| Bamidis P.D. Konstantinidis S.Th., R. P. P. (Ed.). (2017). Proceedings - IEEE Symposium on Computer-Based Medical Systems. In Proceedings - IEEE Symposium on Computer-Based Medical Systems (Vols. 2017-June). Institute of Electrical and Electronics Engineers Inc. https://www.scopus.com/inward/record.uri?eid=2-s2.0-85040334293&partnerID=40&md5=39aab0fa352a5ffb9754daab883ed8e8 | 11. Type of study excluded (review, non-primary references…) |
| Banerjee, A., Chishti, M. S., & Kumar, G. (2017). On Exploring NFC for Half-Duplex Communication in Read/Write Mode. 2017 INTERNATIONAL CONFERENCE ON SELECTED TOPICS IN MOBILE AND WIRELESS NETWORKING (MOWNET), 62–69. | 5. Technology but it is not neither a training or reading-related |
| Barnes, D. E., Yaffe, K., Belfor, N., Jagust, W. J., DeCarli, C., Reed, B. R., & Kramer, J. H. (2009). Computer-based Cognitive Training for Mild Cognitive Impairment Results from a Pilot Randomized, Controlled Trial. Alzheimer Disease & Associated Disorders, 23(3), 205-210. https://doi.org/10.1097/WAD.0b013e31819c6137 | 8. Training oriented to special population (other than dyslexia) |
| Barsukova, A. A., & Aksenova, V. Y. (2018). Challenges for Russian students in computer-based tests. In I. P. I. D. I. D. R. L. Sampson D.G. Sampson D.G. (Ed.), Proceedings of the 15th International Conference on Cognition and Exploratory Learning in the Digital Age, CELDA 2018 (pp. 401–403). IADIS Press. https://www.scopus.com/inward/record.uri?eid=2-s2.0-85060304715&partnerID=40&md5=298ba8306caa76e7e39867864b6671fd | 5. Technology but it is not neither a training or reading-related |
| Bauer-Kealey, M., & Mather, N. (2019). Use of an Online Reading Intervention to Enhance the Basic Reading Skills of Community College Students. COMMUNITY COLLEGE JOURNAL OF RESEARCH AND PRACTICE, 43(9), 631–647. https://doi.org/10.1080/10668926.2018.1524335 | 7. Technological reading training that do not fulfill methods requirements |
| Bax, S. (2013). The cognitive processing of candidates during reading tests: Evidence from eye-tracking. Language Testing. https://doi.org/10.1177/0265532212473244 | 2. It is a reading-related technology but it is not a training (test, support…) |
| Beavis, C. (2014). Games as text, games as action: Video games in the english classroom. Journal of Adolescent and Adult Literacy, 57(6), 433–439. https://doi.org/10.1002/jaal.275 | 9. Training oriented to a second-language learning |
| Bederson, B. B., Quinn, A., & Druin, A. (2009). Designing the reading experience for scanned multi-lingual picture books on mobile phones. En 2009 ACM/IEEE Joint Conference on Digital Libraries, JCDL’09 (pp. 305-308). University of Maryland Human, Computer Interaction Lab, United States. https://doi.org/10.1145/1555400.1555450 | 2. It is a reading-related technology but it is not a training (test, support…) |
| Beer, W., & Wagner, A. (2011). Smart books. In Proceedings of the 9th International Conference on Advances in Mobile Computing and Multimedia - MoMM ’11 (p. 218). Software Competence Center Hagenberg, Softwarepark 21, Hagenberg, Austria. https://doi.org/10.1145/2095697.2095740 | 2. It is a reading-related technology but it is not a training (test, support…) |
| Belen, G. M. A., Cabonita, K. B. P., dela Pena, V. A., Dimakuta, H. A., Hoyle, F. G., & Laviste, R. P. (2018). Tingog: Reading and Speech Application for Children with Repaired Cleft Palate. 2018 IEEE 10TH INTERNATIONAL CONFERENCE ON HUMANOID, NANOTECHNOLOGY, INFORMATION TECHNOLOGY, COMMUNICATION AND CONTROL, ENVIRONMENT AND MANAGEMENT (HNICEM). | 8. Training oriented to special population (other than dyslexia) |
| Belen, G. M. A., Cabonita, K. B. P., dela Pena, V. A., Dimakuta, H. A., Hoyle, F. G., & Laviste, R. P. (2018). Tingog: Reading and Speech Application for Children with Repaired Cleft Palate. 2018 IEEE 10TH INTERNATIONAL CONFERENCE ON HUMANOID, NANOTECHNOLOGY, INFORMATION TECHNOLOGY, COMMUNICATION AND CONTROL, ENVIRONMENT AND MANAGEMENT (HNICEM). | 8. Training oriented to special population (other than dyslexia) |
| Bellingham, H. S. (2014). the Integration of a Computer-Based Early Reading Program To Increase English Language Learners ’ Literacy Skills. Teaching English with Technology, 14(1), 9–22. Retrieved from http://ezproxy.si.unav.es:2048/login?url=http://search.ebscohost.com/login.aspx?direct=true&AuthType=ip,url&db=eric&AN=EJ1143396&lang=es&site=eds-live&scope=site | 9. Training oriented to a second-language learning |
| Belo, N. A. H., McKenney, S. E., & Voogt, J. M. (2013). Towards a knowledge base for using ICT to foster early literacy development: A review study. Retrieved from http://ezproxy.si.unav.es:2048/login?url=http://search.ebscohost.com/login.aspx?direct=true&AuthType=ip,url&db=edsair&AN=edsair.od.......233..e6687bdfa97c4490e79a389315ca7928&lang=es&site=eds-live&scope=site | 11. Type of study excluded (review, non-primary references…) |
| Belo, N., McKenney, S., Voogt, J., & Bradley, B. (2016). Teacher knowledge for using technology to foster early literacy: A literature review. Computers in Human Behavior, 60, 372-383. https://doi.org/10.1016/j.chb.2016.02.053 | 11. Type of study excluded (review, non-primary references…) |
| Benalcazar Chicaiza, D., Barrera I, M., Paez Quinde, M. C., & Pilamunga, M. E. (2019). M-Learning Didactic Strategy for Children Diagnosed with Dyslexia. In Antipova, T and Rocha, A (Ed.), DIGITAL SCIENCE (Vol. 850, pp. 143–149). https://doi.org/10.1007/978-3-030-02351-5_18 | 3. Technological training that is not focus on improving reading |
| Benalcazar Chicaiza, D., Barrera I, M., Paez Quinde, M. C., & Pilamunga, M. E. (2019). M-Learning Didactic Strategy for Children Diagnosed with Dyslexia. In Antipova, T and Rocha, A (Ed.), DIGITAL SCIENCE (Vol. 850, pp. 143–149). https://doi.org/10.1007/978-3-030-02351-5_18 | 3. Technological training that is not focus on improving reading |
| Bengoechea, J. I. I. (2009). Videogames and education: A first empirical research in the basque country. In Pivec, M (Ed.), Proceedings of the European Conference on Games-based Learning (Vol. 2009-Janua, pp. 195–201). Retrieved from https://www.scopus.com/inward/record.uri?eid=2-s2.0-79959441572&partnerID=40&md5=4e63d8bc79297a2e551b9ea43e270948 | 10. Educational-related but it is not about reading |
| Benmarrakchi, F. E., El Kafi, J., & Elhore, A. (2017). User Modeling Approach for Dyslexic Students in Virtual Learning Environments. International Journal of Cloud Applications and Computing, 7(2, 2, SI), 1-9. https://doi.org/10.4018/IJCAC.2017040101 | 2. It is a reading-related technology but it is not a training (test, support…) |
| Benmarrakchi, F. E., El Kafi, J., & Elhore, A. (2017). User Modeling Approach for Dyslexic Students in Virtual Learning Environments. INTERNATIONAL JOURNAL OF CLOUD APPLICATIONS AND COMPUTING, 7(2, 2, SI), 1–9. https://doi.org/10.4018/IJCAC.2017040101 | 2. It is a reading-related technology but it is not a training (test, support…) |
| Berger, R., & McDougall, J. (2012). Digital Transformations: Games As (Authorless) Novels in the English Literature Curriculum. En Chova, LG and Martinez, AL and Torres, IC (Ed.), 5Th International Conference of Education, Research and Innovation (Iceri 2012) (pp. 817-823). | 5. Technology but it is not neither a training or reading-related |
| Berger, R., & Mcdougall, J. (2013). Reading videogames as (authorless) literature. Literacy. https://doi.org/10.1111/lit.12004 | 5. Technology but it is not neither a training or reading-related |
| Berkowitz, T., Schaeffer, M. W., Rozek, C. S., Maloney, E. A., Levine, S. C., & Beilock, S. L. (2016). Response to Comment on «Math at home adds up to achievement in school». Science, 351(6278), 1161-1161. https://doi.org/10.1126/science.aad8555 | 3. Technological training that is not focus on improving reading |
| Bertumen, E., Antonio Albornoz, C., Rae Canero, D., Manalo, J., & Ramos, A. (2017). FLIPPINO: A MOBILE APPLICATION ON READING FOR THE DYSLEXIC STUDENTS OF WORDLAB. In Chova, LG and Martinez, AL and Torres, IC (Ed.), 9TH INTERNATIONAL CONFERENCE ON EDUCATION AND NEW LEARNING TECHNOLOGIES (EDULEARN17) (pp. 8117–8126). | 7. Technological reading training that do not fulfill methods requirements |
| Besoain, F., Perez-Navarro, A., Caylà, J. A., Aviñó, C. J., & de Olalla, P. G. (2015). Prevention of sexually transmitted infections using mobile devices and ubiquitous computing. International Journal of Health Geographics, 14(1), 18. https://doi.org/10.1186/s12942-015-0010-z | 10. Educational-related but it is not about reading |
| Biersdorfer, J. D. (2017, May 14). Serial Fiction on Tap. New York Times Book Review. Retrieved from http://ezproxy.si.unav.es:2048/login?url=http://search.ebscohost.com/login.aspx?direct=true&AuthType=ip,url&db=asx&AN=123036385&lang=es&site=eds-live&scope=site | 3. Technological training that is not focus on improving reading |
| Bigotte, E., Vasconcelos, V., Pires, S., & Fonseca, T. (2016). Executive function assessment in Parkinson’s disease patients using mobile devices. In R. A., R. L.P., C. M.P., G. R., & S. O.S. (Eds.), 11th Iberian Conference on Information Systems and Technologies, CISTI 2016 (Vol. 2016-July). Coimbra Institute of Engineering, Polytechnic Institute of Coimbra, CASPAE, Coimbra, Portugal: IEEE Computer Society. https://doi.org/10.1109/CISTI.2016.7521474 | 8. Training oriented to special population (other than dyslexia) |
| Bippert, K., & Harmon, J. (2017). Middle School Teachers’ Perceptions of Computer-Assisted Reading Intervention Programs. Reading Psychology. ProQuest Information & Learning, US. https://doi.org/10.1080/02702711.2016.1245691 | 2. It is a reading-related technology but it is not a training (test, support…) |
| Bittencourt, T., Savino, J., Fernandes, H., & Rebello, L. H. B. (2016). Mobile device development and its contribution to the treatment of young dyslexic Brazilian children. ( di B. G. & K. P., Eds.), Advances in Intelligent Systems and Computing. Universidade Federal Fluminense, GDI, TDT, Escola de Engenharia, Passos da Pátria 156, Bloco D, Sala 201, Niterói, RJ, Brazil: Springer Verlag. https://doi.org/10.1007/978-3-319-41962-6_30 | 7. Technological reading training that do not fulfill methods requirements |
| Björn, P. M., & Leppänen, P. H. T. (2013). Accelerating decoding-related skills in poor readers learning a foreign language: A computer-based intervention. Educational Psychology. https://doi.org/10.1080/01443410.2013.797336 | 9. Training oriented to a second-language learning |
| Black, M. P., Skinner, C. H., Forbes, B. E., McCurdy, M., Coleman, M. B., Davis, K., & Gettelfinger, M. (2016). Cumulative Instructional Time and Relative Effectiveness Conclusions: Extending Research on Response Intervals, Learning, and Measurement Scale. Behavior Analysis in Practice, 9(1), 58–62. https://doi.org/10.1007/s40617-016-0114-3 | 8. Training oriented to special population (other than dyslexia) |
| Blakesley, E. (2015). Reading the Fine Print. Journal of Academic Librarianship, 41(6), 711. https://doi.org/10.1016/j.acalib.2015.10.010 | 4. Reading-related but it is not neither a training or technological |
| Bloomfield, J., Fordham-Clarke, C., Pegram, A., & Cunningham, B. (2010). The development and evaluation of a computer-based resource to assist pre-registration nursing students with their preparation for objective structured clinical examinations (OSCEs). Nurse Education Today, 30(2), 113–117. https://doi.org/10.1016/j.nedt.2009.06.004 | 10. Educational-related but it is not about reading |
| Blything, L. P., Hardie, A., & Cain, K. (2019). Question Asking During Reading Comprehension Instruction: A Corpus Study of How Question Type Influences the Linguistic Complexity of Primary School Students’ Responses. Reading Research Quarterly. https://doi.org/10.1002/rrq.279 | 11. Type of study excluded (review, non-primary references…) |
| Bobian, M., Kandinov, A., El-Kashlan, N., Svider, P. F., Folbe, A. J., Mayerhoff, R., … Raza, S. N. (2017). Mobile applications and patient education: Are currently available GERD mobile apps sufficient? Laryngoscope, 127(8), 1775–1779. https://doi.org/10.1002/lary.26341 | 10. Educational-related but it is not about reading |
| Bobian, M., Kandinov, A., El-Kashlan, N., Svider, P. F., Folbe, A. J., Mayerhoff, R., Eloy, J. A., & Raza, S. N. (2017). Mobile Applications and Patient Education: Are Currently Available GERD Mobile Apps Sufficient? LARYNGOSCOPE, 127(8), 1775–1779. https://doi.org/10.1002/lary.26341 | 5. Technology but it is not neither a training or reading-related |
| Boboc, A.-L., Orzan, G., Stoica, I., & Niculescu-Ciocan, C. (2018). Gamification and Game-Based Learning - a Solution for Romanian Education System? In Roceanu, I and Beligan, D and Ciolan, L and Stefan, I (Ed.), ELEARNING CHALLENGES AND NEW HORIZONS, VOL 1 (pp. 242–248). https://doi.org/10.12753/2066-026X-18-033 | 5. Technology but it is not neither a training or reading-related |
| Boon, R. T., & Barbetta, P. M. (2017). Reading Interventions for Elementary English Language Learners With Learning Disabilities: A Review. Insights on Learning Disabilities, 14(1), 27. Retrieved from http://ezproxy.si.unav.es:2048/login?url=http://search.ebscohost.com/login.aspx?direct=true&AuthType=ip,url&db=edo&AN=123788818&lang=es&site=eds-live&scope=site | 11. Type of study excluded (review, non-primary references…) |
| Booth, P. (2013). Educational Space, Place and Mobile Applications. In Chova, LG and Martinez, AL and Torres, IC (Ed.), Edulearn13: 5th International Conference on Education and New Learning Technologies (pp. 6465–6469). | 10. Educational-related but it is not about reading |
| Boratto, L., Cadeddu, M., Carta, S., Deplano, G., & Mereu, F. (2016). A tool to analyze the reading behavior of the users in a mobile digital publishing platform. In A. G., C. M., G. A., & B. A. (Eds.), CEUR Workshop Proceedings (Vol. 1748). Dipartimento di Matematica e Informatica, Università di Cagliari, Italy: CEUR-WS. Retrieved from https://www.scopus.com/inward/record.uri?eid=2-s2.0-85012241655&partnerID=40&md5=a1c17c0683d77405dae61d27ac55ae2a | 2. It is a reading-related technology but it is not a training (test, support…) |
| Borgonovi, F. (2016). Video gaming and gender differences in digital and printed reading performance among 15-year-olds students in 26 countries. Journal of Adolescence, 48, 45–61. https://doi.org/10.1016/j.adolescence.2016.01.004 | 2. It is a reading-related technology but it is not a training (test, support…) |
| Borgstrom, L. (2011). Developing Story-Time: the Importance of Interactivity in Encouraging Childhood Reading. Mousaion, 29(3), 193–208. Retrieved from http://search.ebscohost.com/login.aspx?direct=true&db=lih&AN=97849317&site=ehost-live | 2. It is a reading-related technology but it is not a training (test, support…) |
| Borhan, N. H., Shiang, C. W., Chiu, P.-C., Sharbini, H., Ping, T. P., Othman, R. M., & Peter, M. (2018). An enhancement of dyslexic mobile application using sight word reading strategy: Results and findings. Journal of Computer Science, 14(7), 919–929. https://doi.org/10.3844/jcssp.2018.919.929 | 7. Technological reading training that do not fulfill methods requirements |
| Borleffs, L. E. (2018). De code kraken:Towards understanding, diagnosing and remediating dyslexia in Standard Indonesian ; Cracking the code:Towards understanding, diagnosing and remediating dyslexia in Standard Indonesian. Rijksuniversiteit Groningen. https://ezproxy.unav.es/login?url=https://search.ebscohost.com/login.aspx?direct=true&AuthType=ip,url&db=edsbas&AN=edsbas.D8A6E028&lang=es&site=eds-live&scope=site | 4. Reading-related but it is not neither a training or technological |
| Borodin, Y., Puzis, Y., Soviak, A., Bouker, J., Feng, B., Sicoli, R., … Ramakrishnan, I. V. (2014). Listen to Everything You Want to Read with Capti Narrator. In Proceedings of the 11th Web for All Conference (pp. 33:1--33:2). Charmtech Labs LLC, CEWIT, 1500 Stony Brook Rd., Stony Brook, NY, United States: Association for Computing Machinery. https://doi.org/10.1145/2596695.2596728 | 2. It is a reading-related technology but it is not a training (test, support…) |
| Boteanu, A., & Chernova, S. (2012). Modeling topics in user dialog for interactive tablet media. In AAAI Workshop - Technical Report (Vol. WS-12-17, pp. 2–8). Worcester Polytechnic Institute, United States. Retrieved from https://www.scopus.com/inward/record.uri?eid=2-s2.0-84876021485&partnerID=40&md5=816e0f24545ab147bd782b01cc6cc898 | 5. Technology but it is not neither a training or reading-related |
| Botello, J. A. (2014). Comparing the Effect of Two Types of Computer Screen Background Lighting on Students’ Reading Engagement and Achievement. ProQuest Dissertations and Theses. ProQuest Information & Learning, US. Retrieved from https://search.proquest.com/docview/1530298552?accountid=10673%0Ahttp://openurl.ac.uk/redirect/athens:edu/?url_ver=Z39.88-2004&rft_val_fmt=info:ofi/fmt:kev:mtx:dissertation&genre=dissertations+%26+theses&sid=ProQ:Education+Database&atitle=&title=Comparing | 2. It is a reading-related technology but it is not a training (test, support…) |
| Botzakis, S. (2016). Visual and Digital Texts. Journal of Adolescent and Adult Literacy, 59(6), 731–733. https://doi.org/10.1002/jaal.518 | 2. It is a reading-related technology but it is not a training (test, support…) |
| Bouchardon, S., Clément, J., Réty, J.-H., Szilas, N., & Angé, C. (2008). Rencontre : an experimental tool for electronic literature. Proceedings of the Conference Electronic Literature in Europe. Retrieved from http://ezproxy.si.unav.es:2048/login?url=http://search.ebscohost.com/login.aspx?direct=true&AuthType=ip,url&db=edsair&AN=edsair.od......1400..e5506618d9819fdce1449b1546913fbe&lang=es&site=eds-live&scope=site | 2. It is a reading-related technology but it is not a training (test, support…) |
| Boudo, L., Cavallaro, D., Hurtado, D., Pisano, K. R., Rutkowski, P., Smayda, S., … Chase, P. (2014). Children’s Early Literacy Development and Adults’ Positive Disposition Toward Reading Through E-books and Apps. New England Reading Association Journal, 49(1), 23–32. Retrieved from http://proxygw.wrlc.org/login?url=http://search.ebscohost.com/login.aspx?direct=true&db=eue&AN=102395498&site=ehost-live | 2. It is a reading-related technology but it is not a training (test, support…) |
| Bowers, A. J., & Berland, M. (2013). Does recreational computer use affect high school achievement? Educational Technology Research and Development. https://doi.org/10.1007/s11423-012-9274-1 | 7. Technological reading training that do not fulfill methods requirements |
| Bowles, M. (2017). Leveraging the affordances of mobile learning for vocabulary gains. In I. D. S. D. G. I. P. R. L. Spector J.M. Ifenthaler D. (Ed.), 14th International Conference on Cognition and Exploratory Learning in the Digital Age, CELDA 2017 (pp. 241–245). IADIS Press. https://www.scopus.com/inward/record.uri?eid=2-s2.0-85055970531&partnerID=40&md5=dee87cd848229c48c94407145a253b32 | 7. Technological reading training that do not fulfill methods requirements |
| Boyle, D., Kolcun, R., & Yeatman, E. (2015). Devices in the Internet of Things. Journal of the Institute of Telecommunications Professionals, 9(4), 27–31. Retrieved from https://www.scopus.com/inward/record.uri?eid=2-s2.0-84958766701&partnerID=40&md5=2e2f20c6f0037b4636b9cfa47e1c9daf | 5. Technology but it is not neither a training or reading-related |
| Brand, L., Beltran, A., Buday, R., O’Connor, T., Hughes, S., Baranowski, J., … Baranowski, T. (2015). Prose Fiction as a Narrative Companion for a Vegetable Parenting Videogame. Games for Health Journal, 4(4), 305–311. https://doi.org/10.1089/g4h.2014.0143 | 10. Educational-related but it is not about reading |
| Brantmeier, C., & Vanderplank, R. (2008). Descriptive and criterion-referenced self-assessment with L2 readers. System, 36(3), 456–477. https://doi.org/10.1016/j.system.2008.03.001 | 2. It is a reading-related technology but it is not a training (test, support…) |
| Brantmeier, C., Vanderplank, R., & Strube, M. (2012). What about me?. Individual self-assessment by skill and level of language instruction. System, 40(1), 144–160. https://doi.org/10.1016/j.system.2012.01.003 | 2. It is a reading-related technology but it is not a training (test, support…) |
| Broberg, A. (2008). Text–Col: A Tool for Active Reading. Online and Distance Learning: Concepts, Methodologies, Tools, and Applications VO - 3. IGI Global. Retrieved from http://ezproxy.si.unav.es:2048/login?url=http://search.ebscohost.com/login.aspx?direct=true&AuthType=ip,url&db=edsgvr&AN=edsgcl.3056800142&lang=es&site=eds-live&scope=site | 2. It is a reading-related technology but it is not a training (test, support…) |
| Bronuzzi, J., Mapelli, A., & Sallese, J. M. (2016). Analytical model for Transient Current Technique (TCT) signal prediction and analysis for thin interface characterization. Journal of Instrumentation, 11. https://doi.org/10.1088/1748-0221/11/12/C12080 | 5. Technology but it is not neither a training or reading-related |
| Brookes, G., Ng, V., Lim, B. H., Tan, W. P., & Lukito, N. (2011). The computerised-based Lucid Rapid Dyslexia Screening for the identification of children at risk of dyslexia: A Singapore study. Educational and Child Psychology. Dyslexia Association of Singapore, 73 Bukit Timah Road, Singapore 229832., Singapore. Retrieved from https://www.scopus.com/inward/record.uri?eid=2-s2.0-84871414852&partnerID=40&md5=93ac3d9b4fc5e1527bc97fe0a30e3df8 | 2. It is a reading-related technology but it is not a training (test, support…) |
| Brown, D. (2013). Types of words identified as unknown by L2 learners when reading. System, 41(4), 1043–1055. https://doi.org/10.1016/j.system.2013.10.013 | 9. Training oriented to a second-language learning |
| Brown, L. J. E., Adlam, T., Hwang, F., Khadra, H., Maclean, L. M., Rudd, B., … Astell, A. J. (2016). Computer-based tools for assessing micro-longitudinal patterns of cognitive function in older adults. Age, 38(4), 335–350. https://doi.org/10.1007/s11357-016-9934-x | 5. Technology but it is not neither a training or reading-related |
| Brunsdon, C. (2013). Computing with spatial trajectories , edited by Yu Zheng. International Journal of Geographical Information Science. Taylor & Francis. https://doi.org/10.1080/13658816.2012.741688 | 5. Technology but it is not neither a training or reading-related |
| Buerger, S., Kroehne, U., Koehler, C., & Goldhammer, F. (2019). What makes the difference? The impact of item properties on mode effects in reading assessments. STUDIES IN EDUCATIONAL EVALUATION, 62, 1–9. https://doi.org/10.1016/j.stueduc.2019.04.005 | 2. It is a reading-related technology but it is not a training (test, support…) |
| Bulger, M. E., Mayer, R. E., & Metzger, M. J. (2014). Knowledge and processes that predict proficiency in digital literacy. Reading and Writing, 27(9), 1567–1583. https://doi.org/10.1007/s11145-014-9507-2 | 2. It is a reading-related technology but it is not a training (test, support…) |
| Bunn,Tim, & Timothy Kenneth. (2006). The Effectiveness of Additional Interventions for Children with Literacy Difficulties in Years 3 & 4. Dyslexia, 14(3), 214–227. Retrieved from http://10.0.3.234/dys.372%0Ahttp://ezproxy.si.unav.es:2048/login?url=http://search.ebscohost.com/login.aspx?direct=true&AuthType=ip,url&db=pbh&AN=34095269&lang=es&site=eds-live&scope=site | 1.Non technological reading training |
| Burns, M. K., Kanive, R., & DeGrande, M. (2012). Effect of a Computer-Delivered Math Fact Intervention as a Supplemental Intervention for Math in Third and Fourth Grades. Remedial and Special Education, 33(3), 184–191. https://doi.org/10.1177/0741932510381652 | 3. Technological training that is not focus on improving reading |
| Burston, J. (2013). Mobile-Assisted Language Learning: a Selected Annotated Bibliography of Implementation Studies 1994–2012. Language Learning & Technology, 17(173), 157–225. https://doi.org/10.1080/0022027032000276961 | 11. Type of study excluded (review, non-primary references…) |
| Burton, R., Crabb, D. P., Smith, N. D., Glen, F. C., & Garway-Heath, D. F. (2012). Glaucoma and Reading. Optometry and Vision Science, 89(9), 1282–1287. https://doi.org/10.1097/OPX.0b013e3182686165 | 4. Reading-related but it is not neither a training or technological |
| Cabbage, K., Brinkley, S., Gray, S., Alt, M., Cowan, N., Green, S., Kuo, T., & Hogan, T. P. (2017). Assessing working memory in children: The comprehensive assessment battery for children-working memory (CABC-WM). Journal of Visualized Experiments, 2017(124). https://doi.org/10.3791/55121 | 10. Educational-related but it is not about reading |
| Cabbage, K., Brinkley, S., Gray, S., Alt, M., Cowan, N., Green, S., Kuo, T., & Hogan, T. P. (2017). Assessing working memory in children: The comprehensive assessment battery for children-working memory (CABC-WM). Journal of Visualized Experiments, 2017(124). https://doi.org/10.3791/55121 | 10. Educational-related but it is not about reading |
| Callegari, D. A., Jersak, L. C., & Da Costa, A. C. (2013). Technical trends and challenges in mobile health :A systematic review of recent available literature. In ICEIS 2013 - Proceedings of the 15th International Conference on Enterprise Information Systems (Vol. 2, pp. 519–525). Faculdade de Informática, FACIN, Pontifícia Universidade Católica do Rio Grande do Sul, Porto Alegre, RS, Brazil. Retrieved from https://www.scopus.com/inward/record.uri?eid=2-s2.0-84887776283&partnerID=40&md5=5e34d38528f8386ada98c88decc98389 | 5. Technology but it is not neither a training or reading-related |
| Cambra, A. B., & Murillo, A. C. (2011). Towards robust and efficient text sign reading from a mobile phone. In Proceedings of the IEEE International Conference on Computer Vision (pp. 64–71). DIIS, Instituto de Investigacíon en Ingenieŕia de Araǵon, University of Zaragoza, Spain. https://doi.org/10.1109/ICCVW.2011.6130223 | 2. It is a reading-related technology but it is not a training (test, support…) |
| Carlson, K. J. (2014). Incorporating iPad(RTM) Mobile Digital Devices within Repeated Reading Interventions for Students with Learning Disabilities. ProQuest Dissertations and Theses. ProQuest Information & Learning, US. Retrieved from http://search.proquest.com/docview/1562278994?accountid=13607 http://e-tidsskrifter.kb.dk/resolve??url_ver=Z39.88-2004&rft_val_fmt=info:ofi/fmt:kev:mtx:dissertation&genre=dissertations+%26+theses&sid=ProQ:ProQuest+Dissertations+%26+Theses+A%26I&atitle=&ti | 8. Training oriented to special population (other than dyslexia) |
| Caron, J., Light, J., Holyfield, C., & McNaughton, D. (2018). Effects of dynamic text in an AAC app on sight word reading for individuals with autism spectrum disorder. AAC: Augmentative and Alternative Communication, 34(2), 143–154. https://doi.org/10.1080/07434618.2018.1457715 | 2. It is a reading-related technology but it is not a training (test, support…) |
| Carson, K., Boustead, T., & Gillon, G. (2014). Predicting reading outcomes in the classroom using a computer-based phonological awareness screening and monitoring assessment (Com-PASMA). International Journal of Speech-Language Pathology, 16(6), 552–561. https://doi.org/10.3109/17549507.2013.855261 | 2. It is a reading-related technology but it is not a training (test, support…) |
| Carson, K., Boustead, T., & Gillon, G. (2015). Content validity to support the use of a computer-based phonological awareness screening and monitoring assessment (Com-PASMA) in the classroom. International Journal of Speech-Language Pathology, 17(5), 500–510. https://doi.org/10.3109/17549507.2015.1016107 | 2. It is a reading-related technology but it is not a training (test, support…) |
| Cartreine, J. A., Locke, S. E., Buckey, J. C., Sandoval, L., & Hegel, M. T. (2012). Electronic problem-solving treatment: Description and pilot study of an interactive media treatment for depression. Journal of Medical Internet Research, 14(5). https://doi.org/10.2196/resprot.1925 | 3. Technological training that is not focus on improving reading |
| Casey, J. (2019). Digital Shakespeare Is Neither Good Nor Bad, But Teaching Makes It So. Humanities (2076-0787), 8(2), 112. https://doi.org/10.3390/h8020112 | 5. Technology but it is not neither a training or reading-related |
| Castillo, A. (2008). The effect of computer-based authentic assignments on learners’ foreign language abilities and intrinsic motivation. Dissertation Abstracts International, A: The Humanities and Social Sciences. ProQuest Information & Learning, US. Retrieved from http://search.proquest.com/docview/85715979?accountid=14548%5Cnhttp://metadata.lib.hku.hk/hku?url_ver=Z39.88-2004&rft_val_fmt=info:ofi/fmt:kev:mtx:dissertation&genre=dissertations+&+theses&sid=ProQ:Linguistics+and+Language+Behavior+Abstracts+(LLBA)&ati | 9. Training oriented to a second-language learning |
| Castles, A., Coltheart, M., Larsen, L., Jones, P., Saunders, S., & McArthur, G. (2009). Assessing the basic components of reading: A revision of the castles and Coltheart test with new norms. Australian Journal of Learning Difficulties, 14(1), 67–88. https://doi.org/10.1080/19404150902783435 | 4. Reading-related but it is not neither a training or technological |
| Caulton, J. (2014). BARD Mobile App Helps Ensure That All May Read. Social Education, 78(2), 55. Retrieved from http://ezproxy.si.unav.es:2048/login?url=http://search.ebscohost.com/login.aspx?direct=true&AuthType=ip,url&db=asx&AN=95720212&lang=es&site=eds-live&scope=site | 2. It is a reading-related technology but it is not a training (test, support…) |
| Cazzell, S., Browarnik, B., Skinner, A., Skinner, C., Cihak, D., Ciancio, D., … Forbes, B. (2016). Extending Research on a Computer-Based Flashcard Reading Intervention to Postsecondary Students With Intellectual Disabilities. School Psychology Forum, 10(2), 191. Retrieved from http://ezproxy.si.unav.es:2048/login?url=http://search.ebscohost.com/login.aspx?direct=true&AuthType=ip,url&db=edo&AN=119790526&lang=es&site=eds-live&scope=site | 8. Training oriented to special population (other than dyslexia) |
| Cazzell, S., Skinner, C. H., Ciancio, D., Aspiranti, K., Watson, T., Taylor, K., … Skinner, A. (2016). Evaluating a Computer Flash-Card Sight-Word Recognition Intervention With Self-Determined Response Intervals in Elementary Students With Intellectual Disability. School Psychology Quarterly, 32(3), 367–378. https://doi.org/10.1037/spq0000172 | 8. Training oriented to special population (other than dyslexia) |
| Cazzell, S., Skinner, C. H., Ciancio, D., Aspiranti, K., Watson, T., Taylor, K., McCurdy, M., & Skinner, A. (2017). Evaluating a Computer Flash-Card Sight-Word Recognition Intervention With Self-Determined Response Intervals in Elementary Students With Intellectual Disability. SCHOOL PSYCHOLOGY QUARTERLY, 32(3), 367–378. https://doi.org/10.1037/spq0000172 | 2. It is a reading-related technology but it is not a training (test, support…) |
| Cazzell, S., Skinner, C. H., Taylor, K., McCurdy, M., Ciancio, D., Cihak, D., Skinner, A., & Moore, T. (2019). Comparing Computer-Based Sight-Word Interventions in Students with Intellectual Disability: Self-Determined Versus Fixed Response Intervals. Journal of Behavioral Education. https://doi.org/10.1007/s10864-019-09335-8 | 8. Training oriented to special population (other than dyslexia) |
| Celaschi, S., Castro, M. S., & da Cunha, S. P. (2017). Read it aloud to me. (S. C. & A. M., Eds.). Centro de Tecnologia da Informação Renato Archer, Campinas, Brazil: Springer Verlag. https://doi.org/10.1007/978-3-319-58703-5_19 | 2. It is a reading-related technology but it is not a training (test, support…) |
| Celaschi, S., Castro, M. S., & da Cunha, S. P. (2017). Read It Aloud to Me. In Antona, M and Stephanidis, C (Ed.), UNIVERSAL ACCESS IN HUMAN-COMPUTER INTERACTION: DESIGNING NOVEL INTERACTIONS, PT II (Vol. 10278, Issue II, pp. 260–268). https://doi.org/10.1007/978-3-319-58703-5_19 | 2. It is a reading-related technology but it is not a training (test, support…) |
| Celaschi, S., Castro, M. S., & da Cunha, S. P. (2017). Read It Aloud to Me. In Antona, M and Stephanidis, C (Ed.), UNIVERSAL ACCESS IN HUMAN-COMPUTER INTERACTION: DESIGNING NOVEL INTERACTIONS, PT II (Vol. 10278, Issue II, pp. 260–268). https://doi.org/10.1007/978-3-319-58703-5_19 | 2. It is a reading-related technology but it is not a training (test, support…) |
| Chamsaz, A. (2015). A Mobile Platform for Teaching Nonverbal Social Communication Skills to High-Functioning Autistic Children Using Discrete Trial Training. ProQuest Dissertations and Theses. ProQuest Information & Learning, US. Retrieved from http://proxy.bc.edu/login?url=http://search.proquest.com/docview/1754367950?accountid=9673%5Cnhttp://bc-primo.hosted.exlibrisgroup.com/openurl/BCL/services_page??url_ver=Z39.88-2004&rft_val_fmt=info:ofi/fmt:kev:mtx:dissertation&genre=dissertations+%26+the | 3. Technological training that is not focus on improving reading |
| Chang, Y., & Liu, F. (2016). Network traffic and user behavior analysis of mobile reading applications. 2016 Sixth International Conference on Information Science & Technology (ICIST), 142. Retrieved from http://ezproxy.si.unav.es:2048/login?url=http://search.ebscohost.com/login.aspx?direct=true&AuthType=ip,url&db=edb&AN=116452360&lang=es&site=eds-live&scope=site | 2. It is a reading-related technology but it is not a training (test, support…) |
| ChanLin, L.-J. (2015). Tablet Reading Service for College Students. Procedia - Social and Behavioral Sciences, 197(7th World Conference on Educational Sciences), 231–235. https://doi.org/10.1016/j.sbspro.2015.07.129 | 2. It is a reading-related technology but it is not a training (test, support…) |
| ChanLin, L.-J. L.-J. (2018). Bridging children’s reading with an augmented reality story library. LIBRI, 68(3), 219–229. https://doi.org/10.1515/libri-2018-0017 | 5. Technology but it is not neither a training or reading-related |
| Chao, P., & Chen, G. (2009). Interacting with Computers Augmenting paper-based learning with mobile phones. Interacting with Computers, 21(3), 173–185. https://doi.org/10.1016/j.intcom.2009.01.001 | 5. Technology but it is not neither a training or reading-related |
| Chase, P. N. (2014). Gathering Evidence for Distance Education. Acta de Investigación Psicológica, 4(3), 1657–1672. https://doi.org/10.1016/S2007-4719(14)70972-X | 10. Educational-related but it is not about reading |
| Chauhan, S. (2017). A meta-analysis of the impact of technology on learning effectiveness of elementary students. Computers and Education, 105, 14–30. https://doi.org/10.1016/j.compedu.2016.11.005 | 11. Type of study excluded (review, non-primary references…) |
| Chavez, R. A., & Gimino, A. (2010). Innovation and integration: Case studies of effective teacher practices in the use of handheld computers. ProQuest LLC. ProQuest LLC. Retrieved from http://proxy2.hec.ca/login?url=http://search.proquest.com/docview/518584570?accountid=11357%5Cnhttp://gutenberg.hec.ca:3210/sfxlcl3?url_ver=Z39.88-2004&rft_val_fmt=info:ofi/fmt:kev:mtx:dissertation&genre=dissertations+%26+theses&sid=ProQ:ProQuest+Disserta | 5. Technology but it is not neither a training or reading-related |
| Che Pee, N., Leong, P. H., Othman, M. A., Sulaiman, H. A., Othman, M. F. I., & Rahim, Y. A. (2016). Dycscreen- cross-platform dyslexia screening test for Malaysian children through hybrid applications. (S. H.A., O. M.A., O. M.F.I., R. Y.A., & P. N.C., Eds.), Lecture Notes in Electrical Engineering. Communication, Multimedia, Networks and Systems Research Group, Universiti Teknikal Malaysia Melaka (UTeM), Durian Tunggal, Melaka, Malaysia: Springer Verlag. https://doi.org/10.1007/978-3-319-24584-3_92 | 2. It is a reading-related technology but it is not a training (test, support…) |
| Chee, K. N., Yahaya, N., & Ibrahim, N. H. (2017). Effectiveness of mobile learning application in improving reading skills in Chinese language and towards post-attitudes. International Journal of Mobile Learning and Organisation, 11(3), 210–225. https://doi.org/10.1504/IJMLO.2017.085347 | 12. Other: No access granted, same data… |
| Chen, C. M., & Lin, Y. J. (2016). Effects of different text display types on reading comprehension, sustained attention and cognitive load in mobile reading contexts. Interactive Learning Environments, 24(3), 553–571. https://doi.org/10.1080/10494820.2014.891526 | 2. It is a reading-related technology but it is not a training (test, support…) |
| Chen, X., Breslow, L., & DeBoer, J. (2018). Analyzing productive learning behaviors for students using immediate corrective feedback in a blended learning environment. Computers and Education, 117, 59–74. https://doi.org/10.1016/j.compedu.2017.09.013 | 3. Technological training that is not focus on improving reading |
| Cheng, L., & Beal, C. R. (2020). Effects of student-generated drawing and imagination on science text reading in a computer-based learning environment. ETR&D-EDUCATIONAL TECHNOLOGY RESEARCH AND DEVELOPMENT, 68(1), 225–247. https://doi.org/10.1007/s11423-019-09684-1 | 10. Educational-related but it is not about reading |
| Cheng, L., & Beal, C. R. (2020). Effects of student-generated drawing and imagination on science text reading in a computer-based learning environment. ETR&D-EDUCATIONAL TECHNOLOGY RESEARCH AND DEVELOPMENT, 68(1), 225–247. https://doi.org/10.1007/s11423-019-09684-1 | 10. Educational-related but it is not about reading |
| Chiang, H.-H. (2020). Kahoot! In an EFL Reading Class. Journal of Language Teaching & Research, 11(1), 33. https://ezproxy.unav.es/login?url=https://search.ebscohost.com/login.aspx?direct=true&AuthType=ip,url&db=edb&AN=141063708&lang=es&site=eds-live&scope=site | 9. Training oriented to a second-language learning |
| Chiang, H.-Y. A., & Jacobs, K. (2010). Perceptions of a computer-based instruction system in special education: high school teachers and students views. Work, 37(4), 349–359. https://doi.org/10.3233/WOR-2010-1089 | 10. Educational-related but it is not about reading |
| Chiu, P.-S. (2018). A Cloud Bookcase System for Mobile Library. Proceedings - 2018 7th International Congress on Advanced Applied Informatics, IIAI-AAI 2018, 932–933. https://doi.org/10.1109/IIAI-AAI.2018.00188 | 5. Technology but it is not neither a training or reading-related |
| Chu, Y.-L., Hsieh, H.-E., Lin, W.-H., Chen, H.-J., & Chou, C.-H. (2017). Chinese FingerReader: A wearable device to explore Chinese printed text. ACM SIGGRAPH 2017 Posters, SIGGRAPH 2017. https://doi.org/10.1145/3102163.3102206 | 2. It is a reading-related technology but it is not a training (test, support…) |
| Cidrim, L., Braga, P. H. M. M., & Madeiro, F. (2018). Desembaralhando: a mobile application for intervention in the problem of dyslexic children mirror writing. Revista CEFAC: Atualizacao Cientifica Em Fonoaudiologia e Educacao VO - 20, 1, 13. https://doi.org/10.1590/1982-0216201820111917 | 3. Technological training that is not focus on improving reading |
| Ciuffreda, K. J., Yadav, N. K., Thiagarajan, P., & Ludlam, D. P. (2017). A novel computer oculomotor rehabilitation (COR) program for mild traumatic brain injury (mTBI). Brain Sciences, 7(8). https://doi.org/10.3390/brainsci7080099 | 8. Training oriented to special population (other than dyslexia) |
| Ciullo, S. P., & Reutebuch, C. (2013). Computer-based graphic organizers for students with LD: A systematic review of literature. Learning Disabilities Research and Practice, 28(4), 196–210. https://doi.org/10.1111/ldrp.12017 | 11. Type of study excluded (review, non-primary references…) |
| Ciullo, S., Falcomata, T. S., Pfannenstiel, K., & Billingsley, G. (2015). Improving Learning With Science and Social Studies Text Using Computer-Based Concept Maps for Students With Disabilities. Behavior Modification, 39(1), 117–135. https://doi.org/10.1177/0145445514552890 | 2. It is a reading-related technology but it is not a training (test, support…) |
| Clearinghouse, W. W. (2013). What Works Clearinghouse TM Read Naturally ®. What Works Clearinghouse. What Works Clearinghouse. Retrieved from http://ezproxy.si.unav.es:2048/login?url=http://search.ebscohost.com/login.aspx?direct=true&AuthType=ip,url&db=eric&AN=ED544197&lang=es&site=eds-live&scope=site | 11. Type of study excluded (review, non-primary references…) |
| Colejo Blanco, V. (2019). Development of a videogame to improve the reading and writing abilities of children with dyslexia in a playful way. Universitat Jaume I. https://ezproxy.unav.es/login?url=https://search.ebscohost.com/login.aspx?direct=true&AuthType=ip,url&db=edsbas&AN=edsbas.7ADFCF63&lang=es&site=eds-live&scope=site | 2. It is a reading-related technology but it is not a training (test, support…) |
| Conradi, J. (2017). Influence of letter size on word reading performance during walking. PROCEEDINGS OF THE 19TH INTERNATIONAL CONFERENCE ON HUMAN-COMPUTER INTERACTION WITH MOBILE DEVICES AND SERVICES (MOBILEHCI `17). https://doi.org/10.1145/3098279.3098554 | 4. Reading-related but it is not neither a training or technological |
| Conradi, J. (2017). Influence of letter size on word reading performance during walking. PROCEEDINGS OF THE 19TH INTERNATIONAL CONFERENCE ON HUMAN-COMPUTER INTERACTION WITH MOBILE DEVICES AND SERVICES (MOBILEHCI `17). https://doi.org/10.1145/3098279.3098554 | 2. It is a reading-related technology but it is not a training (test, support…) |
| Core, M., & Prunty, A. (2012). Implementing a computer-based reading intervention in a special school setting for adolescents with mild general learning disabilities. In Special and Inclusive Education: A Research Perspective (pp. 261–274). Peter Lang Publishing Group. Retrieved from https://www.scopus.com/inward/record.uri?eid=2-s2.0-84924617955&partnerID=40&md5=4f051d85356307be0611339dc288b0a7 | 8. Training oriented to special population (other than dyslexia) |
| Correa  Ik; Inoue, Ty; Kitamura, Rk; Ribeir jr, _; Tahira, A;, A. F. (2013). Development of an interactive book with Augmented Reality for mobile learning. None;  CO  - Agera#1; Aisti#1; Animation#1; Book#1; Camera#1; Complement#1; Education#3; Excite#1; Fun#1; Geometry#1; Geometry Book#1; Interactive Book#1; Interactive Element#1; Leap#2; Marker#1; Mobile Device#1; Motivate#1; Page#1; Reality#6; Software#3;, 1, 150. Retrieved from http://ezproxy.si.unav.es:2048/login?url=http://search.ebscohost.com/login.aspx?direct=true&AuthType=ip,url&db=edb&AN=109114480&lang=es&site=eds-live&scope=site | 2. It is a reading-related technology but it is not a training (test, support…) |
| Corrêa, A. G. D. (2013). Interactive Books in Augmented Reality for Mobile Devices. In Technology Platform Innovations and Forthcoming Trends in Ubiquitous Learning (Vol. i, pp. 1–18). Universidade Presbiteriana Mackenzie, Brazil: IGI Global. https://doi.org/10.4018/978-1-4666-4542-4.ch001 | 2. It is a reading-related technology but it is not a training (test, support…) |
| Costa, L. C. P., Correa, A. G. D., Dalmon, D. L., Zuffo, M. K., & Lopes, R. D. (2015). Accessible educational digital book on tablets for people with visual impairment. IEEE Transactions on Consumer Electronics, 61(3), 271–278. https://doi.org/10.1109/TCE.2015.7298085 | 8. Training oriented to special population (other than dyslexia) |
| Costa, L. C. P., Correa, A. G. D., Leite, D., Zuffo, M. K., & Lopes, R. D. (2015). Educadaisy: Accessible digital didactic book on tablets for people with visual impairment. In 2015 IEEE International Conference on Consumer Electronics, ICCE 2015 (pp. 78–79). University of Sao Paulo, São Paulo, SP, Brazil; LSI-TEC, São Paulo, SP, Brazil: Institute of Electrical and Electronics Engineers Inc. https://doi.org/10.1109/ICCE.2015.7066326 | 8. Training oriented to special population (other than dyslexia) |
| Craighead, J. (2008). Distributed, game-based, intelligent tutoring systems - The next step in computer based training? In 2008 International Symposium on Collaborative Technologies and Systems, CTS’08 (pp. 247–256). Department of Computer Science, University of South Florida, Tampa, FL 33620, United States. https://doi.org/10.1109/CTS.2008.4543938 | 3. Technological training that is not focus on improving reading |
| Crawford, L., Higgins, K. N., Huscroft-D’Angelo, J. N., & Hall, L. (2016). Students’ use of electronic support tools in mathematics. Etr&D-Educational Technology Research and Development, 64(6), 1163–1182. https://doi.org/10.1007/s11423-016-9452-7 | 3. Technological training that is not focus on improving reading |
| Crichton, S., Pegler, K., & White, D. (2012). Personal Devices in Public Settings : Lessons Learned From an iPod Touch / iPad Project Learning Innovations , Calgary Board of Education , Calgary , Canada. Education, 10(1), 23–31. Retrieved from https://www.scopus.com/inward/record.uri?eid=2-s2.0-84904763885&partnerID=40&md5=cc41fa564e64a8533b648187845b7779 | 10. Educational-related but it is not about reading |
| Cuevas Martínez, M., & Lletres, U. A. de B. F. de F. i. (2019). Reading Academic English : a case study. StratApp - The Art of Learning. https://ezproxy.unav.es/login?url=https://search.ebscohost.com/login.aspx?direct=true&AuthType=ip,url&db=edsbas&AN=edsbas.1AA404C4&lang=es&site=eds-live&scope=site | 10. Educational-related but it is not about reading |
| Dafhe Ifigenia, P. R., Jaime, M. A., Julien, B., & Julio Cesar, P. G. (2018). Integration of Gamification to Assist Literacy in Children with Special Educational Needs. PROCEEDINGS OF 2018 IEEE GLOBAL ENGINEERING EDUCATION CONFERENCE (EDUCON) - EMERGING TRENDS AND CHALLENGES OF ENGINEERING EDUCATION, 1949–1956. | 8. Training oriented to special population (other than dyslexia) |
| Dafhe Ifigenia, P. R., Jaime, M. A., Julien, B., & Julio Cesar, P. G. (2018). Integration of Gamification to Assist Literacy in Children with Special Educational Needs. PROCEEDINGS OF 2018 IEEE GLOBAL ENGINEERING EDUCATION CONFERENCE (EDUCON) - EMERGING TRENDS AND CHALLENGES OF ENGINEERING EDUCATION, 1949–1956. | 8. Training oriented to special population (other than dyslexia) |
| Dasila, R. S., Trivedi, M., Soni, S., Senthil, M., & Narendran, M. (2017). Real Time Environment Perception For Visually Impaired. 2017 IEEE TECHNOLOGICAL INNOVATIONS IN ICT FOR AGRICULTURE AND RURAL DEVELOPMENT (TIAR), 168–172. | 8. Training oriented to special population (other than dyslexia) |
| Daud, S. M., & Abas, H. (2014). “Dyslexia baca” mobile app - The learning ecosystem for dyslexic children. In Proceedings - 2013 International Conference on Advanced Computer Science Applications and Technologies, ACSAT 2013 (pp. 412–416). https://doi.org/10.1109/ACSAT.2013.87 | 2. It is a reading-related technology but it is not a training (test, support…) |
| De Barros, M. A., Andrade, V., Moura, J. A. B., Borgmann, L., Terton, U., Vieira, F., Da Costa, G. C. A., Araújo, R. L., Arruda, A. O., Naviner, S., & Silva, J. (2018). ReadAct: Alternate reality, serious games for reading-acting to engage population and schools on social challenges. In Z. S. U. J. Reilly R. McLaren B.M. (Ed.), CSEDU 2018 - Proceedings of the 10th International Conference on Computer Supported Education (Vol. 2, pp. 238–245). SciTePress. https://www.scopus.com/inward/record.uri?eid=2-s2.0-85047731980&partnerID=40&md5=3e52b9480fe767a2f4dcbeabbb995ede | 3. Technological training that is not focus on improving reading |
| De Cara, B., & Plaza, M. (2010). Computer-based reading instructional programs: What does research tell us? ANAE - Approche Neuropsychologique des Apprentissages chez l’Enfant, 22(107–108), 184–190. Retrieved from https://www.scopus.com/inward/record.uri?eid=2-s2.0-77957704238&partnerID=40&md5=ee7e3b7126a891fe627e37f3216d4aa9 | 11. Type of study excluded (review, non-primary references…) |
| de Castell, S., & Jenson, J. (2018). �“We Should Play More Games!” Learning With Narrative Videogames. In C. M. (Ed.), Proceedings of the European Conference on Games-based Learning (Vols. 2018-Octob, pp. 45–53). Dechema e.V. https://www.scopus.com/inward/record.uri?eid=2-s2.0-85058942182&partnerID=40&md5=6cb81bd5fc058a5fed6622c63bb039d8 | 7. Technological reading training that do not fulfill methods requirements |
| de Jesus, I. P., Santos, R. do R., & de Sousa, A. C. (2017). The mobile library and the reading habits: a case study of BiblioSESC in Salvador’s neighborhood. ENCONTROS BIBLI-REVISTA ELETRONICA DE BIBLIOTECONOMIA E CIENCIA DA INFORMACAO, 22(50), 102–113. https://doi.org/10.5007/1518-2924.2017v22n50p102 | 2. It is a reading-related technology but it is not a training (test, support…) |
| De Kock, R., & Futcher, L. A. (2016). Mobile device usage in higher education institutions in South Africa. 2016 Information Security for South Africa (ISSA), 27–34. https://doi.org/10.1109/ISSA.2016.7802925 | 10. Educational-related but it is not about reading |
| de Moll, F. (2019). The organization of children’s daily lives as a determinant of academic per formance: how are reading and playing videogames related to children’s success in primar y school? [Die alltagsgestaltung von kindern als determinante des schulerfolgs: Wie hängen. Zeitschrift Fur Soziologie Der Erziehung Und Sozialisation, 39(3), 265–280. https://www.scopus.com/inward/record.uri?eid=2-s2.0-85077075982&partnerID=40&md5=6ad6f8ed06c42d0ccec551de27b94407 | 10. Educational-related but it is not about reading |
| de Moll, F. (2019). The Organization of Children’s Daily Lives as a Determinant of Academic Performance: How are Reading and Playing Videogames Related to Children’s Success in Primary School? ZEITSCHRIFT FUR SOZIOLOGIE DER ERZIEHUNG UND SOZIALISATION, 39(3), 265–280. | 10. Educational-related but it is not about reading |
| Degryse, J., De Lepeleire, J., Southgate, L., Vernooij-Dassen, M., Gay, B., & Heyrman, J. (2009). An evaluation of a computer based education program for the diagnosis and management of dementia in primary care. An international study of the transcultural adaptations necessary for European dissemination. Medical Teacher, 31(5), 397–402. https://doi.org/10.1080/01421590802331438 | 8. Training oriented to special population (other than dyslexia) |
| Delavarian, M., Afrooz, A., Towhidkhah, F., & Tabatabaei, K. R. (2017). Designing a computerized neuro-cognitive program for early diagnosing children at risk for dyslexia. Iranian Rehabilitation Journal, 15(2), 103–110. https://doi.org/10.18869/nrip.irj.15.2.103 | 2. It is a reading-related technology but it is not a training (test, support…) |
| Dell, N., & Borriello, G. (2013). Mobile tools for point-of-care diagnostics in the developing world. In Proceedings of the 3rd ACM Symposium on Computing for Development - ACM DEV ’13 (p. 1). Computer Science and Engineering, University of Washington, United States. https://doi.org/10.1145/2442882.2442894 | 5. Technology but it is not neither a training or reading-related |
| Demmans Epp, C., Munteanu, C., Axtell, B., Ravinthiran, K., Aly, Y., & Mansimov, E. (2017). Finger Tracking: Facilitating non-commercial content production for mobile E-reading applications. Proceedings of the 19th International Conference on Human-Computer Interaction with Mobile Devices and Services, MobileHCI 2017. https://doi.org/10.1145/3098279.3098556 | 5. Technology but it is not neither a training or reading-related |
| Des Roches, C. A., & Kiran, S. (2017). Technology-Based Rehabilitation to Improve Communication after Acquired Brain Injury. FRONTIERS IN NEUROSCIENCE, 11. https://doi.org/10.3389/fnins.2017.00382 | 8. Training oriented to special population (other than dyslexia) |
| Deshpande, A., Desrochers, A., Ksoll, C., & Shonchoy, A. S. (2017). The Impact of a Computer-based Adult Literacy Program on Literacy and Numeracy: Evidence from India. WORLD DEVELOPMENT, 96, 451–473. https://doi.org/10.1016/j.worlddev.2017.03.029 | 12. Other: No access granted, same data… |
| Devena, S. (2013). Relationship of Oral Reading Fluency Probes on Students’ Reading Achievement Test Scores. ProQuest Dissertations and Theses. ProQuest LLC, US. Retrieved from http://proxy.seattleu.edu:2048/login?url=http://search.proquest.com/docview/1468723312?accountid=28598%5Cnhttp://primo.seattleu.edu/openurl/SEAU/seau_services_page?genre=unknown&issn=9781303563188&jtitle=&atitle=&aulast=Devena&date=2013&spage=&issue=&volu | 2. It is a reading-related technology but it is not a training (test, support…) |
| Di Tore, S., Zollo, I., Maffei, S., & Sibilio, M. (2016). Design and Development of an Inclusive Learning Videogames Aimed to Promote the Development of Reading and Writing Skills. International Journal of Digital Literacy & Digital Competence, 7(2), 34. Retrieved from http://ezproxy.si.unav.es:2048/login?url=http://search.ebscohost.com/login.aspx?direct=true&AuthType=ip,url&db=edb&AN=117080066&lang=es&site=eds-live&scope=site | 11. Type of study excluded (review, non-primary references…) |
| Diamanti, V., Mouzaki, A., Ralli, A., Antoniou, F., Papaioannou, S., & Protopapas, A. (2017). Preschool Phonological and Morphological Awareness As Longitudinal Predictors of Early Reading and Spelling Development in Greek. FRONTIERS IN PSYCHOLOGY, 8. https://doi.org/10.3389/fpsyg.2017.02039 | 2. It is a reading-related technology but it is not a training (test, support…) |
| Diamanti, V., Mouzaki, A., Ralli, A., Antoniou, F., Papaioannou, S., & Protopapas, A. (2017). Preschool Phonological and Morphological Awareness As Longitudinal Predictors of Early Reading and Spelling Development in Greek. FRONTIERS IN PSYCHOLOGY, 8. https://doi.org/10.3389/fpsyg.2017.02039 | 4. Reading-related but it is not neither a training or technological |
| Didur, J., & Fan, L.-T. (2018). Between Landscape and the Screen: Locative Media,Transitive Reading, and Environmental Storytelling. https://ezproxy.unav.es/login?url=https://search.ebscohost.com/login.aspx?direct=true&AuthType=ip,url&db=edsbas&AN=edsbas.77112050&lang=es&site=eds-live&scope=site | 5. Technology but it is not neither a training or reading-related |
| Dingler, T., Tag, B., Schmidt, A., & Lehrer, S. (2018). Reading scheduler: Proactive recommendations to help users cope with their daily reading volume. In A. F. Abdennadher S. (Ed.), ACM International Conference Proceeding Series (pp. 239–244). Association for Computing Machinery. https://doi.org/10.1145/3282894.3282917 | 2. It is a reading-related technology but it is not a training (test, support…) |
| Dingler, T., Tag, B., Schmidt, A., & Lehrer, S. (2018). Reading scheduler: Proactive recommendations to help users cope with their daily reading volume. In A. F. Abdennadher S. (Ed.), ACM International Conference Proceeding Series (pp. 239–244). Association for Computing Machinery. https://doi.org/10.1145/3282894.3282917 | 2. It is a reading-related technology but it is not a training (test, support…) |
| Doerfler, T., Golke, S., & Artelt, C. (2017). Evaluating Prerequisites for the Development of a Dynamic Test of Reading Competence: Feedback Effects on Reading Comprehension in Children. In Leutner, D and Fleischer, J and Grunkorn, J and Klieme, E (Ed.), COMPETENCE ASSESSMENT IN EDUCATION: RESEARCH, MODELS AND INSTRUMENTS (pp. 487–503). https://doi.org/10.1007/978-3-319-50030-0_28 | 2. It is a reading-related technology but it is not a training (test, support…) |
| Domack, A. M. (2009). “Let’s read a book, Mommy”: How gender, age, and socioeconomic status affect naturalistic conversations about literacy. Dissertation Abstracts International Section A: Humanities and Social Sciences. ProQuest Information & Learning, US. Retrieved from http://search.ebscohost.com/login.aspx?direct=true&db=psyh&AN=2009-99171-076&site=ehost-live | 2. It is a reading-related technology but it is not a training (test, support…) |
| Donahue, S. (2008). Online Learning Comes of Age. Language Magazine, 8(3), 52. Retrieved from http://ezproxy.si.unav.es:2048/login?url=http://search.ebscohost.com/login.aspx?direct=true&AuthType=ip,url&db=edb&AN=35233386&lang=es&site=eds-live&scope=site | 11. Type of study excluded (review, non-primary references…) |
| Dong, C., & Si, Z. (2018). The Research and Application of Augmented Reality in 3D Interactive Books for Children. In Zhao, P and Ouyang, Y and Xu, M and Yang, L and Ren, Y (Ed.), APPLIED SCIENCES IN GRAPHIC COMMUNICATION AND PACKAGING (Vol. 477, pp. 293–299). https://doi.org/10.1007/978-981-10-7629-9_35 | 2. It is a reading-related technology but it is not a training (test, support…) |
| Dong, C., & Si, Z. (2018). The research and application of augmented reality in 3D interactive books for children. Lecture Notes in Electrical Engineering, 477, 293–299. https://doi.org/10.1007/978-981-10-7629-9_35 | 2. It is a reading-related technology but it is not a training (test, support…) |
| Dörfler, T., Golke, S., & Artelt, C. (2017). Evaluating prerequisites for the development of a dynamic test of reading competence: Feedback effects on reading comprehension in children. In D. Leutner, J. Fleischer, J. Grünkorn, E. Klieme, D. Leutner  (Ed), J. Fleischer  (Ed), … E. Klieme  (Ed) (Eds.), Competence assessment in education: Research, models and instruments. (pp. 487–503). Dörfler, Tobias, University of Education, Heidelberg, Heidelberg, Germany: Springer International Publishing. https://doi.org/10.1007/978-3-319-50030-0_28 | 2. It is a reading-related technology but it is not a training (test, support…) |
| Dorris, S. (2014). The Effects of Computer-Assisted Instruction on the Reading Achievement of Elementary School Students. ProQuest Dissertations and Theses. ProQuest Information & Learning, US. Retrieved from http://flagship.luc.edu/login?url=http://search.proquest.com/docview/1561546772?accountid=12163%5Cnhttp://loyola-primo.hosted.exlibrisgroup.com/openurl/01LUC/01LUC_SERVICES?genre=dissertations+%26+theses&issn=&title=The+Effects+of+Computer-Assisted+Instru | 12. Other: No access granted, same data… |
| Dotan, S., & Katzir, T. (2018). Mind the gap: Increased inter-letter spacing as a means of improving reading performance. JOURNAL OF EXPERIMENTAL CHILD PSYCHOLOGY, 174, 13–28. https://doi.org/10.1016/j.jecp.2018.04.010 | 2. It is a reading-related technology but it is not a training (test, support…) |
| Drapkin, Z. A., Lindgren, K. A., Lopez, M. J., & Stabio, M. E. (2015). Development and assessment of a new 3D neuroanatomy teaching tool for MRI training. Anatomical Sciences Education, 8(6), 502–509. https://doi.org/10.1002/ase.1509 | 10. Educational-related but it is not about reading |
| Druin, A., Bederson, B. B., & Quinn, A. (2009). Designing intergenerational mobile storytelling. In Proceedings of the 8th International Conference on Interaction Design and Children - IDC ’09 (p. 325). Human-Computer Interaction Lab (HCIL), College Park, University of Maryland, Maryland 20742, United States. https://doi.org/10.1145/1551788.1551875 | 2. It is a reading-related technology but it is not a training (test, support…) |
| Duffy, F. F., Fochtmann, L. J., Clarke, D. E., Barber, K., Hong, S. H., Yager, J., … Plovnick, R. M. (2016). Psychiatrists’ Comfort Using Computers and Other Electronic Devices in Clinical Practice. Psychiatric Quarterly, 87(3), 571–584. https://doi.org/10.1007/s11126-015-9410-2 | 5. Technology but it is not neither a training or reading-related |
| Dunkerly-Bean, J. M., & Crompton, H. (2015). The Role of Mobile Learning in Promoting Literacy and Human Rights for Women and Girls. In Handbook of Research on the Societal Impact of Digital Media (Vol. 4, pp. 581–608). Old Dominion University, United States: IGI Global. https://doi.org/10.4018/978-1-4666-8310-5.ch023 | 10. Educational-related but it is not about reading |
| Dürebrandt, J. (2015). Segmentation and Beautification of Handwriting using Mobile Devices. Retrieved from http://www.diva-portal.org/smash/record.jsf?pid=diva2:808277 | 2. It is a reading-related technology but it is not a training (test, support…) |
| Duret, C., & Pons, C.-M. (2016). Contemporary Research on Intertextuality in Video Games. Contemporary Research on Intertextuality in Video Games. Université de SherbrookeQC, Canada: IGI Global. https://doi.org/10.4018/978-1-5225-0477-1 | 5. Technology but it is not neither a training or reading-related |
| Duursma, E., Meijer, A., & de Bot, K. (2017). The Impact of Home Literacy and Family Factors on Screen Media Use Among Dutch Preteens. Journal of Child and Family Studies, 26(2), 612–622. https://doi.org/10.1007/s10826-016-0584-5 | 2. It is a reading-related technology but it is not a training (test, support…) |
| Dwi, C. R. A., Nur, H. A., Prasetya, W. A., Cahya, R. A. D., Handayani, A. N., Wibawa, A. P., Dwi, C. R. A., Nur, H. A., & Prasetya, W. A. (2018). Mobile Braille Touch Application for Visually Impaired People using Double Diamond Approach. MATEC Web of Conferences VO  - 197, 197, 15007. https://doi.org/10.1051/matecconf/201819715007 | 8. Training oriented to special population (other than dyslexia) |
| Dymora, P., & Niemiec, K. (2019). Gamification as a Supportive Tool for School Children with Dyslexia. INFORMATICS-BASEL, 6(4). https://doi.org/10.3390/informatics6040048 | 3. Technological training that is not focus on improving reading |
| Earman Stetter, M., & Tejero Hughes, M. (2010). Computer assisted instruction to enhance the reading comprehension of struggling readers: A review of the literature. Journal of Special Education Technology, 25(4), 1–16. https://doi.org/10.1177/016264341002500401 | 11. Type of study excluded (review, non-primary references…) |
| Ecalle, J. (2010). Reading assessment and related competencies . Revue Francaise de Linguistique Appliquee, 15(1), 105–120. Retrieved from http://www.scopus.com/inward/record.url?eid=2-s2.0-77955399013&partnerID=40&md5=0e60feddc56333c140a10d3e506aec08 | 4. Reading-related but it is not neither a training or technological |
| Ecalle, J., Vidalenc, J.-L., Ballet, C., & Magnan, A. (2019). From Fundamental Research to the Design of a Software Solution to Help Poor Readers. Journal of Educational Computing Research. https://doi.org/10.1177/0735633119845447 | 7. Technological reading training that do not fulfill methods requirements |
| Education, U. S. D. of. (2010). What Works Clearinghouse: Fast ForWord. Language, (August), 57. | 11. Type of study excluded (review, non-primary references…) |
| Einum, E. (2015). ASSESSING AND TRAINING LANGUAGE SKILLS WITH MOBILE TECHNOLOGIES AND METHODS. In Chova, LG and Martinez, AL and Torres, IC (Ed.), INTED2015: 9TH INTERNATIONAL TECHNOLOGY, EDUCATION AND DEVELOPMENT CONFERENCE (pp. 5093–5098). | 7. Technological reading training that do not fulfill methods requirements |
| Einum, E. (2015). New Conclusions in Assessment, Revision and Consolidation of English Second Language Knowledge with Applied Student Response Software. In GomezChova, L and LopezMartinez, A and CandelTorres, I (Ed.), Edulearn15: 7th International Conference on Education and New Learning Technologies (pp. 4614–4622). | 9. Training oriented to a second-language learning |
| Ekhsan, H. M., Ahmad, S. Z., Halim, S. A., Hamid, J. N., & Mansor, N. H. (2012). The implementation of interactive multimedia in early screening of dyslexia. ICIMTR 2012 - 2012 International Conference on Innovation, Management and Technology Research, 566–569. https://doi.org/10.1109/ICIMTR.2012.6236459 | 2. It is a reading-related technology but it is not a training (test, support…) |
| El Baki, M. A., Omar, K., Bin Nor Aripin, K. N., Abdul Rahman, T. S., Ithnin, M., Syaiful, A., … Muhammad, T. (2015). A Systematic Review of Computer-Based Remedial Programs for Primary Schoolchildren Diagnosed With Dyslexia: Results From Medline. Pediatrics, 135(Supplement), S6–S6. https://doi.org/10.1542/peds.2014-3330I | 11. Type of study excluded (review, non-primary references…) |
| El Ebyary, K., & Windeatt, S. (2019). Eye tracking analysis of EAP Students’ regions of interest in computer-based feedback on grammar, usage, mechanics, style and organization and development. SYSTEM, 83(SI), 36–49. https://doi.org/10.1016/j.system.2019.03.007 | 4. Reading-related but it is not neither a training or technological |
| EL Ghouati, A. (2017). Examining the Relationship between E-Learning Styles and Achievement in English among Moroccan University Students. ARAB WORLD ENGLISH JOURNAL, 8(2), 323–332. https://doi.org/10.24093/awej/vol8no2.23 | 9. Training oriented to a second-language learning |
| Elaalyani, I., Erradi, M., Mühling, M., & Freisleben, B. (2017). Current trends in text-spotting. In H. M. Y. Kobbane A. Ibrahimi K. (Ed.), Proceedings - 2017 International Conference on Wireless Networks and Mobile Communications, WINCOM 2017. Institute of Electrical and Electronics Engineers Inc. https://doi.org/10.1109/WINCOM.2017.8238215 | 2. It is a reading-related technology but it is not a training (test, support…) |
| Elmer, S., & Jäncke, L. (2014). Intracerebral Functional Connectivity-Guided Neurofeedback as a Putative Rehabilitative Intervention for Ameliorating Auditory-Related Dysfunctions. Frontiers in Psychology, 5(OCT). https://doi.org/10.3389/fpsyg.2014.01227 | 4. Reading-related but it is not neither a training or technological |
| Emily, H. (2015). Mapping a path to understanding: Supporting the comprehension of narrative text for students with learning disabilities and typically developing peers. ProQuest Dissertations and Theses. ProQuest Information & Learning, US. Retrieved from http://search.proquest.com/docview/1700219272?accountid=13607 http://e-tidsskrifter.kb.dk/resolve??url_ver=Z39.88-2004&rft_val_fmt=info:ofi/fmt:kev:mtx:dissertation&genre=dissertations+%26+theses&sid=ProQ:ProQuest+Dissertations+%26+Theses+A%26I&atitle=&ti | 8. Training oriented to special population (other than dyslexia) |
| English, J., & Richardson, L. (2017). SimplyE - More people discovering more from the library. D-Lib Magazine, 23(5–6). https://doi.org/10.1045/may2017-english | 2. It is a reading-related technology but it is not a training (test, support…) |
| Enni, F. (2011). The Leaf project : a first application. KTH, Kommunikationssystem, CoS. Retrieved from http://ezproxy.si.unav.es:2048/login?url=http://search.ebscohost.com/login.aspx?direct=true&AuthType=ip,url&db=edsair&AN=edsair.od.......260..ad637839751c3d570f3c4ba7182067e4&lang=es&site=eds-live&scope=site | 2. It is a reading-related technology but it is not a training (test, support…) |
| Eno, L. P. (2011). Comparing the Reading Performance of High-Achieving Adolescents: Computer-Based Testing versus Paper/Pencil. ProQuest LLC. ProQuest Information & Learning, US. Retrieved from http://search.ebscohost.com/login.aspx?direct=true&db=eric&AN=ED549076&site=ehost-live%5Cnhttp://gateway.proquest.com/openurl?url_ver=Z39.88-2004&rft_val_fmt=info:ofi/fmt:kev:mtx:dissertation&res_dat=xri:pqm&rft_dat=xri:pqdiss:3510708 | 2. It is a reading-related technology but it is not a training (test, support…) |
| Enslinn, A. (2014). Literary Gaming. Cambridge, Massachusetts; London, England: The MIT Press. Retrieved from http://ezproxy.si.unav.es:2048/login?url=http://search.ebscohost.com/login.aspx?direct=true&AuthType=ip,url&db=edsjbk&AN=edsjbk.j.ctt9qf59z&lang=es&site=eds-live&scope=site | 2. It is a reading-related technology but it is not a training (test, support…) |
| Eroglu, G., Aydin, S., Cetin, M., & Balcisoy, S. (2018). Improving cognitive functions of dyslexies using multi-sensory learning and EEG neurofeedback. 26th IEEE Signal Processing and Communications Applications Conference, SIU 2018, 1–4. https://doi.org/10.1109/SIU.2018.8404711 | 7. Technological reading training that do not fulfill methods requirements |
| Eroglu, G., Cetin, M., & Balcisoy, S. (2018). Electroencephalographic identifiers of reading abilities in turkish language. 26th IEEE Signal Processing and Communications Applications Conference, SIU 2018, 1–4. https://doi.org/10.1109/SIU.2018.8404701 | 1.Non technological reading training |
| Erol, B., Antúnez, E., & Hull, J. J. (2008). Hotpaper. In Proceeding of the 16th ACM international conference on Multimedia - MM ’08 (p. 399). RICOH Innovations, California Research Center, 2882 Sand Hill Road, Menlo Park, CA, United States. https://doi.org/10.1145/1459359.1459413 | 2. It is a reading-related technology but it is not a training (test, support…) |
| Ersan, M. (2016). Picture Books on Tablet Devices; an Analysis on Form and Content Features. Turkish Online Journal of Design Art and Communication, 6(2), 148–158. https://doi.org/10.7456/10602100/009 | 5. Technology but it is not neither a training or reading-related |
| Ersek, M., & Wood, B. B. (2008). Development and evaluation of a Nursing Assistant Computerized Education Programme. International Journal of Palliative Nursing, 14(10), 502–509. https://doi.org/10.12968/ijpn.2008.14.10.31495 | 10. Educational-related but it is not about reading |
| Estes, M. E. (2013). Surprises. AALL Spectrum, 17(4), 1. Retrieved from http://ezproxy.si.unav.es:2048/login?url=http://search.ebscohost.com/login.aspx?direct=true&AuthType=ip,url&db=edo&AN=87367085&lang=es&site=eds-live&scope=site | 4. Reading-related but it is not neither a training or technological |
| Evans, J. (2012). Emerging Technologies to Consider. Intercom, 30(6), 6–8. https://doi.org/10.1016/j.gerinurse.2009.08.010 | 5. Technology but it is not neither a training or reading-related |
| Evans, S., Davis, K., Evans, A., Campbell, J. A., Randall, D. P., Yin, K., & Aragon, C. (2017). More Than Peer Production: Fanfiction Communities as Sites of Distributed Mentoring. In Proceedings of the 2017 ACM Conference on Computer Supported Cooperative Work and Social Computing (pp. 259–272). University of Washington, Seattle, United States: Association for Computing Machinery. https://doi.org/10.1145/2998181.2998342 | 2. It is a reading-related technology but it is not a training (test, support…) |
| Fahimuddin, F. Z., Sidhu, S., Agrawal, A., Fatimah Z., F., Sanam, S., & Anubhav, A. (2019). Reading Level of Online Patient Education Materials From Major Obstetrics and Gynecology Societies. Obstetrics & Gynecology, 133(5), 987–993. https://doi.org/10.1097/AOG.0000000000003214 | 10. Educational-related but it is not about reading |
| Farah, R. N., Hanani, F. N., & Zuraida, R. L. (2019). Linprot: A multimedia courseware for optimization methods with integration of augmented reality. International Journal of Scientific and Technology Research, 8(10), 2558–2563. https://www.scopus.com/inward/record.uri?eid=2-s2.0-85074604708&partnerID=40&md5=46e7ff45d8e953842d1dc70e6a439609 | 3. Technological training that is not focus on improving reading |
| Farley, K. T. (2008). Teaching performance in the digital age: Computerized technologies, improvisational play techniques and interactive learning processes. Dissertation Abstracts International Section A: Humanities and Social Sciences. ProQuest Information & Learning, US. Retrieved from http://0-search.ebscohost.com.library.ucc.ie/login.aspx?direct=true&db=psyh&AN=2008-99050-587&site=ehost-live | 3. Technological training that is not focus on improving reading |
| Fatany, H. (2012). Effectiveness of Mobile Learning in the Developing of Mathematical Problems Solving Skills of Students Second Grade Average. Arab Gulf Journal of Scientific Research, 30(4), 192–198. | 3. Technological training that is not focus on improving reading |
| Fenty, N., Mulcahy, C., & Washburn, E. (2015). Effects of computer-assisted and teacher-led fluency instruction on students at risk for reading failure. Learning Disabilities: A Contemporary Journal. ProQuest Information & Learning, US. Retrieved from http://ezproxy.si.unav.es:2048/login?url=http://search.ebscohost.com/login.aspx?direct=true&AuthType=ip,url&db=psyh&AN=2008-99050-264&lang=es&site=eds-live&scope=site | 7. Technological reading training that do not fulfill methods requirements |
| Ferrari, S. (2013). From Generative to Conventional Play: {MOBA} and {League of Legends}. In Proceedings of {DiGRA} 2013: DeFragging Game Studies. Georgia Institute of Technology, 85 Fifth Street NW, Atlanta, GA, United States: Digital Games Research Association (DiGRA). Retrieved from https://www.scopus.com/inward/record.uri?eid=2-s2.0-85000634219&partnerID=40&md5=ffbbea2bdce09b41c20d7d3d1c56a790 | 5. Technology but it is not neither a training or reading-related |
| Ferreira, A., Elejalde, J., & Vine, A. (2014). Análisis de Errores Asistido por Computador basado en un Corpus de Aprendientes de Español como Lengua Extranjera. Revista Signos, 47(86), 385–411. https://doi.org/10.4067/S0718-09342014000300003 | 9. Training oriented to a second-language learning |
| Figueiredo, C. (2016). Production design and game design in videogames: Action, emotion and immersion in the player lived. (S. M. & R. F., Eds.), Advances in Intelligent Systems and Computing. CIAUD—Faculty of Architecture, University of Lisbon, Pólo Universitário, Rua Sá Nogueira, Alto da Ajuda, Lisbon, Portugal: Springer Verlag. https://doi.org/10.1007/978-3-319-41983-1_60 | 5. Technology but it is not neither a training or reading-related |
| Figueiredo, M., Gomes, J., Gomes, C. M. C., Gaspar, R., & Lopes, J. M. (2018). Augmented reality as a new media for supporting mobile-learning. In Virtual and Augmented Reality: Concepts, Methodologies, Tools, and Applications (Vol. 3). IGI Global. https://doi.org/10.4018/978-1-5225-5469-1.ch076 | 11. Type of study excluded (review, non-primary references…) |
| Figueiredo, M., Gomes, J., Gomes, C., & Lopes, J. (2014). Augmented Reality Tools and Learning Practice in Mobile-Learning. 8th International Conference, UAHCI 2014 Held as Part of HCI International 2014 Heraklion, Crete, Greece, June 22–27, 2014, Proceedings, Part II. Centro de Investigação Marinha e Ambiental, Portugal: Springer Verlag. https://doi.org/10.1007/978-3-319-07440-5_28 | 5. Technology but it is not neither a training or reading-related |
| Filho, J. F., & Prata, W. (2009). Improving static print design readability using mobile reading filters. Lecture Notes in Computer Science (Including Subseries Lecture Notes in Artificial Intelligence and Lecture Notes in Bioinformatics), 5614 LNCS(PART 1), 31–37. https://doi.org/10.1007/978-3-642-02707-9_4 | 2. It is a reading-related technology but it is not a training (test, support…) |
| Florit, E., Cain, K., & Mason, L. (2019). Going beyond children’s single-text comprehension: The role of fundamental and higher-level skills in 4 th graders’ multiple-document comprehension. British Journal of Educational Psychology. https://doi.org/10.1111/bjep.12288 | 2. It is a reading-related technology but it is not a training (test, support…) |
| Flowers, C., Kim, D.-H., Lewis, P., & Davis, V. C. (2011). A comparison of computer-based testing and pencil-and-paper testing for students with a read-aloud accommodation. Journal of Special Education Technology, 26(1), 1–12. Retrieved from http://www.tamcec.org/jset-index/a-comparison-of-computer-based-testing-and-pencil-and-paper-testing-for-students-with-a-read-aloud-accommodation/%5Cnhttp://www.tamcec.org/jset/ | 2. It is a reading-related technology but it is not a training (test, support…) |
| Follmer, S., Ballagas, R. (Tico), Raffle, H., Spasojevic, M., & Ishii, H. (2012). People in books. In Proceedings of the ACM 2012 conference on Computer Supported Cooperative Work - CSCW ’12 (p. 685). Tangible Media Group, MIT Media Lab., 77 Massachusetts Ave., Cambridge, MA 02139, United States. https://doi.org/10.1145/2145204.2145309 | 2. It is a reading-related technology but it is not a training (test, support…) |
| Fonseca, M. S., Costa Zaqueu, L. da C., Albuquerque Santos, M. J., & Bottentuit Junior, J. B. (2017). E-book as a teaching resource for students in the hospital class. REVISTA EDUCAONLINE, 11(1), 82–98. | 2. It is a reading-related technology but it is not a training (test, support…) |
| Forbes, B. E., Skinner, C. H., Black, M. P., Yaw, J., Booher, J., & Delisle, J. (2013). Learning rates and known-to-unknown flash-card ratios: Comparing effectiveness while holding instructional time constant. Journal of Applied Behavior Analysis. https://doi.org/10.1002/jaba.74 | 5. Technology but it is not neither a training or reading-related |
| Foreman, S. B. C. (2015). Assistive technologies used by students with Asperger’s syndrome to improve performance in the general education classroom. Dissertation Abstracts International Section A: Humanities and Social Sciences. ProQuest Information & Learning, US. Retrieved from http://gateway.proquest.com/openurl?url_ver=Z39.88-2004&rft_val_fmt=info:ofi/fmt:kev:mtx:dissertation&res_dat=xri:pqm&rft_dat=xri:pqdiss:3645915%5Cnhttp://ovidsp.ovid.com/ovidweb.cgi?T=JS&PAGE=reference&D=psyc12&NEWS=N&AN=2015-99170-415 | 8. Training oriented to special population (other than dyslexia) |
| Förster, N., & Souvignier, E. (2011). Curriculum-Based Measurement: Developing a Computer-Based Assessment Instrument for Monitoring Student Reading Progress on Multiple Indicators. Learning Disabilities -- A Contemporary Journal, 9(2), 65–88. Retrieved from http://ezproxy.georgetowncollege.edu:2048/login?URL=http://search.ebscohost.com/login.aspx?direct=true&db=a9h&AN=70425500 | 2. It is a reading-related technology but it is not a training (test, support…) |
| Fostick, L., Eshcoly, R., Shtibelman, H., Nehemia, R., & Levi, H. (2014). Efficacy of temporal processing training to improve phonological awareness among dyslexic and normal reading students. Journal of Experimental Psychology: Human Perception and Performance. https://doi.org/10.1037/a0037527 | 7. Technological reading training that do not fulfill methods requirements |
| Francese, R., Monaco, C., & Nicoletti, C. (2018). An Android application for helping in the identification of Children with Reading Difficulties. GOODTECHS `18: PROCEEDINGS OF THE 4TH EAI INTERNATIONAL CONFERENCE ON SMART OBJECTS AND TECHNOLOGIES FOR SOCIAL GOOD (GOODTECHS), 226–231. https://doi.org/10.1145/3284869.3284915 | 2. It is a reading-related technology but it is not a training (test, support…) |
| Francese, R., Monaco, C., & Nicoletti, C. (2018). An Android application for helping in the identification of Children with Reading Difficulties. GOODTECHS `18: PROCEEDINGS OF THE 4TH EAI INTERNATIONAL CONFERENCE ON SMART OBJECTS AND TECHNOLOGIES FOR SOCIAL GOOD (GOODTECHS), 226–231. https://doi.org/10.1145/3284869.3284915 | 2. It is a reading-related technology but it is not a training (test, support…) |
| Frederick, P. (2010). Using digital game-based learning to support vocabulary instruction for developmental reading students. ProQuest LLC. ProQuest LLC. Retrieved from https://www.lib.byu.edu/cgi-bin/remoteauth.pl?url=http://proquest.umi.com/pqdweb?did=1949628581&amp%5CnFmt=7&amp%5CnclientId=9469&amp%5CnRQT=309&amp%5CnVName=PQD | 7. Technological reading training that do not fulfill methods requirements |
| Fritschy, J.-M., & Sarter, M. (2017). The European Journal of Neuroscience from 2008 to 2014. European Journal of Neuroscience, 45(7), 875–876. https://doi.org/10.1111/ejn.13531 | 11. Type of study excluded (review, non-primary references…) |
| Frohner, M., Urbauer, P., & Sauermann, S. (2017). Bluetooth low energy peripheral android health app for educational and interoperability testing purposes. In H. D. & S. G. (Eds.), Studies in Health Technology and Informatics (Vol. 236, pp. 336–342). IOS Press. https://doi.org/10.3233/978-1-61499-759-7-336 | 5. Technology but it is not neither a training or reading-related |
| Fucekova, M., & Metruk, R. (2018). DEVELOPING ENGLISH SKILLS BY MEANS OF MOBILE APPLICATIONS. INFORMATION TECHNOLOGIES AND LEARNING TOOLS, 66(4), 173–185. https://doi.org/10.33407/itlt.v66i4.2376 | 9. Training oriented to a second-language learning |
| Gabay, Y., & Holt, L. L. (2015). Incidental learning of sound categories is impaired in developmental dyslexia. Cortex, 73, 131–143. https://doi.org/10.1016/j.cortex.2015.08.008 | 4. Reading-related but it is not neither a training or technological |
| Gamage, N. D. U., Jayadewa, K. W. C., Samarakoon, S. M. U. P. M. U. P., Udeshitha, K. L. A. D. L. A. D., Senanayake, S. M. N. K. B. M. N. K. B., Hennayake, T. M., Jayadewa, K. W. C., Senanayake, S. M. N. K. B. M. N. K. B., Samarakoon, S. M. U. P. M. U. P., & Hennayake, T. M. (2017). Press plus-interactive mobile application for effective news reading. ICCSE 2017 - 12th International Conference on Computer Science and Education, 380–385. https://doi.org/10.1109/ICCSE.2017.8085521 | 2. It is a reading-related technology but it is not a training (test, support…) |
| Gambacorta, C., Nahum, M., Vedamurthy, I., Bayliss, J., Jordan, J., Bavelier, D., & Levi, D. M. (2018). An action video game for the treatment of amblyopia in children: A feasibility study. https://doi.org/10.1016/j.visres.2018.04.005 | 8. Training oriented to special population (other than dyslexia) |
| Gao, T., & Zhang, L. (2013). The application of internet of things for intelligent electrical meter reading system. Sensors and Transducers, 158(11), 255–261. Retrieved from https://www.scopus.com/inward/record.uri?eid=2-s2.0-84900640966&partnerID=40&md5=551eaf96e037b87474dead8df3efc441 | 5. Technology but it is not neither a training or reading-related |
| García Rodríguez, A., & Gómez-Díaz, R. (2016). Niños y apps: aprendiendo a leer y escribir en digital. Alabe Revista de Investigación Sobre Lectura y Escritura, 7(13), 1–23. https://doi.org/10.15645/Alabe2016.13.6 | 11. Type of study excluded (review, non-primary references…) |
| Gauthier, C. A. (2014). Teacher Implementation of Response to Intervention for Grade 6 Students at Risk for Failure in Reading. ProQuest Dissertations and Theses. ProQuest Information & Learning, US. Retrieved from https://search.proquest.com/docview/1609399584?accountid=15272 | 4. Reading-related but it is not neither a training or technological |
| Gazzano, A. (2019). Improving Piano Sight-Reading in Beginners: An Audio-Visual Free App for Self-Practice. VIRTUALIDAD EDUCACION Y CIENCIA, 10(18), 117–126. | 3. Technological training that is not focus on improving reading |
| Gerber, H. R., & Onwuegbuzie, A. (2013). ``Why Can’T We Always Learn Like This?{’’} Games-Based Learning and the English Language Arts Classroom: Lessons Learned and Practical Applications. In Tan, DTH and Fang, LML (Ed.), 2013 Ieee 63Rd Annual Conference International Council for Educational Media (Icem). | 7. Technological reading training that do not fulfill methods requirements |
| Gerber, H. R., Abrams, S. S., Onwuegbuzie, A. J., & Benge, C. L. (2014). From Mario to FIFA: What qualitative case study research suggests about games-based learning in a US classroom. Educational Media International, 51(1), 16–34. https://doi.org/10.1080/09523987.2014.889402 | 7. Technological reading training that do not fulfill methods requirements |
| Gewertz, C. (2010). Study of Reading Programs Finds Little Proof of Gains In Student Comprehension. Education Week, 29(31), 6. Retrieved from http://ezproxy.si.unav.es:2048/login?url=http://search.ebscohost.com/login.aspx?direct=true&AuthType=ip,url&db=asx&AN=51214490&lang=es&site=eds-live&scope=site | 11. Type of study excluded (review, non-primary references…) |
| Ghouati, A. El. (2017). Examining the Relationship between E-Learning Styles and Achievement in English among Moroccan University Students. Arab World English Journal Arab World English Journal (AWEJ), 88(2), 323–332. https://doi.org/10.24093/awej/vol8no2.23 | 9. Training oriented to a second-language learning |
| Giakoumis, D., Kaklanis, N., Votis, K., & Tzovaras, D. (2014). Enabling user interface developers to experience accessibility limitations through visual, hearing, physical and cognitive impairment simulation. Universal Access in the Information Society, 13(2), 227–248. https://doi.org/10.1007/s10209-013-0309-0 | 5. Technology but it is not neither a training or reading-related |
| Gil, L., Martinez, T., & Vidal-Abarca, E. (2015). Online assessment of strategic reading literacy skills. Computers & Education, 82, 50–59. https://doi.org/10.1016/j.compedu.2014.10.026 | 2. It is a reading-related technology but it is not a training (test, support…) |
| Golke, S., Dörfler, T., & Artelt, C. (2015). The impact of elaborated feedback on text comprehension within a computer-based assessment. Learning and Instruction, 39, 123–136. https://doi.org/10.1016/j.learninstruc.2015.05.009 | 2. It is a reading-related technology but it is not a training (test, support…) |
| Golovchinksy, G., Carter, S., & Dunnigan, A. (2011). Ara. In Proceedings of the 19th ACM international conference on Multimedia - MM ’11 (p. 799). FX Palo Alto Laboratory, Inc., 3174 Porter Drive, Palo Alto, CA 94304, United States. https://doi.org/10.1145/2072298.2072464 | 2. It is a reading-related technology but it is not a training (test, support…) |
| Gomez, I., & Alvarez, J. M. G. (2017). PANTALLAS E INDUSTRIA ADAPTATION IN A MULTIMEDIATIC ERA: TEXTS, SCREENS AND INDUSTRY. FOTOCINEMA-REVISTA CIENTIFICA DE CINE Y FOTOGRAFIA, 14, 5–18. | 10. Educational-related but it is not about reading |
| Google’s new rivet app encourages children to read. (2019). Pakistan & Gulf Economist VO  - 38, 21. https://ezproxy.unav.es/login?url=https://search.ebscohost.com/login.aspx?direct=true&AuthType=ip,url&db=edsgbe&AN=edsgcl.586827877&lang=es&site=eds-live&scope=site | 10. Educational-related but it is not about reading |
| Grabe, W. (2008). Vocabulary and Reading Comprehension. Reading in a Second Language: Moving from Theory to Practice., 13(2), 531-540w. https://doi.org/10.1002/9781405198431.wbeal1267 | 2. It is a reading-related technology but it is not a training (test, support…) |
| Gutierrez, M. P. C., Cristobal, I. H. T., Alonzo, A. T. B., & Bustamante, R. R. M. (2019). Digital Storytelling vs Traditional Storytelling: Teaching English Language to ANHS Students. 2019 9th IEEE Integrated STEM Education Conference, ISEC 2019, 38–41. https://doi.org/10.1109/ISECon.2019.8882047 | 9. Training oriented to a second-language learning |
| Hahnel, C., Goldhammer, F., Kroehne, U., & Naumann, J. (2018). The role of reading skills in the evaluation of online information gathered from search engine environments. COMPUTERS IN HUMAN BEHAVIOR, 78, 223–234. https://doi.org/10.1016/j.chb.2017.10.004 | 4. Reading-related but it is not neither a training or technological |
| Haladjian, J., Richter, D., Muntean, P., Ismailović, D., & Brügge, B. (2013). A framework for the creation of mobile games targeting dyslexic children. IADIS Mobile Learning 2013. International Association for Development of the Information Society. Retrieved from http://ezproxy.si.unav.es:2048/login?url=http://search.ebscohost.com/login.aspx?direct=true&AuthType=ip,url&db=eric&AN=ED562385&lang=es&site=eds-live&scope=site | 2. It is a reading-related technology but it is not a training (test, support…) |
| Hall, J., & Bush, L. (2013). Incorporating the game of geocaching in K-12 classrooms and teacher education programs. In Pedagogical Applications and Social Effects of Mobile Technology Integration (Vol. 2, pp. 79–97). Mercer University, United States: IGI Global. https://doi.org/10.4018/978-1-4666-2985-1.ch005 | 3. Technological training that is not focus on improving reading |
| Hamdan, K., & Amorri, A. (2016). MOBILE LEARNING DEVICES TO ASSIST DYSLEXIC STUDENTS TO IMPROVE THEIR ENGLISH READING PROFICIENCY. In Chova, LG and Martinez, AL and Torres, IC (Ed.), INTED2016: 10TH INTERNATIONAL TECHNOLOGY, EDUCATION AND DEVELOPMENT CONFERENCE (pp. 8201–8213). | 7. Technological reading training that do not fulfill methods requirements |
| Hameister, I., Nickels, L., Abel, S., & Croot, K. (2017). “Do you have mowing the lawn?”–improvements in word retrieval and grammar following constraint-induced language therapy in primary progressive aphasia. Aphasiology, 31(3), 308–331. https://doi.org/10.1080/02687038.2016.1197558 | 8. Training oriented to special population (other than dyslexia) |
| Hamid, S. S. A., Admodisastro, N., & Ghani, A. A. A. (2015). Computer-based learning model to improve learning of the malay language amongst dyslexic primary school students. In Proceedings of the Asia Pacific HCI and UX Design Symposium on - APCHIUX ’15 (Vol. 07-Decembe, pp. 37–41). https://doi.org/10.1145/2846439.2846448 | 2. It is a reading-related technology but it is not a training (test, support…) |
| HAMSAH, R. A. (2018). DEVELOPING ONLINE READING MATERIAL USING TASK BASED LEARNING FOR THE TENTH GRADE STUDENTS AT MA MA’ARIF NU RANDEGANSARI DRIYOREJO GRESIK. https://ezproxy.unav.es/login?url=https://search.ebscohost.com/login.aspx?direct=true&AuthType=ip,url&db=edsbas&AN=edsbas.2D4A95FD&lang=es&site=eds-live&scope=site | 5. Technology but it is not neither a training or reading-related |
| Hao, Y. W., & Zeng, C. (2017). On reading changes under the fragmentized reading environment and application of visual thinking in the design of mobile multimedia reading interface. Proceedings - 2016 International Conference on Information System and Artificial Intelligence, ISAI 2016, 168–171. https://doi.org/10.1109/ISAI.2016.0044 | 2. It is a reading-related technology but it is not a training (test, support…) |
| Hargood, C., Hicks, B., Charles, F., Lynch, S., & Tang, W. (2017). Snow White Is Missing: An Interactive Locative Story for Dementia Patients. In Tian, F and Gatzidis, C and ElRhalibi, A and Tang, W and Charles, F (Ed.), E-LEARNING AND GAMES, EDUTAINMENT 2017 (Vol. 10345, pp. 85–92). https://doi.org/10.1007/978-3-319-65849-0_10 | 8. Training oriented to special population (other than dyslexia) |
| Hartmann, E. (2009). Erfolg versprechende Computerprogramme für Kinder und Jugendliche mit Lese-Rechtschreibschwierigkeiten: Ein Überblick. Sprache · Stimme · Gehör, 33(04), 203–211. https://doi.org/10.1055/s-0029-1242739 | 11. Type of study excluded (review, non-primary references…) |
| Harvey, H., & Walker, R. (2018). Reading comprehension and its relationship with working memory capacity when reading horizontally scrolling text. QUARTERLY JOURNAL OF EXPERIMENTAL PSYCHOLOGY, 71(9), 1887–1897. https://doi.org/10.1080/17470218.2017.1363258 | 2. It is a reading-related technology but it is not a training (test, support…) |
| Hashimoto, Y., Iikura, Y., Hisada, Y., Kang, S., Arisawa, T., & Kobayashi-Better, D. (2017). The Kuzushiji Project: Developing a Mobile Learning Application for Reading Early Modern Japanese Texts. DIGITAL HUMANITIES QUARTERLY, 11(1). | 2. It is a reading-related technology but it is not a training (test, support…) |
| Hassan Taj, I., Ali, F., Aslam Sipra, M., & Ahmad, W. (2017). Effect of Technology Enhanced Language Learning on Vocabulary Acquisition of EFL Learners. International Journal of Applied Linguistics and English Literature, 6(3), 262. https://doi.org/10.7575/aiac.ijalel.v.6n.3p.262 | 9. Training oriented to a second-language learning |
| Hautala, J., Heikkilä, R., Nieminen, L., Rantanen, V., Latvala, J.-M., & Richardson, U. (2020). Identification of Reading Difficulties by a Digital Game-Based Assessment Technology. Journal of Educational Computing Research, 073563312090530. https://doi.org/10.1177/0735633120905309 | 2. It is a reading-related technology but it is not a training (test, support…) |
| Hazaea, A. N., & Alzubi, A. A. (2016). The Effectiveness of Using Mobile on EFL Learners’ Reading Practices in Najran University. English Language Teaching, 9(5), 8. https://doi.org/10.5539/elt.v9n5p8 | 9. Training oriented to a second-language learning |
| Hazaea, A. N., & Alzubi, A. A. (2018). Impact of mobile assisted language learning on learner autonomy in EFL reading context. JOURNAL OF LANGUAGE AND EDUCATION, 4(2), 48–58. https://doi.org/10.17323/2411-7390-2018-4-2-48-58 | 9. Training oriented to a second-language learning |
| He, L., & Min, S. (2017). Development and Validation of a Computer Adaptive EFL Test. Language Assessment Quarterly, 14(2), 160–176. https://doi.org/10.1080/15434303.2016.1162793 | 9. Training oriented to a second-language learning |
| Head, T. S. (2016). Supporting literacy with iPads: A pilot study  in second-grade classrooms. ProQuest Dissertations and Theses. ProQuest Information & Learning, US. Retrieved from https://search.proquest.com/docview/1777617036?accountid=10673%0Ahttp://openurl.ac.uk/redirect/athens:edu/?url_ver=Z39.88-2004&rft_val_fmt=info:ofi/fmt:kev:mtx:dissertation&genre=dissertations+%26+theses&sid=ProQ:ProQuest+Dissertations+%26+Theses+Global&a | 3. Technological training that is not focus on improving reading |
| Hebbecker, K., & Souvignier, E. (2018). Formative Assessment in third-grade reading instruction—Implementation and effectiveness of a modular concept including prepared teaching-material [Formatives Assessments im Leseunterricht der Grundschule – Implementation und Wirksamkeit eines modularen, . Zeitschrift Fur Erziehungswissenschaft, 21(4), 735–765. https://doi.org/10.1007/s11618-018-0834-y | 2. It is a reading-related technology but it is not a training (test, support…) |
| Hebbecker, K., & Souvignier, E. (2018). Formative Assessment in third-grade reading instruction-Implementation and effectiveness of a modular concept including prepared teaching-material. ZEITSCHRIFT FUR ERZIEHUNGSWISSENSCHAFT, 21(4), 735–765. https://doi.org/10.1007/s11618-018-0834-y | 2. It is a reading-related technology but it is not a training (test, support…) |
| Hein, J. M., Teixeira, M. C. T. V., Seabra, A. G., & de Macedo, E. C. (2010). Assessment of efficacy of the phonic literacy software for students with intellectual disability . Revista Brasileira de Educacao Especial, 16(1), 65–82. Retrieved from https://www.scopus.com/inward/record.uri?eid=2-s2.0-77954884749&partnerID=40&md5=c84cb5f45cce4b6410b04180bb6e8926 | 8. Training oriented to special population (other than dyslexia) |
| Helland, T., Morken, F., Bless, J. J., Valderhaug, H. V, Eiken, M., Helland, W. A., & Torkildsen, J. V. (2018). Auditive training effects from a dichotic listening app in children with dyslexia. Dyslexia, 24(4), 336–356. https://doi.org/10.1002/dys.1600 | 3. Technological training that is not focus on improving reading |
| Helland, T., Tjus, T., Hovden, M., Ofte, S., & Heimann, M. (2011). Effects of Bottom-Up and Top-Down Intervention Principles in Emergent Literacy in Children at Risk of Developmental Dyslexia: A Longitudinal Study. Journal of Learning Disabilities, 44(2), 105–122. https://doi.org/10.1177/0022219410391188 | 7. Technological reading training that do not fulfill methods requirements |
| Hepworth, A. J. (2014). Influence of Student Engagement, Moods and Completed Assignments with on Normalized Gains and Growth in Reading Literature Using iPads. ProQuest LLC. ProQuest Information & Learning, US. Retrieved from http://ezproxy.si.unav.es:2048/login?url=http://search.ebscohost.com/login.aspx?direct=true&AuthType=ip,url&db=psyh&AN=2015-99110-278&lang=es&site=eds-live&scope=site | 2. It is a reading-related technology but it is not a training (test, support…) |
| Hernández Rentería, L. A., Ramírez Lujano, A., Contreras Davito, M., Carranza, D. B., & Peña Pérez Negrón, A. (2019). A serious game proposal to reinforce reading comprehension in scholars. Communications in Computer and Information Science, 1114 CCIS, 30–41. https://doi.org/10.1007/978-3-030-37386-3_3 | 7. Technological reading training that do not fulfill methods requirements |
| Herring, E., Grindle, C., & Kovshoff, H. (2019). Teaching early reading skills to children with severe intellectual disabilities using Headsprout Early Reading. JOURNAL OF APPLIED RESEARCH IN INTELLECTUAL DISABILITIES, 32(5), 1138–1148. https://doi.org/10.1111/jar.12603 | 8. Training oriented to special population (other than dyslexia) |
| Heuston, E. (2010). Effects of Computer-Based Early-Reading Academic Learning Time on Early-Reading Achievement: A Dose-Response Approach. Children. ProQuest LLC. Retrieved from http://contentdm.lib.byu.edu/ETD/image/etd3462.pdf | 7. Technological reading training that do not fulfill methods requirements |
| Hidayat, T., Darmawan, D., & Setiawati, L. (2019). PENGEMBANGAN MEDIA “SEMPOA DIGITAL BERBASIS M-LEARNING” PADA PELAJARAN MATEMATIKA DALAM POKOK BAHASAN ARITMATIKA. https://ezproxy.unav.es/login?url=https://search.ebscohost.com/login.aspx?direct=true&AuthType=ip,url&db=edsbas&AN=edsbas.77ED81ED&lang=es&site=eds-live&scope=site | 3. Technological training that is not focus on improving reading |
| Hilte, M., & Reitsma, P. (2011). Activating the meaning of a word facilitates the integration of orthography: Evidence from spelling exercises in beginning spellers. Journal of Research in Reading, 34(3), 333–345. https://doi.org/10.1111/j.1467-9817.2010.01442.x | 7. Technological reading training that do not fulfill methods requirements |
| Hilton-Prillhart, A. N., Hopkins, M. B., Skinner, C. H., & McCane-Bowling, S. (2011). Enhancing sight word reading in second-grade students using a computer-based sight word reading system. Journal of Evidence-Based Practices for Schools, 12(2), 205–218. Retrieved from http://search.ebscohost.com/login.aspx?direct=true&db=psyh&AN=2011-21662-008&site=ehost-live&scope=site%0AANPrillhart@milhgan.edu | 9. Training oriented to a second-language learning |
| Holl, K., & Elberzhager, F. (2016). Mobile Application Quality Assurance: Reading Scenarios as Inspection and Testing Support. In Proceedings - 42nd Euromicro Conference on Software Engineering and Advanced Applications, SEAA 2016 (pp. 245–249). Raunhofer IESE, Fraunhofer-Platz 1, Kaiserslautern, Germany: Institute of Electrical and Electronics Engineers Inc. https://doi.org/10.1109/SEAA.2016.11 | 5. Technology but it is not neither a training or reading-related |
| Holl, K., Scherr, S. A., & Elberzhager, F. (2018). Using Scenario-based Reading for Testing Mobile Applications with FIT4Apps. 2018 11TH INTERNATIONAL CONFERENCE ON THE QUALITY OF INFORMATION AND COMMUNICATIONS TECHNOLOGY (QUATIC), 175–183. https://doi.org/10.1109/QUATIC.2018.00035 | 5. Technology but it is not neither a training or reading-related |
| Holl, K., Scherr, S. A., & Elberzhager, F. (2018). Using scenario-based reading for testing mobile applications with FIT4Apps. Proceedings - 2018 International Conference on the Quality of Information and Communications Technology, QUATIC 2018, 175–183. https://doi.org/10.1109/QUATIC.2018.00035 | 3. Technological training that is not focus on improving reading |
| Holz, H., Brandelik, K., Beuttler, B., Brandelik, J., & Ninaus, M. (2018). How to train your syllable stress awareness. International Journal of Serious Games VO - 5, 3. https://doi.org/10.17083/ijsg.v5i3.242 | 2. It is a reading-related technology but it is not a training (test, support…) |
| Hooley, D. S., & Thorpe, J. (2017). The effects of formative reading assessments closely linked to classroom texts on high school reading comprehension. Educational Technology Research and Development. College of Education, Idaho State University, 921 S. 8th Ave., Pocatello, ID, United States: Springer New York LLC. https://doi.org/10.1007/s11423-017-9514-5 | 2. It is a reading-related technology but it is not a training (test, support…) |
| Hooley, D. S., Thorpe, J., Hooley 2 ), D. S. ( 1, Thorpe 4 ), J. ( 1, Hooley, D. S., & Thorpe, J. (2017). The effects of formative reading assessments closely linked to classroom texts on high school reading comprehension. Educational Technology Research and Development, 65(5), 1215–1238. https://doi.org/10.1007/s11423-017-9514-5 | 2. It is a reading-related technology but it is not a training (test, support…) |
| Horak, R., & Hrbacek, J. (2013). Elearning and mobile devices - Technical problems and possible solutions. In Szakal, A (Ed.), 2013 IEEE 11th International Conference on Emerging eLearning Technologies and Applications (ICETA) (pp. 123–126). Department of Technical Education and Information Science, Faculty of Education, Masaryk University, Brno, Czech Republic: IEEE Computer Society. https://doi.org/10.1109/ICETA.2013.6674416 | 10. Educational-related but it is not about reading |
| Hsu, C. K., Hwang, G. J., & Chang, C. K. (2013). A personalized recommendation-based mobile learning approach to improving the reading performance of EFL students. Computers and Education, 63, 327–336. https://doi.org/10.1016/j.compedu.2012.12.004 | 9. Training oriented to a second-language learning |
| Huang, H. -c. (2014). Online Versus Paper-based Instruction: Comparing Two Strategy Training Modules for Improving Reading Comprehension. RELC Journal, 45(2), 165–180. https://doi.org/10.1177/0033688214534797 | 9. Training oriented to a second-language learning |
| Huang, H.-C. (2013). iPad Reading: An Innovative Approach to New Literacies. In PACLIC 27 Workshop on Computer-Assisted Language Learning (pp. 520–525). National Taiwan Ocean University/2, Pei-ning Road, Keelung, Taiwan: National Chengchi University. Retrieved from http://aclweb.org/anthology/Y13-2002 | 9. Training oriented to a second-language learning |
| Hussain, A., Mutalib, N. A., & Yasin, A. (2014). JFakih: Modelling mobile learning game. In 2014 International Conference on Computer and Information Sciences, ICCOINS 2014 - A Conference of World Engineering, Science and Technology Congress, ESTCON 2014 - Proceedings. https://doi.org/10.1109/ICCOINS.2014.6868824 | 5. Technology but it is not neither a training or reading-related |
| Hvorecky, J. (2013). An Integral Approach to Online Education: An Example. Proceedings of the 12Th European Conference on E-Learning (Ecel 2013), 139–148. Retrieved from http://ezproxy.si.unav.es:2048/login?url=http://search.ebscohost.com/login.aspx?direct=true&AuthType=ip,url&db=edb&AN=101688191&lang=es&site=eds-live&scope=site | 10. Educational-related but it is not about reading |
| Hwang, G.-J., & Fu, Q.-K. (2019). Trends in the research design and application of mobile language learning: a review of 2007-2016 publications in selected SSCI journals. INTERACTIVE LEARNING ENVIRONMENTS, 27(4), 567–581. https://doi.org/10.1080/10494820.2018.1486861 | 11. Type of study excluded (review, non-primary references…) |
| Hyman, J. A., Moser, M. T., & Segala, L. N. (2014). Electronic reading and digital library technologies: Understanding learner expectation and usage intent for mobile learning. Educational Technology Research and Development, 62(1), 35–52. https://doi.org/10.1007/s11423-013-9330-5 | 2. It is a reading-related technology but it is not a training (test, support…) |
| Ibrahim, N. K., Hammed, H., Zaidan, A. A., Zaidan, B. B., Albahri, O. S., Alsalem, M. A., Mohammed, R. T., Jasim, A. N., Shareef, A. H., Jalood, N. S., Baqer, M. J., Nidhal, S., Almahdi, E. M., & Alaa, M. (2019). Multi-Criteria Evaluation and Benchmarking for Young Learners’ English Language Mobile Applications in Terms of LSRW Skills. IEEE ACCESS, 7, 146620–146651. https://doi.org/10.1109/ACCESS.2019.2941640 | 9. Training oriented to a second-language learning |
| Ide, W. T., Jorge, V., Silva, L., & Ruppert, G. C. S. (2014). Open-source STL library and application for Android mobile devices. In Bartolo, PJD and DeLemos, ACS and Pereira, AMH and Mateus, AJD and Ramos, C and DosSantos, C and Oliveira, D and Pinto, E and Craveiro, F and Bartolo, HMCDTG and Almeia, HD and Sousa, I and Matias, JM and Durao, L and Gaspar, M and Alves, NMF and Carreira (Ed.), HIGH VALUE MANUFACTURING: ADVANCED RESEARCH IN VIRTUAL AND RAPID PROTOTYPING (pp. 395–398). | 2. It is a reading-related technology but it is not a training (test, support…) |
| Imrattanatrai, W., Hanittinan, C., Tanachaihirunsiri, N., & Kamnoonwatana, N. (2014). Real-time recognition and augmented reality for education. In M. J.L. (Ed.), Proceedings of the 2014 3rd ICT International Senior Project Conference, ICT-ISPC 2014 (pp. 17–20). Faculty of Information and Communication Technology, Mahidol University, Nakorn Pathom, Thailand: Institute of Electrical and Electronics Engineers Inc. https://doi.org/10.1109/ICT-ISPC.2014.6923208 | 5. Technology but it is not neither a training or reading-related |
| Indonesian Sign Language Computer Application for the Deaf | 8. Training oriented to special population (other than dyslexia) |
| Iozzio, C. (2009). Six Alternatives to the Amazon Kindle 2. PC Magazine, 28(3), 1. Retrieved from http://search.ebscohost.com/login.aspx?direct=true&db=a9h&AN=42315571&amp%0Alang=ja&site=ehost-live | 2. It is a reading-related technology but it is not a training (test, support…) |
| Ipek, I. (2010). The effects of CBI lesson sequence type and field dependence on learning from computer-based cooperative instruction in web. Turkish Online Journal of Educational Technology, 9(1), 221–234. Retrieved from http://ezproxy.si.unav.es:2048/login?url=http://search.ebscohost.com/login.aspx?direct=true&AuthType=ip,url&db=eric&AN=EJ875785&lang=es&site=eds-live&scope=site | 2. It is a reading-related technology but it is not a training (test, support…) |
| Ipek, I. (2011). The effects of text density levels and the cognitive style of field dependence on learning from a CBI tutorial. Turkish Online Journal of Educational Technology, 10(1), 167–182. Retrieved from https://www.scopus.com/inward/record.uri?eid=2-s2.0-78751555833&partnerID=40&md5=4d664299c69c452b0be589f8aac4700c | 2. It is a reading-related technology but it is not a training (test, support…) |
| Irblich, D. (2015). Reading Games with Elves and Mathis. Computer Based Promotion of Reading for the first until fourth Classes. PRAXIS DER KINDERPSYCHOLOGIE UND KINDERPSYCHIATRIE, 64(10), 819–820. | 12. Other: No access granted, same data… |
| Irkhamudin, I., & Supriyanto, T. (2018). Development Reading Assessment Media to Understanding Javanese Language Computer Based on Test for Students on Javanese Language and Literature Department. https://ezproxy.unav.es/login?url=https://search.ebscohost.com/login.aspx?direct=true&AuthType=ip,url&db=edsbas&AN=edsbas.3803EDA5&lang=es&site=eds-live&scope=site | 2. It is a reading-related technology but it is not a training (test, support…) |
| Irwin, J., Preston, J., Brancazio, L., D’angelo, M., & Turcios, J. (2015). Development of an audiovisual speech perception app for children with autism spectrum disorders. Clinical Linguistics & Phonetics, 29(1), 76–83. https://doi.org/10.3109/02699206.2014.966395 | 8. Training oriented to special population (other than dyslexia) |
| Ishikawa, Y., Smith, C., Kondo, M., Akano, I., Maher, K., & Wada, N. (2014). Development and Use of an EFL Reading Practice Application for an Android Tablet Computer. (Chova, LG and Martinez, AL and Torres, IC, Ed.), International Journal of Mobile and Blended Learning (Vol. 6). International Association for Development of the Information Society. https://doi.org/10.4018/ijmbl.2014070103 | 9. Training oriented to a second-language learning |
| IURead: A new computer-based reading test | 2. It is a reading-related technology but it is not a training (test, support…) |
| J., K., H., G., & Y., S. (Eds.). (2016). International Workshop on Database Systems for Advanced Applications, DASFAA 2016. In Lecture Notes in Computer Science (including subseries Lecture Notes in Artificial Intelligence and Lecture Notes in Bioinformatics) (Vol. 9645, pp. 1–393). Springer Verlag. http://www.scopus.com/inward/record.url?eid=2-s2.0-84964040272&partnerID=tZOtx3y1 | 11. Type of study excluded (review, non-primary references…) |
| J., K., T., K., Christoph, R., G., C., & E., L. (Eds.). (2015). 10th European Conference on Technology Enhanced Learning, EC-TEL 2015. In 10th European Conference on Technology Enhanced Learning, EC-TEL 2015 (Vol. 9307, pp. 1–646). Springer Verlag. http://www.scopus.com/inward/record.url?eid=2-s2.0-84944710819&partnerID=40&md5=11237863e70fb0d5083d548d6a5a800c | 11. Type of study excluded (review, non-primary references…) |
| J., V., S.F., O., T., I., & C., G. (Eds.). (2017). 23rd International Conference on Collaboration Technologies, CRIWG 2017: Vol. 10391 LNCS (pp. 1–259). Springer Verlag. https://www.scopus.com/inward/record.uri?eid=2-s2.0-85026206831&partnerID=40&md5=f371a7d24dabc6e23c2e1c443b257ba0 | 11. Type of study excluded (review, non-primary references…) |
| Jacelon, C. S., Gibbs, M. A., & Ridgway, J. V. (2016). Computer technology for self-management: A scoping review. Journal of Clinical Nursing, 25(9–10), 1179–1192. https://doi.org/10.1111/jocn.13221 | 11. Type of study excluded (review, non-primary references…) |
| Jackson, L. A., Eye, A. Von, Fitzgerald, H. E., Witt, E. A., & Zhao, Y. (2011). Computers in Human Behavior Internet use , videogame playing and cell phone use as predictors of children ’ s body mass index ( BMI ), body weight , academic performance , and social and overall self-esteem. Computers in Human Behavior, 27(1), 599–604. https://doi.org/10.1016/j.chb.2010.10.019 | 5. Technology but it is not neither a training or reading-related |
| Jackson, L. a., Eye, a. Von, Fitzgerld, H. E., Witt, E. a., & Zhao, Y. Z. Y. (2009). Information Technology (IT) Use and Children’s Academic Performance. In 2009 Fourth International Conference on Internet and Web Applications and Services (pp. 533–538). All of Michigan State University, East Lansing, MI 48824, United States. https://doi.org/10.1109/ICIW.2009.86 | 2. It is a reading-related technology but it is not a training (test, support…) |
| Jackson, L. A., Eye, A. Von, Witt, E. A., Zhao, Y., & Fitzgerald, H. E. (2011). Computers in Human Behavior A longitudinal study of the effects of Internet use and videogame playing on academic performance and the roles of gender , race and income in these relationships. Computers in Human Behavior, 27(1), 228–239. https://doi.org/10.1016/j.chb.2010.08.001 | 5. Technology but it is not neither a training or reading-related |
| Jackson, L., Witt, E. A., Eye, A. von, Fitzgerald, H. E., & Zhao, Y. (2010). Children’s Information Technology (IT) Use and Their Physical, Cognitive, Social and Psychological Well-Being. In 2010 Fourth International Conference on Digital Society (pp. 198–203). Michigan State University, East Lansing, MI 48824, United States. https://doi.org/10.1109/ICDS.2010.69 | 2. It is a reading-related technology but it is not a training (test, support…) |
| Jacquet, J.-M., Linden, I., & Staicu, M.-O. (2012). Blackboard Rules for Coordinating Context-aware Applications in Mobile Ad Hoc Networks. Electronic Proceedings in Theoretical Computer Science, 91(Special Section on Foundations of Coordination Languages and Software (FOCLASA 2012)), 63–78. https://doi.org/10.4204/EPTCS.91.5 | 5. Technology but it is not neither a training or reading-related |
| Jacquin-Courtois, S., Bays, P. M., Salemme, R., Leff, A. P., & Husain, M. (2013). Rapid compensation of visual search strategy in patients with chronic visual field defects. Cortex, 49(4), 994–1000. https://doi.org/10.1016/j.cortex.2012.03.025 | 6. Training that is not neither technological or reading-related. |
| Jaén, M. M., & Basanta, C. P. (2009). Vocabulary and web-based instruction: A psycholinguistic perspective. International Journal of Learning, 16(9), 537–550. Retrieved from https://www.scopus.com/inward/record.uri?eid=2-s2.0-77950324105&partnerID=40&md5=cfe06e989a66562d52828159f8d26fb2 | 2. It is a reading-related technology but it is not a training (test, support…) |
| Jagoda, P. (2013). Fabulously procedural: Braid, historical processing, and the videogame sensorium. American Literature. https://doi.org/10.1215/00029831-2367346 | 5. Technology but it is not neither a training or reading-related |
| Jainis, J. Bin, & Taisi, N. J. B. (2018). LEARNING APPROACH OF READING THE KADAZANDUSUN LANGUAGE THROUGH SONGS AND COMPUTER-BASED MUSIC FOR STANDARD ONE STUDENT. https://doi.org/10.7454/irhs.v3i1.38 | 7. Technological reading training that do not fulfill methods requirements |
| Jairam, D., & Kiewra, K. A. (2010). Helping students soar to success on computers: An investigation of the SOAR study method for computer-based learning. Journal of Educational Psychology, 102(3), 601–614. https://doi.org/10.1037/a0019137 | 3. Technological training that is not focus on improving reading |
| Jakobsen, M., Meyer DeMott, M. A., & Heir, T. (2017). Validity of screening for psychiatric disorders in unaccompanied minor asylum seekers: Use of computer-based assessment. Transcultural Psychiatry, 1363461517722868. https://doi.org/10.1177/1363461517722868 | 8. Training oriented to special population (other than dyslexia) |
| Jalaliniya, S., & Mardanbegi, D. (2016). EyeGrip. In Proceedings of the 2016 CHI Conference on Human Factors in Computing Systems - CHI ’16 (pp. 5801–5811). IT University of Copenhagen, Copenhagen, Denmark: Association for Computing Machinery. https://doi.org/10.1145/2858036.2858584 | 2. It is a reading-related technology but it is not a training (test, support…) |
| Jankowski, J., Samp, K., Irzynska, I., Jozwowicz, M., & Decker, S. (2010). Integrating Text with Video and 3D Graphics: The Effects of Text Drawing Styles on Text Readability. In Proceedings of the 28th international conference on Human factors in computing systems (Vol. 2, pp. 1321–1330). Digital Enterprise Research Institute, National University of Ireland, Galway, Ireland. https://doi.org/10.1145/1753326.1753524 | 2. It is a reading-related technology but it is not a training (test, support…) |
| Jariwala, N., & Patel, B. (2018). A system for the conversion of digital Gujarati text-to-speech for visually impaired people. Advances in Intelligent Systems and Computing, 664, 67–75. https://doi.org/10.1007/978-981-10-6626-9_8 | 8. Training oriented to special population (other than dyslexia) |
| Javorsky, K., & Trainin, G. (2014). Teaching Young Readers to Navigate a Digital Story When Rules Keep Changing. Reading Teacher, 67(8), 606–618. https://doi.org/10.1002/trtr.1259 | 2. It is a reading-related technology but it is not a training (test, support…) |
| Jayashree, D., Afritha Farhath, K., Amruthavarshini, R., & Pavithra, S. (2016). Voice based application as medicine spotter for visually impaired. In 2016 2nd International Conference on Science Technology Engineering and Management, ICONSTEM 2016 (pp. 56–60). https://doi.org/10.1109/ICONSTEM.2016.7560923 | 5. Technology but it is not neither a training or reading-related |
| Jia, C. X., Liu, C., Liu, R. R., & Wang, P. (2012). Application of recommendation system: An empirical study of the mobile reading platform. Lecture Notes in Computer Science (Including Subseries Lecture Notes in Artificial Intelligence and Lecture Notes in Bioinformatics). Institute for Information Economy, Alibaba Business College, HangZhou Normal University, Zhejiang 310036, China. https://doi.org/10.1007/978-3-642-34624-8_45 | 2. It is a reading-related technology but it is not a training (test, support…) |
| Jia, W. (2017). Enlightenment from the Innovative Application of 4G Communication Technology in the Mobile Library. In Proceedings - 2016 International Conference on Smart City and Systems Engineering, ICSCSE 2016 (pp. 153–156). Hebi Polytechnic College, China: Institute of Electrical and Electronics Engineers Inc. https://doi.org/10.1109/ICSCSE.2016.0050 | 2. It is a reading-related technology but it is not a training (test, support…) |
| Jiang, X., Sawaki, Y., & Sabatini, J. (2012). Word reading efficiency, text reading fluency, and reading comprehension among Chinese learners of English. Reading Psychology, 33(4), 323–349. https://doi.org/10.1080/02702711.2010.526051 | 9. Training oriented to a second-language learning |
| Jiao, H., & Wang, S. (2010). A multifaceted approach to investigating the equivalence of “computer based” and “paper and pencil” assessments; an example of reading diagnostics. Int. J. Learn. Technol., 5(3), 264–288. https://doi.org/10.1504/IJLT.2010.037307 | 2. It is a reading-related technology but it is not a training (test, support…) |
| Jiménez-García, M., & De Los Ángeles Martínez-Ortega, M. (2017). Use of a mobile application in teaching reading [El uso de una Aplicación Móvil en la Enseñanza de la Lectura]. Informacion Tecnologica, 28(1), 151–160. https://doi.org/10.4067/S0718-07642017000100015 | 7. Technological reading training that do not fulfill methods requirements |
| Jiménez-García, M., & Martínez-Ortega, M. de los Á. (2017). El Uso de una Aplicación Móvil en la Enseñanza de la Lectura. Información tecnológica, 28(1), 151–160. https://doi.org/10.4067/S0718-07642017000100015 | 7. Technological reading training that do not fulfill methods requirements |
| Jiménez-Porta, A., & Diez-Martínez, E. (2016). DYSLEXIA : Analysis of technological resources (Mobile applications, PC applications, websites) in mexican Spanish to support its therapeutic in basic education. In I. Chova, LG and Martinez, AL and Torres (Ed.), INTED2016: 10TH INTERNATIONAL TECHNOLOGY, EDUCATION AND DEVELOPMENT CONFERENCE (pp. 5297–5305). | 11. Type of study excluded (review, non-primary references…) |
| Jin, N. (2017). Mobile-assisted language learning: Using WeChat in an English reading class. Lecture Notes in Computer Science (Including Subseries Lecture Notes in Artificial Intelligence and Lecture Notes in Bioinformatics), 10676 LNCS, 500–506. https://doi.org/10.1007/978-3-319-71084-6_59 | 2. It is a reading-related technology but it is not a training (test, support…) |
| Jin, N. (2017). Mobile-assisted language learning: Using WeChat in an English reading class. Lecture Notes in Computer Science (Including Subseries Lecture Notes in Artificial Intelligence and Lecture Notes in Bioinformatics), 10676 LNCS, 500–506. https://doi.org/10.1007/978-3-319-71084-6_59 | 2. It is a reading-related technology but it is not a training (test, support…) |
| Jobke, S., Kasten, E., & Sabel, B. A. (2009). Vision Restoration Through Extrastriate Stimulation in Patients With Visual Field Defects: A Double-Blind and Randomized Experimental Study. Neurorehabilitation and Neural Repair, 23(3), 246–255. https://doi.org/10.1177/1545968308324221 | 5. Technology but it is not neither a training or reading-related |
| Johnson, E., Pardini, J., Sandel, N., & Lovell, M. (2014). C-45 Do Athletes with Dyslexia Differ at Baseline and/or at Concussion Post-Injury Assessment on a Computer-Based Test Battery? Archives of Clinical Neuropsychology. https://doi.org/10.1093/arclin/acu038 | 4. Reading-related but it is not neither a training or technological |
| Johnson, M., & Nadas, R. (2009). Marginalised behaviour: Digital annotations, spatial encoding and the implications for reading comprehension. Learning, Media and Technology, 34(4), 323–336. https://doi.org/10.1080/17439880903338606 | 2. It is a reading-related technology but it is not a training (test, support…) |
| Jordan, C. O., & Rogers, D. L. (2015). Effect on blink rate during videogame related activities and reading in school-aged children. Journal of American Association for Pediatric Ophthalmology and Strabismus, 19(4), e48–e49. https://doi.org/10.1016/j.jaapos.2015.07.150 | 2. It is a reading-related technology but it is not a training (test, support…) |
| Jungjohann, J., DeVries, J. M., Gebhardt, M., & Muehling, A. (2018). Levumi: A Web-Based Curriculum-Based Measurement to Monitor Learning Progress in Inclusive Classrooms. In Miesenberger, K and Kouroupetroglou, G (Ed.), COMPUTERS HELPING PEOPLE WITH SPECIAL NEEDS, PT I (Vol. 10896, pp. 369–378). https://doi.org/10.1007/978-3-319-94277-3_58 | 2. It is a reading-related technology but it is not a training (test, support…) |
| Jungjohann, J., DeVries, J. M., Gebhardt, M., & Muehling, A. (2018). Levumi: A Web-Based Curriculum-Based Measurement to Monitor Learning Progress in Inclusive Classrooms. In Miesenberger, K and Kouroupetroglou, G (Ed.), COMPUTERS HELPING PEOPLE WITH SPECIAL NEEDS, PT I (Vol. 10896, pp. 369–378). https://doi.org/10.1007/978-3-319-94277-3_58 | 2. It is a reading-related technology but it is not a training (test, support…) |
| Kaltenegger, K., Kuester, S., Altpeter-Ott, E., Eschweiler, G. W., Cordey, A., Ivanov, I. V, Martus, P., Knipp, C., & Trauzettel-Klosinski, S. (2019). Effects of home reading training on reading and quality of life in AMDa randomized and controlled study. GRAEFES ARCHIVE FOR CLINICAL AND EXPERIMENTAL OPHTHALMOLOGY, 257(7), 1499–1512. https://doi.org/10.1007/s00417-019-04328-9 | 8. Training oriented to special population (other than dyslexia) |
| Kamijo, H., Morii, S., Yamaguchi, W., Toyooka, N., Tada-Umezaki, M., & Hirobayashi, S. (2016). Creating an Adaptive Technology Using a Cheminformatics System to Read Aloud Chemical Compound Names for People with Visual Disabilities. Journal of Chemical Education, 93(3), 496–503. https://doi.org/10.1021/acs.jchemed.5b00217 | 9. Training oriented to a second-language learning |
| Kast, M., Baschera, G.-M., Gross, M., Jäncke, L., & Meyer, M. (2011). Computer-based learning of spelling skills in children with and without dyslexia. Annals of Dyslexia, 61(2), 177–200. https://doi.org/10.1007/s11881-011-0052-2 | 7. Technological reading training that do not fulfill methods requirements |
| Kasten, E., Haschke, P., Meinhold, U., & Oertel-Verweyen, P. (2010). A computer program for training eccentric reading in persons with central scotoma. Journal of Visual Impairment and Blindness, 104(5), 303–311. Retrieved from http://www.afb.org/afbpress/pubjvib.asp?DocID=jvib040506 | 8. Training oriented to special population (other than dyslexia) |
| Kaznin, A. A., Sushko, O. P., & Babkin, A. V. (2017). Developing the Algorithm Allowing Business-Dedicated Mobile Applications to Read Texts. PROCEEDINGS OF THE 2017 INTERNATIONAL CONFERENCE QUALITY MANAGEMENT,TRANSPORT AND INFORMATION SECURITY, INFORMATION TECHNOLOGIES (IT&QM&IS), 210–217. | 2. It is a reading-related technology but it is not a training (test, support…) |
| Keegan, R. D., Oliver, M. C., Stanfill, T. J., Stevens, K. V, Brown, G. R., Ebinger, M., & Gay, J. M. (2016). Use of a mobile device simulation as a preclass active learning exercise. Journal of Nursing Education, 55(1), 56–59. https://doi.org/10.3928/01484834-20151214-14 | 10. Educational-related but it is not about reading |
| Keengwe, J., & Maxfield, M. (2014). Advancing higher education with mobile learning technologies: Cases, trends, and inquiry-based methods. Advancing Higher Education with Mobile Learning Technologies: Cases, Trends, and Inquiry-Based Methods (Vol. i). IGI Global. https://doi.org/10.4018/978-1-4666-6284-1 | 3. Technological training that is not focus on improving reading |
| Keezhatta, M. S., & Omar, A. (2019). Enhancing Reading Skills for Saudi Secondary School Students through Mobile Assisted Language Learning (MALL): An Experimental Study. INTERNATIONAL JOURNAL OF ENGLISH LINGUISTICS, 9(1), 437–447. https://doi.org/10.5539/ijel.v9n1p437 | 9. Training oriented to a second-language learning |
| Keller, K., Keough, W., & Galgao, F. (2018). Student Response to Use of Reading Assistant Software forStudent Response to Use of Reading Assistant Software forEnglish Language Learners in Thailand. https://doi.org/10.35974/isc.v6i1.1252 | 9. Training oriented to a second-language learning |
| Kerry, S. J., Aguilar, O. M., Penny, W., Crinion, J. T., Leff, A. P., & Woodhead, Z. V. J. (2019). How Does iReadMore Therapy Change the Reading Network of Patients with Central Alexia? The Journal of Neuroscience : The Official Journal of the Society for Neuroscience, 39(29), 5719–5727. https://doi.org/10.1523/JNEUROSCI.1426-18.2019 | 8. Training oriented to special population (other than dyslexia) |
| Kesim, M., & Yildirim, H. (2017). A LITERATURE REVIEW AND CONTENT ANALYSIS ON INTERACTIVE E-BOOKS. In Chova, LG and Martinez, AL and Torres, IC (Ed.), 9TH INTERNATIONAL CONFERENCE ON EDUCATION AND NEW LEARNING TECHNOLOGIES (EDULEARN17) (pp. 9824–9829). | 11. Type of study excluded (review, non-primary references…) |
| Kettler, R. J. (2011). Computer-Based Screening for the New Modified Alternate Assessment. Journal of Psychoeducational Assessment, 29(1), 3–13. https://doi.org/10.1177/0734282910370804 | 3. Technological training that is not focus on improving reading |
| Kheiravar, S., Lasserre, P., & Campbell, R. (2012). A mobile application for collaborative learning. In Proceedings of the 14th international conference on Human-computer interaction with mobile devices and services companion (pp. 137–142). Irving K. Barber School of Arts and Sciences, University of British Columbia, Kelowna, BC V1V 1V7, Canada. https://doi.org/10.1145/2371664.2371690 | 3. Technological training that is not focus on improving reading |
| Khezrlou, S., & Ellis, R. (2017). Effects of computer-assisted glosses on EFL learners’ vocabulary acquisition and reading comprehension in three learning conditions. System, 65, 104–116. https://doi.org/10.1016/j.system.2017.01.009 | 9. Training oriented to a second-language learning |
| Khowaja, K., & Salim, S. S. (2013). Research in Autism Spectrum Disorders A systematic review of strategies and computer-based intervention ( CBI ) for reading comprehension of children with autism. Research in Autism Spectrum Disorders. https://doi.org/10.1016/j.rasd.2013.05.009 | 8. Training oriented to special population (other than dyslexia) |
| Khowaja, K., Salim, S. S., & Al-Thani, D. (2018). Components to design serious games for children with autism spectrum disorder (ASD) to learn vocabulary. 2018 5TH IEEE INTERNATIONAL CONFERENCE ON ENGINEERING TECHNOLOGIES AND APPLIED SCIENCES (IEEE ICETAS). | 8. Training oriented to special population (other than dyslexia) |
| Khowaja, K., Salim, S. S., Asemi, A., Ghulamani, S., & Shah, A. (2019). A systematic review of modalities in computer-based interventions (CBIs) for language comprehension and decoding skills of children with autism spectrum disorder (ASD). Universal Access in the Information Society. https://doi.org/10.1007/s10209-019-00646-1 | 11. Type of study excluded (review, non-primary references…) |
| Ki, T., Munipalle, S., Dantu, K., Ko, S. Y., & Ziarek, L. (2014). Poster. In Proceedings of the 12th annual international conference on Mobile systems, applications, and services - MobiSys ’14 (pp. 373–373). Department of Computer Science and Engineering, University at Buffalo, State University of New York, Buffalo, NY, United States: Association for Computing Machinery. https://doi.org/10.1145/2594368.2601453 | 5. Technology but it is not neither a training or reading-related |
| Kim, B. S., & Min, S. L. (2017). QoS- aware Flash Memory Controller. In Parmer, G (Ed.), PROCEEDINGS OF THE 23RD IEEE REAL-TIME AND EMBEDDED TECHNOLOGY AND APPLICATIONS SYMPOSIUM (RTAS 2017) (pp. 51–61). | 5. Technology but it is not neither a training or reading-related |
| Kim, J. S., Kim, J. Y., Kim, K. T., Chae, J. B., Kim, J. H., & Kim, D. Y. (2019). Near reading speed changes after panretinal photocoagulation in diabetic retinopathy patients: a prospective study using an iPad application for the measurement of reading speed. Graefe’s Archive for Clinical and Experimental Ophthalmology, 257(12), 2631–2638. https://doi.org/10.1007/s00417-019-04494-w | 8. Training oriented to special population (other than dyslexia) |
| Kingsnorth, A., & Wolffsohn, J. S. (2015). Mobile app reading speed test. British Journal of Ophthalmology, 99(4), 536–539. https://doi.org/10.1136/bjophthalmol-2014-305818 | 1.Non technological reading training |
| Kiyani, H. S., & Naz, S. (2018). DEVELOPMENT OF NAMING, READING AND IMITATION SKILLS MANAGEMENT PROGRAMME FOR PATIENTS WITH BROCA’S APHASIA. Khyber Medical University Journal, 10(3), 127. https://ezproxy.unav.es/login?url=https://search.ebscohost.com/login.aspx?direct=true&AuthType=ip,url&db=edsbas&AN=edsbas.C99A264B&lang=es&site=eds-live&scope=site | 9. Training oriented to a second-language learning |
| Komatsu, H., & Rappleye, J. (2017). Did the shift to computer-based testing in PISA 2015 affect reading scores? A View from East Asia. COMPARE-A JOURNAL OF COMPARATIVE AND INTERNATIONAL EDUCATION, 47(4), 616–623. https://doi.org/10.1080/03057925.2017.1309864 | 10. Educational-related but it is not about reading |
| Kroehne, U., Buerger, S., Hahnel, C., & Goldhammer, F. (2019). Construct Equivalence of PISA Reading Comprehension Measured With Paper-Based and Computer-Based Assessments. EDUCATIONAL MEASUREMENT-ISSUES AND PRACTICE, 38(3), 97–111. https://doi.org/10.1111/emip.12280 | 4. Reading-related but it is not neither a training or technological |
| Kujala, T. (2009). Computer based reading game as a tool for improving reading related skills in Finnish six-year-old children. Frontiers in Human Neuroscience. Frontiers Media S.A. https://doi.org/10.3389/conf.neuro.09.2009.05.143 | 11. Type of study excluded (review, non-primary references…) |
| Kumar, K. (2018). Library in your pocket delivery of instruction service through library mobile apps: A world in your pocket. In Changing the Scope of Library Instruction in the Digital Age. IGI Global. https://doi.org/10.4018/978-1-5225-2802-9.ch009 | 5. Technology but it is not neither a training or reading-related |
| Kuzmičová, A., Schilhab, T., & Burke, M. (2018). m-Reading: Fiction reading from mobile phones. Convergence. https://doi.org/10.1177/1354856518770987 | 2. It is a reading-related technology but it is not a training (test, support…) |
| Kuznetsova, A. A., Ilyasova, L. G., Bezuglova, O. A., & Arbarova, S. A. (2017). Difficulty In English Reading Texts Comprehension. MODERN JOURNAL OF LANGUAGE TEACHING METHODS, 7(12), 21–30. | 4. Reading-related but it is not neither a training or technological |
| la Gala Quispe, K., & Vera Sancho, J. (2018). Use of augmented reality improves reading comprehension levels in fifth grade students of the primary level [Uso de la realidad aumentada mejora los niveles de comprensión lectora en estudiantes de quinto grado del nivel primario]. In Z.-R. M. G.-P. F. J. Villalba-Condori K.O. Lavonen J. (Ed.), CEUR Workshop Proceedings (Vol. 2302). CEUR-WS. https://www.scopus.com/inward/record.uri?eid=2-s2.0-85060627709&partnerID=40&md5=340dbbe13e6c2e545bf163dfa2cd1615 | 7. Technological reading training that do not fulfill methods requirements |
| Laborda, J. G., Royo, T. M., & Bakieva, M. (2016). Looking towards the future of language assessment: Usability of tablet PCs in language testing. Journal of Universal Computer Science, 22(1), 114–123. Retrieved from https://www.scopus.com/inward/record.uri?eid=2-s2.0-84964707326&partnerID=40&md5=bee67e9636cc14a46e6d8e398d1dd652 | 2. It is a reading-related technology but it is not a training (test, support…) |
| Lai, C. F., Tsai, C. W., Chen, S. Y., Hwang, R. H., & Yang, C. S. (2017). An intelligent concept map for e-book via automatic keyword extraction. In G. R., C. Y., H. Y.-M., W. T.-T., & X. H. (Eds.), Lecture Notes in Computer Science (including subseries Lecture Notes in Artificial Intelligence and Lecture Notes in Bioinformatics): Vol. 10108 LNCS (pp. 75–85). Springer Verlag. https://doi.org/10.1007/978-3-319-52836-6_10 | 2. It is a reading-related technology but it is not a training (test, support…) |
| Lan, Y. J., Sung, Y. T., & Chang, K. E. (2009). Let us read together: Development and evaluation of a computer-assisted reciprocal early English reading system. Computers and Education, 53(4), 1188–1198. https://doi.org/10.1016/j.compedu.2009.06.002 | 3. Technological training that is not focus on improving reading |
| Landerer, D., Bahro, D., Roehm, H., Koppitz, M., Mertens, A., Manger, F., Denk, F., Heidinger, M., Windmann, T., & Colsmann, A. (2017). Solar Glasses: A Case Study on Semitransparent Organic Solar Cells for Self-Powered, Smart, Wearable Devices. ENERGY TECHNOLOGY, 5(11), 1936–1945. https://doi.org/10.1002/ente.201700226 | 5. Technology but it is not neither a training or reading-related |
| Latimer, N. (2018). Reading during an academic reading-into-writing task: an eye-tracking study. University of Bedfordshire. https://ezproxy.unav.es/login?url=https://search.ebscohost.com/login.aspx?direct=true&AuthType=ip,url&db=edsbas&AN=edsbas.5E76C6D9&lang=es&site=eds-live&scope=site | 2. It is a reading-related technology but it is not a training (test, support…) |
| Lawton, T., & Shelley-Tremblay, J. (2017). Training on Movement Figure-Ground Discrimination Remediates Low-Level Visual Timing Deficits in the Dorsal Stream, Improving High-Level Cognitive Functioning, Including Attention, Reading Fluency, and Working Memory. FRONTIERS IN HUMAN NEUROSCIENCE, 11. https://doi.org/10.3389/fnhum.2017.00236 | 3. Technological training that is not focus on improving reading |
| Lawton, T., & Shelley-Tremblay, J. (2017). Training on Movement Figure-Ground Discrimination Remediates Low-Level Visual Timing Deficits in the Dorsal Stream, Improving High-Level Cognitive Functioning, Including Attention, Reading Fluency, and Working Memory. FRONTIERS IN HUMAN NEUROSCIENCE, 11. https://doi.org/10.3389/fnhum.2017.00236 | 7. Technological reading training that do not fulfill methods requirements |
| Lawton, T., & Shelley-Tremblay, J. (2018). “Training on movement figure-ground discrimination remediates low-level visual timing deficits in the dorsal stream, improving high-level cognitive functioning, including attention, reading fluency, and working memory”: Corrigendum. Frontiers in Human Neuroscience, 12. https://doi.org/10.3389/fnhum.2018.00461 | 11. Type of study excluded (review, non-primary references…) |
| Lazzeri, S., Cabezas, X., Ojeda, L., & Leiva, F. (2015). Assessing the Impact of Computer-Based Formative Evaluations in a Course of English as a Foreign Language for Undergraduate Kinesiology Students in Chile. Research-publishing.net. Research-publishing.net. Retrieved from http://search.ebscohost.com/login.aspx?direct=true&db=eric&AN=ED564250&site=ehost-live | 9. Training oriented to a second-language learning |
| Leger, P.-M., Nguyen, T. A., Charland, P., Senecal, S., Lapierre, H. G., Fredette, M., Léger, P.-M., An Nguyen, T., Charland, P., Sénécal, S., Lapierre, H. G., & Fredette, M. (2019). How Learner Experience and Types of Mobile Applications Influence Performance: The Case of Digital Annotation. Computers in the Schools, 36(2), 83–104. https://doi.org/10.1080/07380569.2019.1601957 | 2. It is a reading-related technology but it is not a training (test, support…) |
| Lena, P. H., Jimbara, W. R., & Suyoto. (2018). Learning to Read and Identifies the Level of Hearing Disability Early Age Using a Mobile Learning Application. In Auer, ME and Guralnick, D and Simonics, I (Ed.), TEACHING AND LEARNING IN A DIGITAL WORLD, VOL 1 (Vol. 715, pp. 355–362). https://doi.org/10.1007/978-3-319-73210-7_43 | 5. Technology but it is not neither a training or reading-related |
| Lenhard, W., Schroeders, U., & Lenhard, A. (2017). Equivalence of Screen Versus Print Reading Comprehension Depends on Task Complexity and Proficiency. DISCOURSE PROCESSES, 54(5–6, SI), 427–445. https://doi.org/10.1080/0163853X.2017.1319653 | 2. It is a reading-related technology but it is not a training (test, support…) |
| Leto, E. (2018). Supporting Dyslexic Students through ICT Tools in Foreign Language Learning. PROCEEDINGS OF THE 11TH INNOVATION IN LANGUAGE LEARNING INTERNATIONAL CONFERENCE, 146–149. | 9. Training oriented to a second-language learning |
| Levy, E. (2015, March 2). READING, WRITING, AND SWIPING. New York Family. Retrieved from http://ezproxy.si.unav.es:2048/login?url=http://search.ebscohost.com/login.aspx?direct=true&AuthType=ip,url&db=edb&AN=102061426&lang=es&site=eds-live&scope=site | 11. Type of study excluded (review, non-primary references…) |
| Lewandowski, L., Wood, W., & Miller, L. A. (2016). Technological Applications for Individuals with Learning Disabilities and ADHD. In Computer-Assisted and Web-Based Innovations in Psychology, Special Education, and Health (pp. 61–93). Department of Psychology, Syracuse University, New York, NY, United States: Elsevier Inc. https://doi.org/10.1016/B978-0-12-802075-3.00003-6 | 8. Training oriented to special population (other than dyslexia) |
| Li, X., & Yang, X. (2016). Effects of Learning Styles and Interest on Concentration and Achievement of Students in Mobile Learning. Journal of Educational Computing Research, 54(7), 922–945. https://doi.org/10.1177/0735633116639953 | 3. Technological training that is not focus on improving reading |
| Li, X.-G., Long, X.-L., Fan, L.-X., & Liao, Y.-M. (2018). A study of Assessment model of Oral English Imitation Reading in College Entrance Examination. In Li, W and Li, Q and Wang, L (Ed.), 2018 11TH INTERNATIONAL CONGRESS ON IMAGE AND SIGNAL PROCESSING, BIOMEDICAL ENGINEERING AND INFORMATICS (CISP-BMEI 2018). | 9. Training oriented to a second-language learning |
| Li, X.-G., Long, X.-L., Fan, L.-X., & Liao, Y.-M. (2019). A Study of Assessment Model of Oral English Imitation Reading in College Entrance Examination. In W. L. Li Q. Li W. (Ed.), Proceedings - 2018 11th International Congress on Image and Signal Processing, BioMedical Engineering and Informatics, CISP-BMEI 2018. Institute of Electrical and Electronics Engineers Inc. https://doi.org/10.1109/CISP-BMEI.2018.8633170 | 10. Educational-related but it is not about reading |
| Limsukhawat, S., Kaewyoun, S., Wongwatkit, C., & Wongta, J. (2016). A Development of Augmented Reality- supported Mobile Game Application based on Jolly Phonics Approach to Enhancing English Phonics Learning Performance of ESL Learners. In W. S.L., B. A.G., M. H., B. G., J. J., Y. J.-C., … Y. C. (Eds.), The 24th International Conference on Computers in Education (pp. 483–488). Department of Computer and Information Technology, Faculty of Industrial Education and Technology, King Mongkut’s University of Technology, Thonburi, Thailand: Asia-Pacific Society for Computers in Education. Retrieved from https://www.scopus.com/inward/record.uri?eid=2-s2.0-85018948298&partnerID=40&md5=f2ac247068212b5548a769de761d758d | 8. Training oriented to special population (other than dyslexia) |
| Lin, C. (2014). Learning English reading in a mobile-assisted extensive reading program. Computers & Education. https://doi.org/10.1016/j.compedu.2014.05.004 | 9. Training oriented to a second-language learning |
| Lin, C.-Y., Yu, W.-J., Chen, W.-J., Huang, C.-W., & Lin, C.-C. (2016). The Effect of Literacy Learning via Mobile Augmented Reality for the Students with ADHD and Reading Disabilities. (A. M. & S. C., Eds.). Department of Special Education, National University of Tainan, Tainan, Taiwan: Springer Verlag. https://doi.org/10.1007/978-3-319-40238-3_11 | 8. Training oriented to special population (other than dyslexia) |
| Lin, P.-H., Liu, T.-C., & Paas, F. (2017). 'Effects of spell checkers on English as a second language students’ incidental spelling learning: A cognitive load perspective’: Erratum. Reading and Writing: An Interdisciplinary Journal, 30(7), 1527–1528. https://doi.org/10.1007/s11145-017-9734-4 | 9. Training oriented to a second-language learning |
| Lin, Q., Yang, L., Jia, H., Duan, C., & Liu, Y. (2017). Revisiting reading rate with mobility: Rate-adaptive reading in COTS RFID systems. CoNEXT 2017 - Proceedings of the 2017 13th International Conference on Emerging Networking EXperiments and Technologies, 199–211. https://doi.org/10.1145/3143361.3143387 | 2. It is a reading-related technology but it is not a training (test, support…) |
| Lin, Q., Yang, L., Jia, H., Duan, C., & Liu, Y. (2017). Revisiting reading rate with mobility: Rate-adaptive reading in COTS RFID systems. CoNEXT 2017 - Proceedings of the 2017 13th International Conference on Emerging Networking EXperiments and Technologies, 199–211. https://doi.org/10.1145/3143361.3143387 | 5. Technology but it is not neither a training or reading-related |
| Liontou, T. (2018). DEVELOPING EFL READING COMPREHENSION COMPETENCE TO STUDENTS WITH LEARNING DIFFERENCES: EVIDENCE FROM A TECHNOLOGY-ENHANCED LEARNING ENVIRONMENT. In Chova, LG and Martinez, AL and Torres, IC (Ed.), 12TH INTERNATIONAL TECHNOLOGY, EDUCATION AND DEVELOPMENT CONFERENCE (INTED) (pp. 117–121). | 9. Training oriented to a second-language learning |
| Liontou, T. (2019). Foreign language learning for children with ADHD: evidence from a technology-enhanced learning environment. EUROPEAN JOURNAL OF SPECIAL NEEDS EDUCATION, 34(2, SI), 220–235. https://doi.org/10.1080/08856257.2019.1581403 | 9. Training oriented to a second-language learning |
| Littleton, K., Scanlon, E., & Sharples, M. (2011). Orchestrating Inquiry Learning. Orchestrating Inquiry Learning. Routledge, Taylor & Francis Group. https://doi.org/10.4324/9780203136195 | 10. Educational-related but it is not about reading |
| Liu, J. L., Jason McAnany, J., Wilensky, J. T., Aref, A. A., & Vajaranant, T. S. (2017). M&S Smart System Contrast Sensitivity Measurements Compared with Standard Visual Function Measurements in Primary Open-Angle Glaucoma Patients. Journal of Glaucoma, 26(6), 528–533. https://doi.org/10.1097/IJG.0000000000000659 | 10. Educational-related but it is not about reading |
| Liu, W. (2017). Research on Visual Design Based on Mobile Internet - Taking E-book APP as an Example. In Jing, W and Ning, X and Huiyu, Z (Ed.), PROCEEDINGS OF THE 7TH INTERNATIONAL CONFERENCE ON EDUCATION, MANAGEMENT, INFORMATION AND COMPUTER SCIENCE (ICEMC 2017) (Vol. 73, pp. 908–912). | 2. It is a reading-related technology but it is not a training (test, support…) |
| Lizunova V, I. (2019). The Market of Electronic Books in Russia: The Specifics of Production and Distribution. TEKST KNIGA KNIGOIZDANIE-TEXT BOOK PUBLISHING, 19, 106–125. https://doi.org/10.17223/23062061/19/8 | 11. Type of study excluded (review, non-primary references…) |
| Llema, C. F., & Vilela-Malabanan, C. M. (2019). Design and development of MLERWS: A user-centered mobile application for English reading and writing skills. In Y. A. (Ed.), Procedia Computer Science (Vol. 161, pp. 1002–1010). Elsevier B.V. https://doi.org/10.1016/j.procs.2019.11.210 | 9. Training oriented to a second-language learning |
| Llorens, A. C., Cerdán, R., & Vidal-Abarca, E. (2014). Adaptive formative feedback to improve strategic search decisions in task-oriented reading. Journal of Computer Assisted Learning, 30(3), 233–251. https://doi.org/10.1111/jcal.12050 | 2. It is a reading-related technology but it is not a training (test, support…) |
| Llorens, A. C., Vidal-Abarca, E., & Cerdán, R. (2016). Formative feedback to transfer self-regulation of task-oriented reading strategies. Journal of Computer Assisted Learning, 32(4), 314–331. https://doi.org/10.1111/jcal.12134 | 2. It is a reading-related technology but it is not a training (test, support…) |
| Llorens, A. C., Vidal-Abarca, E., Cerdán, R., & Ávila, V. (2015). Does formative feedback on search behaviour help students in answering comprehension questions from an available text? / ¿Ayuda la retroalimentación formativa sobre el comportamiento de búsqueda a contestar preguntas de comprensión en lecturas con texto d. Infancia y Aprendizaje, 38(4), 808–841. https://doi.org/10.1080/02103702.2015.1076269 | 4. Reading-related but it is not neither a training or technological |
| Logan, B. J. (2016). Comparing reading interventions for language arts students. Dissertation Abstracts International Section A: Humanities and Social Sciences. ProQuest Information & Learning, US. Retrieved from http://ezproxy.si.unav.es:2048/login?url=http://search.ebscohost.com/login.aspx?direct=true&AuthType=ip,url&db=psyh&AN=2016-99010-175&lang=es&site=eds-live&scope=site | 11. Type of study excluded (review, non-primary references…) |
| Loo, J. H. Y., BAMIOU, D. E. V. A., Campbell, N., & Luxon, L. M. (2010). Computer based auditory training (CBAT): benefits for children with language and reading related learning difficulties. Developmental Medicine & Child Neurology. Retrieved from http://ezproxy.si.unav.es:2048/login?url=http://search.ebscohost.com/login.aspx?direct=true&AuthType=ip,url&db=edswss&AN=000279723500013&lang=es&site=eds-live&scope=site | 11. Type of study excluded (review, non-primary references…) |
| Lorentz, P. (2015). The collaborative creation of video games: A marriage for love. New Media & Society, 17(6), 996–1002. https://doi.org/10.1177/1461444815574029 | 7. Technological reading training that do not fulfill methods requirements |
| Lousado, J. P., Costa, C., Oliveira, I. P., & Roberto, M. T. G. (2011). Audio folios management system for foreign language learning. In 6th Iberian Conference on Information Systems and Technologies (CISTI 2011) (pp. 1–5). Centro de Estudos Em Educação, Tecnologias e Saúde, Instituto Politécnico de Viseu, Campus Politécnico, 3504-510, Viseu, Portugal. Retrieved from https://www.scopus.com/inward/record.uri?eid=2-s2.0-80052486855&partnerID=40&md5=c3ca6c1b2bf58ba719e61a95c8a458c7 | 9. Training oriented to a second-language learning |
| Lovelace, T. S. (2008). The effects of explicit phonological awareness instruction on the prereading skills of preschool children at risk for reading failure: Comparing single and multiple skill instructional strategies. ProQuest Dissertations and Theses. ProQuest Information & Learning, US. Retrieved from http://search.proquest.com.ezp-prod1.hul.harvard.edu/docview/304484572?accountid=11311%5Cnhttp://sfx.hul.harvard.edu/hvd?url_ver=Z39.88-2004&rft_val_fmt=info:ofi/fmt:kev:mtx:dissertation&genre=dissertations+%26+theses&sid=ProQ:ProQuest+Dissertations+%26+T | 2. It is a reading-related technology but it is not a training (test, support…) |
| Lu, M. (2008). Effectiveness of vocabulary learning via mobile phone. Journal of Computer Assisted Learning, 24(May 2006), 515–525. https://doi.org/10.1111/j.1365-2729.2008.00289.x | 3. Technological training that is not focus on improving reading |
| Ludovico, L. A., Di Tore, P. A., Mangione, G. R., Di Tore, S., & Corona, F. (2015). Measuring the reading abilities of dyslexic children through a visual game. International Journal of Emerging Technologies in Learning, 10(7), 47–54. https://doi.org/10.3991/ijet.v10i7.4625 | 2. It is a reading-related technology but it is not a training (test, support…) |
| Ludtke, J., Froehlich, E., Jacobs, A. M., Hutzler, F., La1, Froehlich, E., Jacobs, A. M., Hutzler, F., Luedtke, J., Froehlich, E., Jacobsi, A. M., Hutzler, F., Lüdtke, J., Fröhlich, E., Jacobs, A. M., & Hutzler, F. (2019). The SLS-Berlin: Validation of a German Computer-Based Screening Test to Measure Reading Proficiency in Early and Late Adulthood. Frontiers in Psychology, 10(JULY), 1682. https://doi.org/10.3389/fpsyg.2019.01682 | 2. It is a reading-related technology but it is not a training (test, support…) |
| Lukes, D. (2016). Dyslexia friendly reader: Prototype, designs, and exploratory study. In IISA 2015 - 6th International Conference on Information, Intelligence, Systems and Applications. Research, Development and Policy, Dyslexia Action, United Kingdom: Institute of Electrical and Electronics Engineers Inc. https://doi.org/10.1109/IISA.2015.7388008 | 3. Technological training that is not focus on improving reading |
| Lukes, D., & Litsas, C. (2016). Building a phonics engine for automated text guidance. In IISA 2015 - 6th International Conference on Information, Intelligence, Systems and Applications. Research, Development and Policy, Dyslexia Action, United Kingdom: Institute of Electrical and Electronics Engineers Inc. https://doi.org/10.1109/IISA.2015.7388010 | 2. It is a reading-related technology but it is not a training (test, support…) |
| Lundälv, M., Derbring, S., Mühlenbock, K. H., Brännström, A., Farre, B., & Nordberg, L. (2014). Inclusive AAC: Multi-modal and multilingual language support for all. Technology and Disability, 26(2–3), 93–103. https://doi.org/10.3233/TAD-140407 | 7. Technological reading training that do not fulfill methods requirements |
| Luo, T., Lee, G.-L., & Molina, C. (2017). Incorporating istation into early childhood classrooms to improve reading comprehension. Journal of Information Technology Education: Research, 16(1), 247–266. https://www.scopus.com/inward/record.uri?eid=2-s2.0-85026211502&partnerID=40&md5=8cc8002a8356915daa3e56aeb7a75c46 | 7. Technological reading training that do not fulfill methods requirements |
| Luo, T., Lee, G.-L., & Molina, C. (2017). Incorporating istation into early childhood classrooms to improve reading comprehension. Journal of Information Technology Education: Research, 16(1), 247–266. https://www.scopus.com/inward/record.uri?eid=2-s2.0-85026211502&partnerID=40&md5=8cc8002a8356915daa3e56aeb7a75c46 | 7. Technological reading training that do not fulfill methods requirements |
| Luo, T., Lee, G.-L., & Molina, C. (2017). Incorporating istation into early childhood classrooms to improve reading comprehension. Journal of Information Technology Education: Research, 16(1), 247–266. Retrieved from https://www.scopus.com/inward/record.uri?eid=2-s2.0-85026211502&partnerID=40&md5=8cc8002a8356915daa3e56aeb7a75c46 | 7. Technological reading training that do not fulfill methods requirements |
| Luthra, V., & Ghosh, S. (2015). Understanding, evaluating and analyzing touch screen gestures for visually impaired users in mobile environment. (S. C., A. M., & S. C., Eds.), Lecture Notes in Computer Science (Including Subseries Lecture Notes in Artificial Intelligence and Lecture Notes in Bioinformatics). Samsung Research and Development Institute-Bangalore, Bangalore, India: Springer Verlag. https://doi.org/10.1007/978-3-319-20681-3_3 | 8. Training oriented to special population (other than dyslexia) |
| Lyddon, P. A. (2011). The Efficacy of Corrective Feedback and Textual Enhancement in Promoting the Acquisition of Grammatical Redundancies. Modern Language Journal, 95(SUPPL. 1), 104–129. https://doi.org/10.1111/j.1540-4781.2011.01272.x | 1.Non technological reading training |
| Lytridis, C., & Tsinakos, A. (2014). Using a Commercial Mobile Application as a Mobile Learning Platform. (K. M., S. M., & B. Y., Eds.), Mobile As Mainstream-Towards Future Challenges in Mobile Learning, Mlearn 2014. Department of Computer and Informatics Engineering, Eastern Macedonia and Thrace Institute of Technology, Ag. Loukas, Kavala, Greece: Springer Verlag. Retrieved from https://www.scopus.com/inward/record.uri?eid=2-s2.0-84908516678&partnerID=40&md5=7d86207f87f27627781af945788a54da | 10. Educational-related but it is not about reading |
| M.E., A., J., U., & D., G. (Eds.). (2017). 19th International Conference on Interactive Collaborative Learning, ICL 2016. In Advances in Intelligent Systems and Computing (Vol. 545, pp. 1–638). Springer Verlag. https://www.scopus.com/inward/record.uri?eid=2-s2.0-85010022794&partnerID=40&md5=bebfc68459ba7de4661815f3a39de7db | 11. Type of study excluded (review, non-primary references…) |
| Ma, W., Adesope, O. O., Nesbit, J. C., & Liu, Q. (2014). Intelligent tutoring systems and learning outcomes: A meta-analysis. Journal of Educational Psychology, 106(4), 901–918. https://doi.org/10.1037/a0037123 | 5. Technology but it is not neither a training or reading-related |
| Maaß, E. E., Hahlweg, K., Heinrichs, N., Kuschel, A., Naumann, S., Bertram, H., … Döpfner, M. (2010). Sozioökonomischer status, mütterliches erziehungsverhalten, erhöhter medienkonsum und die sprach- und rechenfertigkeiten von kindergartenkindern. Psychologie in Erziehung Und Unterricht, 57(1), 46–61. https://doi.org/10.2378/peu2010.art04d | 5. Technology but it is not neither a training or reading-related |
| Macias, C., Panch, T., Hicks, Y. M., Scolnick, J. S., Weene, D. L., Öngür, D., & Cohen, B. M. (2015). Using Smartphone Apps to Promote Psychiatric and Physical Well-Being. Psychiatric Quarterly, 86(4), 505–519. https://doi.org/10.1007/s11126-015-9337-7 | 5. Technology but it is not neither a training or reading-related |
| Madeira, J., Silva, C., Marcelino, L., & Ferreira, P. (2015). Assistive Mobile Applications for Dyslexia. Procedia Computer Science, 64(Conference on ENTERprise Information Systems/International Conference on Project MANagement/Conference on Health and Social Care Information Systems and Technologies, CENTERIS/ProjMAN / HCist 2015 October 7-9, 2015), 417–424. https://doi.org/10.1016/j.procs.2015.08.535 | 7. Technological reading training that do not fulfill methods requirements |
| Magliano, J. P., Higgs, K., & Millis, K. (2018). Deep comprehension of text revealed by talking and writing while reading. In Deep Comprehension: Multi-Disciplinary Approaches to Understanding, Enhancing, and Measuring Comprehension. Taylor and Francis. https://doi.org/10.4324/9781315109503 | 11. Type of study excluded (review, non-primary references…) |
| Magliano, J. P., Millis, K. K., Levinstein, I., & Boonthum, C. (2011). Assessing comprehension during reading with the Reading Strategy Assessment Tool (RSAT). Metacognition and Learning. https://doi.org/10.1007/s11409-010-9064-2 | 2. It is a reading-related technology but it is not a training (test, support…) |
| Magliano, J. P., Ray, M., & Millis, K. K. (2017). THE READING STRATEGY ASSESSMENT TOOL A Computer-Based Approach for Evaluating Comprehension Processes during Reading. In Crossley, SA and McNamara, DS (Ed.), ADAPTIVE EDUCATIONAL TECHNOLOGIES FOR LITERACY INSTRUCTION (pp. 282–287). | 2. It is a reading-related technology but it is not a training (test, support…) |
| Mahesh, G., Jayahari, K. R., & Bijlani, K. (2016). A smart phone integrated smart classroom. In A.-B. K. & A.-B. K. (Eds.), International Conference on Next Generation Mobile Applications, Services, and Technologies (pp. 88–93). Amrita E-Learning Research Lab (AERL), Amrita School of Engineering, Amrita University, Amritapuri, India: IEEE Computer Society. https://doi.org/10.1109/NGMAST.2016.31 | 10. Educational-related but it is not about reading |
| Mahmood, A., ul Waheed, S., Anjum, M. A. I., & Majeed, R. (2013). Computer Assisted Language Learning: An Instrument of Change for Boosting Motivation Level among the Students of Graduation in Pakistan. Language in India, 13(7), 159–170. Retrieved from http://search.proquest.com/docview/1650525792?accountid=14548%5Cnhttp://metadata.lib.hku.hk/hku?url_ver=Z39.88-2004&rft_val_fmt=info:ofi/fmt:kev:mtx:journal&genre=article&sid=ProQ:ProQ:llba&atitle=Computer+Assisted+Language+Learning:+An+Instrument+of+Cha | 5. Technology but it is not neither a training or reading-related |
| Mahmoudian, N. (2017). A CONTENT ANALYSIS OF VOCABULARY LEARNING WEBSITES WITH A VIEW TOWARDS MATERIALS DEVELOPEMENT. MODERN JOURNAL OF LANGUAGE TEACHING METHODS, 7(5), 88–97. | 9. Training oriented to a second-language learning |
| Mai, P., & Kuo, P. (2012). Research on the usability of digital magazine applications. In 8th International Conference on Information Science and Digital Content Technology (ICIDT) (Vol. 2, pp. 430–433). Master’s Program of Digital Content and Technologies, National Cheng-Chi University, Taipei, Taiwan. Retrieved from http://ieeexplore.ieee.org/xpls/abs_all.jsp?arnumber=6269307 | 5. Technology but it is not neither a training or reading-related |
| Maier, U. (2014). Computer-based, formative assessment in primary and secondary education - A literature review on development, implementation and effects . Unterrichtswissenschaft, 42(1), 69–86. Retrieved from https://www.scopus.com/inward/record.uri?eid=2-s2.0-84904439099&partnerID=40&md5=3c24673149ef8df68e61ae36515b47d8 | 11. Type of study excluded (review, non-primary references…) |
| Maier, U., Randler, C., & Wolf, N. (2016). Effects of Computer-Based, Formative Tests with Diverse Feedback Formats on Student Performance in Science Classes. Zeitschrift Fur Padagogik, 62(2), 241–262. | 11. Type of study excluded (review, non-primary references…) |
| Maknouz, D. (2016). Digital natives to the test: Cognitive skills and abilities of networked students . Mondo Digitale, 15(62). Retrieved from https://www.scopus.com/inward/record.uri?eid=2-s2.0-84979462411&partnerID=40&md5=257a95be4192be11d63cac3614740b46 | 11. Type of study excluded (review, non-primary references…) |
| Mallett, L., & Orr, T. J. (2008). Working in the Classroom - A Vision of Miner Training in the 21st Century. In Saydam, S (Ed.), First International Future Mining Conference and Exhibition 2008, Proceedings (pp. 83–89). Retrieved from wos:000269969900011%5Cnhttp://www2a.cdc.gov/nioshtic-2/BuildQyr.asp?s1=virtual+reality&f1=*&Adv=0&terms=1&PageNo=1&RecNo=6&View=f& | 10. Educational-related but it is not about reading |
| Malstädt, N., Hasselhorn, M., & Lehmann, M. (2012). Free recall behaviour in children with and without spelling impairment: The impact of working memory subcapacities. Dyslexia, 18(4), 187–198. https://doi.org/10.1002/dys.1446 | 7. Technological reading training that do not fulfill methods requirements |
| Mandaleeka, N. G., & Ali, M. A. (2011). Human Face of Technology & Its Deployment: A Case of Adult Literacy Program. In 2011 IEEE Global Humanitarian Technology Conference (pp. 487–492). Business Systems and Cybernetics Centre, Tata Consultancy Services, Hyderabad, India. https://doi.org/10.1109/GHTC.2011.53 | 7. Technological reading training that do not fulfill methods requirements |
| Mansor, W., Khuan, L. Y., Che Wan Fadzal, C. W. N. F., Mohammad, N., & Amirin, S. (2016). Development of computer-based assessment for brain electrophysiology technique of dyslexic children. In ISCAIE 2016 - 2016 IEEE Symposium on Computer Applications and Industrial Electronics (pp. 79–83). Medical Engineering Technology Section, Universiti Kuala Lumpur-British Malaysian Institute, Gombak, Selangor, Malaysia: Institute of Electrical and Electronics Engineers Inc. https://doi.org/10.1109/ISCAIE.2016.7575041 | 2. It is a reading-related technology but it is not a training (test, support…) |
| Mares-Rosas, L. A., Vargas-Huitron, J., Martinez-Juarez, J. A., Ariza-Mercado, E. D., Rivera-Reyes, R. E., Acebedo-Calderon, O., Tocohua Rojas, R., Pedraza Cruz, F. I., & Veliz Martinez, J. C. (2018). PROPOSAL OF A SOFTWARE ARCHITECTURE IN THE CLOUD FOR SERIOUS GAMES APPLY IN LEARNING DISORDERS. In Chova, LG and Martinez, AL and Torres, IC (Ed.), 12TH INTERNATIONAL TECHNOLOGY, EDUCATION AND DEVELOPMENT CONFERENCE (INTED) (pp. 5052–5057). | 3. Technological training that is not focus on improving reading |
| Margelisch, K., & Perrig, W. J. (2015). Impacts of a word-picture training on reading in youth with mixed intellectual disabilities. A waiting-list control group comparison. https://doi.org/10.7892/boris.63563 | 8. Training oriented to special population (other than dyslexia) |
| Marhoubi, A. H., Saravi, S., & Edirisinghe, E. A. (2015). The Application of Machine Learning in Multi Sensor Data Fusion for Activity Recognition in Mobile Device Space. In D. A.K. & D. N.K. (Eds.), Image Sensing Technologies: Materials, Devices, Systems, and Applications Ii (Vol. 9481, pp. 1–9). Computer Science Department, Loughborough University, United Kingdom: SPIE. https://doi.org/94810g\r10.1117/12.2177115 | 5. Technology but it is not neither a training or reading-related |
| Maria Jimenez-Porta, A., & Diez-Martinez, E. (2018). Impact of videogames on reading fluency in children with and without dyslexia. The case of Minecraft. REVISTA LATINOAMERICANA DE TECNOLOGIA EDUCATIVA-RELATEC, 17(1), 77–90. https://doi.org/10.17398/1695-288X.17.1.77 | 7. Technological reading training that do not fulfill methods requirements |
| Marques, C. G. C., Manso, A., Ferreira, A. P., & Morgado, F. (2017). Using Mobile Technologies in Education: A New Pedagogical Approach to Promote Reading Literacy. International Journal of Technology and Human Interaction ((IJTHI)), 13(4), 77–90. https://doi.org/10.4018/IJTHI.2017100106 | 7. Technological reading training that do not fulfill methods requirements |
| Marques, C. G. C., Manso, A., Ferreira, A. P., Morgado, F., Cardoso Marques, C. G., Manso, A., Ferreira, A. P., & Morgado, F. (2017). Using mobile technologies in education: A new pedagogical approach to promote reading literacy. INTERNATIONAL JOURNAL OF TECHNOLOGY AND HUMAN INTERACTION, 13(4), 77–90. https://doi.org/10.4018/IJTHI.2017100106 | 10. Educational-related but it is not about reading |
| Marques, C. G., Manso, A., Dias, P., Ferreira, A. P., & Morgado, F. (2016). Information system to promote reading literacy-Letrinhas. In R. A., R. L.P., C. M.P., G. R., & S. O.S. (Eds.), Iberian Conference on Information Systems and Technologies, CISTI (Vol. 2016-July). Centre for Public Policy and Administration (CAPP), Instituto Politécnico de Tomar, Tomar, Portugal: IEEE Computer Society. https://doi.org/10.1109/CISTI.2016.7521470 | 7. Technological reading training that do not fulfill methods requirements |
| Martínez, T., Vidal-Abarca, E., Gil, L., & Gilabert, R. (2009). On-line assessment of comprehension processes. The Spanish Journal of Psychology, 12(1), 308–319. https://doi.org/10.1017/S1138741600001700 | 2. It is a reading-related technology but it is not a training (test, support…) |
| Martins, V. F., Lima, T., Sampaio, P. N. M., & De Paiva, M. (2017). Mobile application to support dyslexia diagnostic and reading practice. In Proceedings of IEEE/ACS International Conference on Computer Systems and Applications, AICCSA. https://doi.org/10.1109/AICCSA.2016.7945710 |  |
| Maslamani, J. A., Windeatt, S., Olivier, P., Heslop, P., Kharrufa, A., Shearer, J., & Balaam, M. (2012). Collaborative Strategic Reading on Multi-Touch and Multi-User Digital Tabletop Displays. CALL: Using, Learning, Knowing, EUROCALL Conference, Gothenburg, Sweden, 22-25 August 2012, Proceedings. Research-publishing. net. https://doi.org/10.14705/rpnet.2012.000054 | 9. Training oriented to a second-language learning |
| Mayer, S. A. (2010). The role of learners’ questions and instructors’ voice and gestures in the comprehension of lessons. Dissertation Abstracts International: Section B: The Sciences and Engineering. ProQuest Information & Learning, US. Retrieved from http://ezproxy.si.unav.es:2048/login?url=http://search.ebscohost.com/login.aspx?direct=true&AuthType=ip,url&db=psyh&AN=2010-99020-057&lang=es&site=eds-live&scope=site | 10. Educational-related but it is not about reading |
| Mayora, C., Nieves, I., & Ojeda, V. (2014). An in-house prototype for the implementation of computer-based extensive reading in a limited-resource school. The Reading Matrix, 14(2), 78–95. Retrieved from http://search.ebscohost.com/login.aspx?direct=true&AuthType=ip,url,cookie,uid&db=eue&AN=99810582&site=ehost-live&scope=site | 9. Training oriented to a second-language learning |
| Mazlan, N. H., Daud, S. M., & Ayob, A. F. (2010). Effectiveness of assistive computer technology (ACT) for enhancing basic language skills among students with hearing disabilities. Pertanika Journal of Social Science and Humanities, 18(1), 141–156. Retrieved from http://ezproxy.si.unav.es:2048/login?url=http://search.ebscohost.com/login.aspx?direct=true&AuthType=ip,url&db=edb&AN=50837545&lang=es&site=eds-live&scope=site | 8. Training oriented to special population (other than dyslexia) |
| McTigue, E. M., Soiheim, O. J., Zimmer, W. K., & Uppstad, P. H. (2020). Critically Reviewing GraphoGame Across the World: Recommendations and Cautions for Research and Implementation of Computer-Assisted Instruction for Word-Reading Acquisition. READING RESEARCH QUARTERLY, 55(1), 45–73. https://doi.org/10.1002/rrq.256 | 11. Type of study excluded (review, non-primary references…) |
| Mehringer, H., Fraga-González, G., Pleisch, G., Röthlisberger, M., Aepli, F., Keller, V., Karipidis, I. I., & Brem, S. (2020). (Swiss) GraphoLearn: an app-based tool to support beginning readers. Research and Practice in Technology Enhanced Learning, 15(1). https://doi.org/10.1186/s41039-020-0125-0 | 7. Technological reading training that do not fulfill methods requirements |
| Mele, M. L., & Federici, S. (2012). Gaze and eye-tracking solutions for psychological research. Cognitive Processing, 13(1 SUPPL), 261. https://doi.org/10.1007/s10339-012-0499-z | 5. Technology but it is not neither a training or reading-related |
| Melhi, A. A. (2014). Effects on and Predictability of Computer-mediated Glosses in Reading Comprehension of EFL College Students. Reading Matrix: An International Online Journal, 14(2), 65–77. Retrieved from http://ezproxy.si.unav.es:2048/login?url=http://search.ebscohost.com/login.aspx?direct=true&AuthType=ip,url&db=eric&AN=EJ1046927&lang=es&site=eds-live&scope=site | 9. Training oriented to a second-language learning |
| Mersand, S. (2015). Lexia Reading Core5. Tech & Learning, 35(7), 14. Retrieved from http://ezproxy.georgetowncollege.edu:2048/login?URL=http://search.ebscohost.com/login.aspx?direct=true&db=eue&AN=102824620 | 11. Type of study excluded (review, non-primary references…) |
| Messer, D., & Nash, G. (2018). An evaluation of the effectiveness of a computer-assisted reading intervention. JOURNAL OF RESEARCH IN READING, 41(1), 140–158. https://doi.org/10.1111/1467-9817.12107 | 12. Other: No access granted, same data… |
| Mestrovic, D., & Marot, N. J. (2017). E-BOOK READING APPLICATIONS - INNOVATIVE TECHNOLOGY AS A RESPONSE TO GROWING CONSUMER PREFERENCES AND ITS IMPLICATIONS FOR TOURISM. In Markovic, S and Jurdana, DS (Ed.), 4TH INTERNATIONAL SCIENTIFIC CONFERENCE: TOSEE - TOURISM IN SOUTHERN AND EASTERN EUROPE 2017 (Vol. 4, pp. 349–363). https://doi.org/10.20867/tosee.04.12 | 2. It is a reading-related technology but it is not a training (test, support…) |
| Mican, D., & Tomai, N. (2015). Study on Students Mobile Learning Acceptance. In Boja, C and Doinea, M and Ciurea, C and Pocatilu, P and Batagan, L and Ion, A and Diaconita, V and Andreica, M and Delcea, C and Zamfiroiu, A and Zurini, M and Popescu, O (Ed.), Proceedings of the 14Th International Conference on Informatics in Economy (Ie 2015): Education, Research & Business Technologies (pp. 122–127). | 3. Technological training that is not focus on improving reading |
| Michaelsen, E., & Pihl, J. (2012). Enhancing Children’S Reading Engagement in the Digital Age: Cartoons, Action Stories and Videogames. In Chova, LG and Torres, IC and Martinez, AL (Ed.), Edulearn12: 4Th International Conference on Education and New Learning Technologies (pp. 1809–1818). | 2. It is a reading-related technology but it is not a training (test, support…) |
| Michaelsen, E., & Pihl, J. (2012). Enhancing Children’S Reading Engagement in the Digital Age: Cartoons, Action Stories and Videogames. In Chova, LG and Torres, IC and Martinez, AL (Ed.), Edulearn12: 4Th International Conference on Education and New Learning Technologies (pp. 1809–1818). | 3. Technological training that is not focus on improving reading |
| MIHAIL-VADUVA, D. (2018). Enriching Curricula with Mobile Solutions. Informatică Economică VO  - 22, 3, 33. https://doi.org/10.12948/issn14531305/22.3.2018.04 | 3. Technological training that is not focus on improving reading |
| Mohammed, W. A., & Husni, H. (2017). Reading apps for children: Readability from the design perspective. In L. C. K. Nifa F.A.A. Hussain A. (Ed.), AIP Conference Proceedings (Vol. 1891). American Institute of Physics Inc. https://doi.org/10.1063/1.5005428 | 2. It is a reading-related technology but it is not a training (test, support…) |
| Mohammed, W. A., & Husni, H. (2017). Reading Apps for Children: Readability from the Design Perspective. In Nifa, FAA and Lin, CK and Hussain, A (Ed.), 2ND INTERNATIONAL CONFERENCE ON APPLIED SCIENCE AND TECHNOLOGY 2017 (ICAST’17) (Vol. 1891). https://doi.org/10.1063/1.5005428 | 2. It is a reading-related technology but it is not a training (test, support…) |
| Mokgonyane, T. B., Sefara, T. J., Manamela, P. J., Manamela, M. J., & Modipa, T. I. (2017). Development of a speech-enabled basic arithmetic m-learning application for foundation phase learners. In C. D.R. (Ed.), 2017 IEEE AFRICON: Science, Technology and Innovation for Africa, AFRICON 2017 (pp. 794–799). Institute of Electrical and Electronics Engineers Inc. https://doi.org/10.1109/AFRCON.2017.8095584 | 3. Technological training that is not focus on improving reading |
| Mokgonyane, T. B., Sefara, T. J., Manamela, P. J., Manamela, M. J., & Modipa, T. I. (2017). Development of a speech-enabled basic arithmetic m-learning application for foundation phase learners. In C. D.R. (Ed.), 2017 IEEE AFRICON: Science, Technology and Innovation for Africa, AFRICON 2017 (pp. 794–799). Institute of Electrical and Electronics Engineers Inc. https://doi.org/10.1109/AFRCON.2017.8095584 | 3. Technological training that is not focus on improving reading |
| Molnar, G., & Szuts, Z. (2014). Advanced mobile communication and media devices and applications in the base of higher education. In S. A. (Ed.), SISY 2014 - IEEE 12th International Symposium on Intelligent Systems and Informatics, Proceedings (pp. 169–174). Budapest University of Technology and Economics, Department of Technical Education, Budapest, Hungary: Institute of Electrical and Electronics Engineers Inc. https://doi.org/10.1109/SISY.2014.6923580 | 5. Technology but it is not neither a training or reading-related |
| Monahan, B. (2010). The Use of Technology to Improve the Reading Skills of Second Language Learners in Grades K-2. NAAAS & Affiliates Conference Monographs, 619. Retrieved from http://ezproxy.si.unav.es:2048/login?url=http://search.ebscohost.com/login.aspx?direct=true&AuthType=ip,url&db=edb&AN=61059929&lang=es&site=eds-live&scope=site | 9. Training oriented to a second-language learning |
| Montalvo-Castro, J. A., & Martos-Castaneda, N. (2019). Design and evaluation of a new digital format for reading illustrated tales: Correpalabras. OCNOS-REVISTA DE ESTUDIOS SOBRE LA LECTURA, 18(3), 29–37. https://doi.org/10.18239/ocnos_2019.18.3.2008 | 2. It is a reading-related technology but it is not a training (test, support…) |
| Monticelli, C., De Oliveira Heidrich, R., Rodrigues, R., Cappelatti, E., Goulart, R., Oliveira, R., & Velho, E. (2018). Text vocalizing desktop scanner for visually impaired people. Communications in Computer and Information Science, 851, 62–67. https://doi.org/10.1007/978-3-319-92279-9_8 | 8. Training oriented to special population (other than dyslexia) |
| Moore, K. L. (2012). Effectiveness of A Computer-Based Program for Improving The Reading Performance of Deaf Students. ProQuest LLC. ProQuest LLC, US. Retrieved from http://ezproxy.si.unav.es:2048/login?url=http://search.ebscohost.com/login.aspx?direct=true&AuthType=ip,url&db=psyh&AN=2013-99111-107&lang=es&site=eds-live&scope=site | 8. Training oriented to special population (other than dyslexia) |
| Mori, K., Ballagas, R., Revelle, G., Raffle, H., Horii, H., & Spasojevic, M. (2011). Interactive rich reading. In Proceedings of the 19th ACM international conference on Multimedia - MM ’11 (p. 825). Nokia Research Center, 955 Page Mill Rd, Palo Alto CA 94306, United States. https://doi.org/10.1145/2072298.2072478 | 7. Technological reading training that do not fulfill methods requirements |
| Moser, A., & Hlavacs, H. (2018). Learning Supported by Story Templates. In Ciussi, M (Ed.), PROCEEDINGS OF THE 12TH EUROPEAN CONFERENCE ON GAMES BASED LEARNING (ECGBL 2018) (pp. 441–446). | 5. Technology but it is not neither a training or reading-related |
| Moser, G. P., Morrison, T. G., & Wilcox, B. (2017). Supporting Fourth-Grade Students’ Word Identification Using Application Software. Reading Psychology, 38(4), 349–368. https://doi.org/10.1080/02702711.2016.1278414 | 12. Other: No access granted, same data… |
| MR, G., BC, M., SJ, W., Yonkman, J., AJ, S., Shirley, B., … Burdea, G. C. (2010). In-home virtual reality videogame telerehabilitation in adolescents with hemiplegic cerebral palsy. Archives of Physical Medicine & Rehabilitation, 91(1), 1–8. https://doi.org/10.1016/j.apmr.2009.08.153 | 8. Training oriented to special population (other than dyslexia) |
| Mudanyali, O., Dimitrov, S., Sikora, U., Padmanabhan, S., Navruz, I., & Ozcan, A. (2012). Integrated rapid-diagnostic-test reader platform on a cellphone. Lab on a Chip, 12(15), 2678. https://doi.org/10.1039/c2lc40235a | 2. It is a reading-related technology but it is not a training (test, support…) |
| Muharlisiani, L. T., Kukuh, A., & Azizah, S. (2018). DESIGNING COMPUTER-BASED EXERCISES USING WEBLOG, HOT POTATOES SOFTWARE AND SKYPE MESSENGERS IN CREATING IDEAS TO FACILITATE INDEPENDENCE LEARNING OF READING COMPREHENSION. Eltin Journal VO  - 6, 1, 11. https://doi.org/10.22460/ej.v6i1p11-21.1029 | 3. Technological training that is not focus on improving reading |
| Mukhitdinova, K., Asilova, G., Salisheva, Z., & Rakhmatullaeva, M. (2019). Current issues of creating educational material for intensive teaching to the Uzbek language of foreigners. International Journal of Engineering and Advanced Technology, 9(1), 5224–5226. https://doi.org/10.35940/ijeat.A2945.109119 | 9. Training oriented to a second-language learning |
| Mun, S. P., Husin, M. H., Singh, M. M., & Malim, N. H. A. H. (2018). N-LibSys: Library system using NFC technology. Lecture Notes in Electrical Engineering, 425, 22–30. https://doi.org/10.1007/978-981-10-5281-1_3 | 5. Technology but it is not neither a training or reading-related |
| Murphy, C., & Schochat, E. (2013). Effects of different types of auditory temporal training on language skills: a systematic review. Clinics, 68(10), 1364–1370. https://doi.org/10.6061/clinics/2013(10)12 | 11. Type of study excluded (review, non-primary references…) |
| Mursidi, A., Murdani, E., Sumarli, Buyung, & Rosmaiyadi. (2018). Role of WhatsApp application in building the interests of students literacy. ACM International Conference Proceeding Series, 1–4. https://doi.org/10.1145/3268808.3268815 | 2. It is a reading-related technology but it is not a training (test, support…) |
| Murukannaiah, P. K., & Singh, M. P. (2015). Platys. ACM Transactions on Software Engineering and Methodology, 24(3), 1–32. https://doi.org/10.1145/2729976 | 3. Technological training that is not focus on improving reading |
| Mustapa, A.-M., Rahman, Z. A., Ghani, M. Z. A., Saad, M. F. M., & Mohamed, F. A. (2018). Qiraahbot’s prototype development for an extensive reading activity. International Journal of Civil Engineering and Technology, 9(9), 1494–1503. https://www.scopus.com/inward/record.uri?eid=2-s2.0-85054766052&partnerID=40&md5=f114536ef28e4e6cd98bb59e99d9b9c0 | 3. Technological training that is not focus on improving reading |
| Myrberg, C. (2017). Why doesn’t everyone love reading e-books? Insights: The UKSG Journal, 30(3), 115–125. https://doi.org/10.1629/uksg.386 | 2. It is a reading-related technology but it is not a training (test, support…) |
| Nanjundaswamy, M., Prabhu, P., & Kittur, R. (2017). Computer-Based Auditory Training Programs for Children with Hearing Impairment – A Scoping Review. International Archives of Otorhinolaryngology. Department of Electronics, All India Institute of Speech and Hearing Ringgold Standard Institution, Mysore, Karnataka, India: Georg Thieme Verlag. https://doi.org/10.1055/s-0037-1602797 | 8. Training oriented to special population (other than dyslexia) |
| Narin, B. (2018). Reading Digital News: Hypertextual Usage Habits and Learning Practices Among U. S. Communication Undergraduates. CONNECTIST-ISTANBUL UNIVERSITY JOURNAL OF COMMUNICATION SCIENCES, 55, 143–169. https://doi.org/10.26650/CONNECTIST497463 | 5. Technology but it is not neither a training or reading-related |
| Naumann, J., & Goldhammer, F. (2017). Time-on-task effects in digital reading are non-linear and moderated by persons’ skills and tasks’ demands. LEARNING AND INDIVIDUAL DIFFERENCES, 53, 1–16. https://doi.org/10.1016/j.lindif.2016.10.002 | 2. It is a reading-related technology but it is not a training (test, support…) |
| Naumann, J., & Sälzer, C. (2017). Digital reading proficiency in german 15-year olds: evidence from PISA 2012 [Kompetenzen 15-jähriger Schülerinnen und Schüler beim Lesen digitaler Texte in Deutschland: Befunde aus PISA 2012]. Zeitschrift Fur Erziehungswissenschaft, 20(4), 585–603. https://doi.org/10.1007/s11618-017-0758-y | 4. Reading-related but it is not neither a training or technological |
| Naumann, J., & Sälzer, C. (2017). Digital reading proficiency in german 15-year olds: evidence from PISA 2012 [Kompetenzen 15-jähriger Schülerinnen und Schüler beim Lesen digitaler Texte in Deutschland: Befunde aus PISA 2012]. Zeitschrift Fur Erziehungswissenschaft, 20(4), 585–603. https://doi.org/10.1007/s11618-017-0758-y | 4. Reading-related but it is not neither a training or technological |
| Nikolaeva, Y. S., & Pak, N. I. (2017). Newest web-technologies for studying and diagnosing individual abilities of learners. Journal of Social Studies Education Research, 8(2), 130–144. https://doi.org/10.17499/jsser.360869 | 3. Technological training that is not focus on improving reading |
| Nip, T., Gunter, E. L., Herman, G. L., Morphew, J. W., & West, M. (2018). Using a computer-based testing facility to improve student learning in a programming languages and compilers course. SIGCSE 2018 - Proceedings of the 49th ACM Technical Symposium on Computer Science Education, 2018-Janua, 568–573. https://doi.org/10.1145/3159450.3159500 | 1.Non technological reading training |
| Nip, T., Gunter, E. L., Herman, G. L., Morphew, J. W., & West, M. (2018). Using a computer-based testing facility to improve student learning in a programming languages and compilers course. SIGCSE 2018 - Proceedings of the 49th ACM Technical Symposium on Computer Science Education, 2018-Janua, 568–573. https://doi.org/10.1145/3159450.3159500 | 1.Non technological reading training |
| Not found | 2. It is a reading-related technology but it is not a training (test, support…) |
| Nootens, P., Morin, M.-F., Alamargot, D., Goncalves, C., Venet, M., & Labrecque, A.-M. (2019). Differences in Attitudes Toward Reading: A Survey of Pupils in Grades 5 to 8. FRONTIERS IN PSYCHOLOGY, 9, 2773. https://doi.org/10.3389/fpsyg.2018.02773 | 10. Educational-related but it is not about reading |
| O’Brien, E., Jacquouton, B., Moineau, A., & Campbell, A. G. (2019). Wikipedia in Virtual Reality and HowText-based Media can be Explore in Virtual Reality. ACM International Conference Proceeding Series. https://doi.org/10.1145/3358331.3358401 | 10. Educational-related but it is not about reading |
| OECD. (2014). PISA 2012 Technical Report. OECD Report. OECD Publishing. https://doi.org/10.1787/9789264190511-en | 10. Educational-related but it is not about reading |
| Omar, S., & Bidin, A. (2015). The Impact of Multimedia Graphic and Text with Autistic Learners in Reading. Universal Journal of Educational Research, 3(12), 989–996. https://doi.org/10.13189/ujer.2015.031206 | 8. Training oriented to special population (other than dyslexia) |
| Ono, Y., Ishihara, M., & Yamashiro, M. (2012). Mobile-based shadowing materials in foreign language teaching. 1st IEEE Global Conference on Consumer Electronics 2012, GCCE 2012, 90–93. https://doi.org/10.1109/GCCE.2012.6379976 | 9. Training oriented to a second-language learning |
| Orji, F., Deters, R., Greer, J., & Vassileva, J. (2018). ClassApp: A Motivational Course-level App. In Chakrabarti, S and Saha, HN (Ed.), 2018 IEEE 9TH ANNUAL INFORMATION TECHNOLOGY, ELECTRONICS AND MOBILE COMMUNICATION CONFERENCE (IEMCON) (pp. 49–53). | 3. Technological training that is not focus on improving reading |
| Osakwe, J., Dlodlo, N., & Jere, N. (2017). Where learners’ and teachers’ perceptions on mobile learning meet: A case of Namibian secondary schools in the Khomas region. TECHNOLOGY IN SOCIETY, 49, 16–30. https://doi.org/10.1016/j.techsoc.2016.12.004 | 2. It is a reading-related technology but it is not a training (test, support…) |
| Osifo, A. (2019). Improving collaboration in blended learning environments through differentiated activities and mobile-assisted language learning tools. In I. P. O. G. S. I. A. Ravesteijn P. Rodrigues L. (Ed.), Proceedings of the 15th International Conference on Mobile Learning 2019, ML 2019 (pp. 3–10). IADIS Press. https://www.scopus.com/inward/record.uri?eid=2-s2.0-85065881577&partnerID=40&md5=910e4645f2bb49fdecb6147562f4e11f | 11. Type of study excluded (review, non-primary references…) |
| Ostiz-Blanco, M., Lallier, M., Grau, S., Rello, L., Bigham, J. P., & Carreiras, M. (2018). Jellys: Towards a Videogame that Trains Rhythm and Visual Attention for Dyslexia. ASSETS’18: PROCEEDINGS OF THE 20TH INTERNATIONAL ACM SIGACCESS CONFERENCE ON COMPUTERS AND ACCESSIBILITY, 447–449. https://doi.org/10.1145/3234695.3241028 | 7. Technological reading training that do not fulfill methods requirements |
| Ostiz-Blanco, M., Lallier, M., Grau, S., Rello, L., Bigham, J. P., & Carreiras, M. (2018). Jellys: Towards a Videogame that Trains Rhythm and Visual Attention for Dyslexia. ASSETS’18: PROCEEDINGS OF THE 20TH INTERNATIONAL ACM SIGACCESS CONFERENCE ON COMPUTERS AND ACCESSIBILITY, 447–449. https://doi.org/10.1145/3234695.3241028 | 11. Type of study excluded (review, non-primary references…) |
| Pablo, A. Q.-S., Espinoza, V., & Eva, U. (2017). Application of cloud, knowledge and innovation model for improving linguistic in writing skill. In R. L.P., R. A., A. B., C. C., & C. M.P. (Eds.), Information Systems and Technologies (CISTI), 2017 12th Iberian Conference on. Universidad Técnica Particular de Loja, Department of Electronics and Computer Science, Grupo Innovación Tecnológica Educativa, Loja - Quito, Ecuador: IEEE Computer Society. https://doi.org/10.23919/CISTI.2017.7975903 | 3. Technological training that is not focus on improving reading |
| Paciga, K. A. (2015). Their teacher can’t be an app: Preschoolers’ listening comprehension of digital storybooks. Journal of Early Childhood Literacy, 15(4), 473–509. https://doi.org/10.1177/1468798414552510 | 7. Technological reading training that do not fulfill methods requirements |
| Pae, H. K., Kim, S.-A., Mano, Q. R., & Kwon, Y.-J. Y.-J. (2017). Sublexical and lexical processing of the English orthography among native speakers of Chinese and Korean. Reading and Writing, 30(1), 1–24. https://doi.org/10.1007/s11145-016-9660-x | 4. Reading-related but it is not neither a training or technological |
| Palincsar, A. S., Fitzgerald, M. S., Marcum, M. B., & Sherwood, C.-A. (2018). Examining the work of ``scaffolding{’’} in theory and practice: A case study of 6th graders and their teacher interacting with one another, an ambitious science curriculum, and mobile devices. INTERNATIONAL JOURNAL OF EDUCATIONAL RESEARCH, 90, 191–208. https://doi.org/10.1016/j.ijer.2017.11.006 | 3. Technological training that is not focus on improving reading |
| Palincsar, A. S., Fitzgerald, M. S., Marcum, M. B., & Sherwood, C.-A. (2018). Examining the work of ``scaffolding{’’} in theory and practice: A case study of 6th graders and their teacher interacting with one another, an ambitious science curriculum, and mobile devices. INTERNATIONAL JOURNAL OF EDUCATIONAL RESEARCH, 90, 191–208. https://doi.org/10.1016/j.ijer.2017.11.006 | 3. Technological training that is not focus on improving reading |
| Palmer, S. L., Leigh, L., Ellison, S. C., Onar-Thomas, A., Wu, S., Qaddoumi, I., … Gajjar, A. (2014). Feasibility and efficacy of a computer-based intervention aimed at preventing reading decoding deficits among children undergoing active treatment for Medulloblastoma: Results of a randomized trial. Journal of Pediatric Psychology. Oxford University Press. https://doi.org/10.1093/jpepsy/jst095 | 8. Training oriented to special population (other than dyslexia) |
| Pannim, P., Suwannatthachote, P., & Numprasertchai, S. (2018). Investigation of Instructional Design on Reading Comprehension Affect the Demand for Mobile Application for Students with Learning Disabilities. 2018 2ND INTERNATIONAL CONFERENCE ON EDUCATION AND E-LEARNING (ICEEL 2018), 104–108. https://doi.org/10.1145/3291078.3291100 | 5. Technology but it is not neither a training or reading-related |
| Pannim, P., Suwannatthachote, P., & Numprasertchai, S. (2018). Investigation of instructional design on reading comprehension affect the demand for mobile application for students with learning disabilities. ACM International Conference Proceeding Series, 104–108. https://doi.org/10.1145/3291078.3291100 | 2. It is a reading-related technology but it is not a training (test, support…) |
| Pareja-Lora, A., Calle-Martínez, C., & Rodríguez-Arancón, P. (2016). New Perspectives on Teaching and Working with Languages in the Digital Era. Research-publishing.net. Research-publishing.net. https://doi.org/10.14705/rpnet.2016.tislid2014.9781908416353 | 2. It is a reading-related technology but it is not a training (test, support…) |
| Park, H.-R., & Kim, D. (2015). English Language Learners’ Strategies for Reading Computer-Based Texts at Home and in School. CALICO Journal, 0(0), 380–409. https://doi.org/10.1558/cj.v33i3.26552 | 9. Training oriented to a second-language learning |
| Park, H.-R., Kim, D., & Vorobel, O. (2019). International students’ reading digital texts on tablets: experiences and strategies. Journal of Computing in Higher Education. https://doi.org/10.1007/s12528-019-09242-x | 5. Technology but it is not neither a training or reading-related |
| Park, J. H., Kim, H.-Y., & Lim, S.-B. (2019). Development of an electronic book accessibility standard for physically challenged individuals and deduction of a production guideline. Computer Standards & Interfaces, 64, 78–84. http://10.0.3.248/j.csi.2018.12.004 | 8. Training oriented to special population (other than dyslexia) |
| Pee, N. C., Mohtaram, S., Shibgatullah, A. S., Sulaiman, H. A., Othman, M. F. I., Rahim, Y. A., & Othman, M. A. (2017). The framework of mobile dyslexia screening test using multiple-deficit theories. Advanced Science Letters, 23(5), 4000–4004. https://doi.org/10.1166/asl.2017.8344 | 2. It is a reading-related technology but it is not a training (test, support…) |
| Pee, N. C., Mohtaram, S., Shibgatullah, A. S., Sulaiman, H. A., Othman, M. F. I., Rahim, Y. A., & Othman, M. A. (2017). The framework of mobile dyslexia screening test using multiple-deficit theories. Advanced Science Letters, 23(5), 4000–4004. https://doi.org/10.1166/asl.2017.8344 | 2. It is a reading-related technology but it is not a training (test, support…) |
| Penichet, Victor M. R., Peñalver, A., Gallud, J. A., & Penichet, V. M. R. (2013). New Trends in Interaction, Virtual Reality and Modeling. London : Springer London : Imprint: Springer, 2013. Retrieved from http://ezproxy.si.unav.es:2048/login?url=http://search.ebscohost.com/login.aspx?direct=true&AuthType=ip,url&db=edshlc&AN=edshlc.013818842-4&lang=es&site=eds-live&scope=site | 5. Technology but it is not neither a training or reading-related |
| Perlman, E. H. (2016). the Use of Computerized Cognitive Assessment With Children With Autism. Dissertation Abstracts International Section A: Humanities and Social Sciences. ProQuest Information & Learning, US. Retrieved from http://ezproxy.si.unav.es:2048/login?url=http://search.ebscohost.com/login.aspx?direct=true&AuthType=ip,url&db=psyh&AN=2017-05717-144&lang=es&site=eds-live&scope=site | 8. Training oriented to special population (other than dyslexia) |
| Petrou, A. (2015). Tablets Change the Face of the Special Education. a Case Study in Greece Early Results - Early Findings. In Chova, LG and Martinez, AL and Torres, IC (Ed.), Inted2015: 9Th International Technology, Education and Development Conference (pp. 5853–5860). | 8. Training oriented to special population (other than dyslexia) |
| Peyre, H., Gérard, C. L., Dupong Vanderhorst, I., Larger, S., Lemoussu, C., Vesta, J., … Bucci, M. P. (2016). Computerized oculomotor training in dyslexia: A randomized, crossover clinical trial in pediatric population. Encephale. https://doi.org/10.1016/j.encep.2017.03.004 | 7. Technological reading training that do not fulfill methods requirements |
| Peyre, H., Gérard, C.-L., Dupong Vanderhorst, I., Larger, S., Lemoussu, C., Vesta, J., Bui Quoc, E., Gouleme, N., Delorme, R., & Bucci, M. P. (2018). Computerized oculomotor training in dyslexia: A randomized, crossover clinical trial in pediatric population [Rééducation oculomotrice informatisée dans la dyslexie : essai clinique randomisé en crossover en population pédiatrique]. Encephale, 44(3), 247–255. https://doi.org/10.1016/j.encep.2017.03.004 | 5. Technology but it is not neither a training or reading-related |
| Peyre, H., Gérard, C.-L., Dupong Vanderhorst, I., Larger, S., Lemoussu, C., Vesta, J., Bui Quoc, E., Gouleme, N., Delorme, R., & Bucci, M. P. (2018). Computerized oculomotor training in dyslexia: A randomized, crossover clinical trial in pediatric population [Rééducation oculomotrice informatisée dans la dyslexie : essai clinique randomisé en crossover en population pédiatrique]. Encephale, 44(3), 247–255. https://doi.org/10.1016/j.encep.2017.03.004 | 7. Technological reading training that do not fulfill methods requirements |
| Pfenninger, S. E. (2015). MSL in the digital ages: Effects and effectiveness of computer-mediated intervention for FL learners with dyslexia. Studies in Second Language Learning and Teaching, 5(1), 109. https://doi.org/10.14746/ssllt.2015.5.1.6 | 9. Training oriented to a second-language learning |
| Pham, V. K., Nguyen, H. D., & Tran, M. T. (2015). Virtual music teacher for new music learners with optical music recognition. In Zaphiris, P and Ioannou, A (Ed.), Lecture Notes in Computer Science (including subseries Lecture Notes in Artificial Intelligence and Lecture Notes in Bioinformatics) (Vol. 9192, pp. 415–426). https://doi.org/10.1007/978-3-319-20609-7_39 | 3. Technological training that is not focus on improving reading |
| Philiyanti, F., Haristiani, N., Rasyid, Y., & Emzir. (2019). ANDROID-BASED LEARNING MEDIA IN CONTEXTUAL TEACHING AND LEARNING ON JAPANESE LANGUAGE READING. JOURNAL OF ENGINEERING SCIENCE AND TECHNOLOGY, 14(3), 1138–1149. | 9. Training oriented to a second-language learning |
| Pierce, J. B. (2009). Inspiring Young Readers. American Libraries, 40(10), 70. Retrieved from http://ezproxy.si.unav.es:2048/login?url=http://search.ebscohost.com/login.aspx?direct=true&AuthType=ip,url&db=asx&AN=44715637&lang=es&site=eds-live&scope=site | 2. It is a reading-related technology but it is not a training (test, support…) |
| Pieschl, S., Bromme, R., & Stahl, E. (2010). What can hypertext re-reading tell us about the design of adaptive (metacognitive) help functions? In AAAI Fall Symposium - Technical Report (Vol. FS-10-01, pp. 75–80). University of Muenster, Institute for Psychology, Fliednerstr. 21, 48149 Muenster, Germany. Retrieved from http://www.scopus.com/inward/record.url?eid=2-s2.0-79960129632&partnerID=tZOtx3y1 | 3. Technological training that is not focus on improving reading |
| Pillar, G. A., Prudente, M. S., & Aguja, S. E. (2015). Using interactive applications in enhancing students??? knowledge and technical skills and process skills in grade 4 science. Advanced Science Letters, 21(7), 2266–2270. https://doi.org/10.1166/asl.2015.6289 | 3. Technological training that is not focus on improving reading |
| Pindiprolu, S. S., & Forbush, D. E. (2009). Evaluating the Promise of Computer-Based Reading Interventions with Students with Reading Difficulties. Journal on School Educational Technology, 4(3), 41–49. Retrieved from https://login.pallas2.tcl.sc.edu/login?url=http://search.ebscohost.com/login.aspx?direct=true&db=eric&AN=EJ1102828&site=ehost-live | 12. Other: No access granted, same data… |
| Pinnelli, S., Salento, U., Pistoia, M., Pinnelli, S., & Borrelli, G. (2015). Use of a robotic platform in dyslexia-affected pupils : the ROBIN project experience Use of a robotic platform in dyslexia-affected pupils : the ROBIN project experience. INTERNATIONAL JOURNAL OF EDUCATION AND INFORMATION TECHNOLOGIES, 9(SEPTEMBER), 46–49. Retrieved from https://www.researchgate.net/profile/Stefania_Pinnelli/publication/282102086_Use_of_a_robotic_platform_in_dyslexia-affected_pupils_the_ROBIN_project_experience/links/5602c6fd08ae3b544e35e87a.pdf | 3. Technological training that is not focus on improving reading |
| Pinter, R., Radosav, D., & ??isar, S. M. (2010). Learning styles and reading on screen. In SIISY 2010 - 8th IEEE International Symposium on Intelligent Systems and Informatics (pp. 159–161). Subotica Tech., Subotica, Serbia. https://doi.org/10.1109/SISY.2010.5647271 | 2. It is a reading-related technology but it is not a training (test, support…) |
| Piotrowska, B., Willis, A., & Kerridge, J. (2015). Performance on a novel visual-spatial- motor task may predict reading difficulties in children. Psychology of Education Review, 39(2), 42–48. Retrieved from http://ezproxy.si.unav.es:2048/login?url=http://search.ebscohost.com/login.aspx?direct=true&AuthType=ip,url&db=pbh&AN=109952026&lang=es&site=eds-live&scope=site | 2. It is a reading-related technology but it is not a training (test, support…) |
| Pisanu, F., & Freise, L. (2017). EFFECTS OF ICTS’ TRAINING FOR TEACHERS IN PRIMARY SCHOOL ON STUDENTS’ COMPETENCIES, MOTIVATION AND PERCEIVED CLASSROOM CLIMATE. In Beseda, J and Rohlikova, L and Batko, J (Ed.), DISCO 2017: OPEN EDUCATION AS A WAY TO A KNOWLEDGE SOCIETY (pp. 24–39). | 10. Educational-related but it is not about reading |
| Plavnick  plavnick@msu.edu, J., Thompson, J., Englert, C., Mariage, T., & Johnson, K. (2016). Mediating Access to Headsprout Early Reading for Children with Autism Spectrum Disorders. Journal of Behavioral Education, 25(3), 357–378. Retrieved from http://10.0.3.239/s10864-015-9244-x%5Cnhttp://search.ebscohost.com/login.aspx?direct=true&db=eue&AN=117353786&site=ehost-live&scope=site | 8. Training oriented to special population (other than dyslexia) |
| Plavnick, J. B. ., & Savana Bak, M. Y. . (2016). Description of a Computer-Based Reading Program for Children with Autism Spectrum Disorder. Michigan Reading Journal, 48(3), 51–56. Retrieved from http://search.ebscohost.com/login.aspx?direct=true&db=eue&AN=115642204&site=ehost-live&scope=site | 8. Training oriented to special population (other than dyslexia) |
| Poehner, M. E., & Lantolf, J. P. (2013). Bringing the ZPD into the equation: Capturing L2 development during Computerized Dynamic Assessment (C-DA). Language Teaching Research, 17(3), 323–342. https://doi.org/10.1177/1362168813482935 | 9. Training oriented to a second-language learning |
| Poitras, E., & Trevors, G. (2012). Deriving Empirically-Based Design Guidelines for Advanced Learning Technologies that Foster Disciplinary Comprehension. Canadian Journal of Learning and Technology, 38(1), 1–21. Retrieved from http://cjlt.csj.ualberta.ca/index.php/cjlt/article/view/634 | 2. It is a reading-related technology but it is not a training (test, support…) |
| Ponce, H. R., & Mayer, R. E. (2014). An eye movement analysis of highlighting and graphic organizer study aids for learning from expository text. Computers in Human Behavior, 41, 21–32. https://doi.org/10.1016/j.chb.2014.09.010 | 2. It is a reading-related technology but it is not a training (test, support…) |
| Ponsard, C., & Fries, V. (2008). An accessible viewer for digital comic books. Lecture Notes in Computer Science (Including Subseries Lecture Notes in Artificial Intelligence and Lecture Notes in Bioinformatics). CETIC Research Center, Charleroi, Belgium. https://doi.org/10.1007/978-3-540-70540-6_81 | 2. It is a reading-related technology but it is not a training (test, support…) |
| Potocki, A., Ecalle, J., & Magnan, A. (2015). Computerized comprehension training for whom and under which conditions is it efficient? Journal of Computer Assisted Learning, 31(2), 162–175. https://doi.org/10.1111/jcal.12087 | 7. Technological reading training that do not fulfill methods requirements |
| Potter, C., Ravenscroft, G., & Fridjhon, P. (2010). USING COMPUTER-BASED ERROR ANALYSIS TO IDENTIFY CHILDREN WITH LEARNING DIFFICULTIES. In Chova, LG and Belenguer, DM and Torres, IC (Ed.), 4TH INTERNATIONAL TECHNOLOGY, EDUCATION AND DEVELOPMENT CONFERENCE (INTED 2010) (pp. 211–221). | 2. It is a reading-related technology but it is not a training (test, support…) |
| Prabhu, M. T. (2010). Software programs help English-language learners. ESchool News, 13(1), 9. Retrieved from http://ezproxy.si.unav.es:2048/login?url=http://search.ebscohost.com/login.aspx?direct=true&AuthType=ip,url&db=edo&AN=47692122&lang=es&site=eds-live&scope=site | 9. Training oriented to a second-language learning |
| Premchaiswadi, W., & Premchaiswadi, N. (2011). Mobile application for learning the Thai language. In Proceedings of the 15th WSEAS … (pp. 445–452). Graduate School of Information Technology in Business, Siam University, 38 Petkasem Rd., Phasicharoen, Bangkok, 10160, Thailand. Retrieved from http://www.wseas.us/e-library/conferences/2011/Corfu/COMPUTERS/COMPUTERS-75.pdf | 7. Technological reading training that do not fulfill methods requirements |
| Pretorius, M., Gelderblom, H., & Chimbo, B. (2010). Using eye tracking to compare how adults and children learn to use an unfamiliar computer game. In Proceedings of the 2010 Annual Research Conference of the South African Institute of Computer Scientists and Information Technologists on - SAICSIT ’10 (pp. 275–283). School of Computing, UNISA, Preller Street, Pretoria, South Africa. https://doi.org/10.1145/1899503.1899534 | 3. Technological training that is not focus on improving reading |
| Prist, M., Cavanini, L., Longhi, S., Monteriu, A., Ortenzi, D., & Freddi, A. (2014). A low cost mobile platform for educational robotic applications. In MESA 2014 - 10th IEEE/ASME International Conference on Mechatronic and Embedded Systems and Applications, Conference Proceedings. Dipartimento di Ingegneria dell’Informazione, Università Politecnica Delle Marche, Via Brecce Bianche, Ancona, Italy: Institute of Electrical and Electronics Engineers Inc. https://doi.org/10.1109/MESA.2014.6935571 | 10. Educational-related but it is not about reading |
| Protopapas, A. (2008). Validation of Unsupervised Computer-Based Screening for. Learning Disabilities -- A Contemporary Journal, 6(1), 45–69. Retrieved from http://ezproxy.si.unav.es:2048/login?url=http://search.ebscohost.com/login.aspx?direct=true&AuthType=ip,url&db=eric&AN=EJ797660&lang=es&site=eds-live&scope=site | 2. It is a reading-related technology but it is not a training (test, support…) |
| Proudfoot, D. E. (2016). The effect of a reading comprehension software program on student achievement in mathematics. International Journal of Cognitive Research in Science, Engineering and Education. https://doi.org/10.5937/IJCRSEE1601039P | 7. Technological reading training that do not fulfill methods requirements |
| Puff Simmons, M. D. (2013). Empirically-Validated High School Classroom-Based Interventions Designed to Address Executive Skills Dysfunction in Middle Adolescent Males and the Informed Practice of School Psychology. ProQuest Dissertations and Theses. ProQuest Information & Learning, US. Retrieved from http://oxfordsfx.hosted.exlibrisgroup.com/oxford?url_ver=Z39.88-2004&rft_val_fmt=info:ofi/fmt:kev:mtx:dissertation&genre=dissertations+%2526+theses&sid=ProQ:ProQuest+Dissertations+%2526+Theses+Global&atitle=&title=Empirically-Validated+High+School+Classro | 8. Training oriented to special population (other than dyslexia) |
| Puolakanaho, A., & Latvala, J.-M. (2017). Embedding preschool assessment methods into digital learning games to predict early reading skills. Human Technology, 13(2), 216–236. https://doi.org/10.17011/ht/urn.201711104212 | 2. It is a reading-related technology but it is not a training (test, support…) |
| Purkayastha, S., Nehete, N., & Purkayastha, J. (2012). Dyscover - An Orton-Gillingham approach inspired multi-sensory learning application for dyslexic children. In Proceedings of the 2012 World Congress on Information and Communication Technologies, WICT 2012 (pp. 685–690). Department of Computer and Information Science, Norwegian University of Science and Technology, Trondheim, Norway. https://doi.org/10.1109/WICT.2012.6409163 | 7. Technological reading training that do not fulfill methods requirements |
| Purrazzella, K., & Mechling, L. C. (2013). Evaluation of manual spelling, observational and incidental learning using computer-based instruction with a tablet PC, large screen projection, and a forward chaining procedure. Education and Training in Autism and Developmental Disabilities, 48(2), 218–235. Retrieved from http://ezproxy.si.unav.es:2048/login?url=http://search.ebscohost.com/login.aspx?direct=true&AuthType=ip,url&db=edsjsr&AN=edsjsr.23880641&lang=es&site=eds-live&scope=site | 8. Training oriented to special population (other than dyslexia) |
| Qader, O. J. A., & Ibraheem, N. M. (2019). Mobile games effects on visual acuity of primary school students and the role of chewable multivitamins in the improvement. Indian Journal of Forensic Medicine and Toxicology, 13(4), 1419–1424. https://doi.org/10.5958/0973-9130.2019.00501.2 | 10. Educational-related but it is not about reading |
| Qian, K., Owen, N., & Bax, S. (2018). Researching mobile-assisted Chinese-character learning strategies among adult distance learners. INNOVATION IN LANGUAGE LEARNING AND TEACHING, 12(1, SI), 56–71. https://doi.org/10.1080/17501229.2018.1418633 | 9. Training oriented to a second-language learning |
| Quezada-Sarmiento, P. A., Espinoza, V., Ulehlova, E., & Enciso-Quispe, L. (2017). Application of cloud, knowledge and innovation model for improving linguistic in writing skill [Implementación de modelo cloud, conocimiento e innovación para la mejora lingüística de la destreza de escritura]. In A. B. C. C. C. M. P. Reis L.P. Rocha A. (Ed.), Iberian Conference on Information Systems and Technologies, CISTI. IEEE Computer Society. https://doi.org/10.23919/CISTI.2017.7975903 | 3. Technological training that is not focus on improving reading |
| Quiña-Mera, A., Barahona, S. P., Guevara-Vega, C., García-Santillán, I., Guevara-Vega, A., & Yugla, J. M. (2019). Use of gamification in the learning of children with dyseidetic disexia: A case study. RISTI - Revista Iberica de Sistemas e Tecnologias de Informacao, 2019(E22), 161–173. https://www.scopus.com/inward/record.uri?eid=2-s2.0-85075273273&partnerID=40&md5=eb3b146d7ac17c090554372682d2be1f | 2. It is a reading-related technology but it is not a training (test, support…) |
| R., M., G., W., H.-S., Y., H., H., S., K., & A., L. (Eds.). (2016). 15th IFIP TC 14 International Conference on Entertainment Computing, ICEC 2016. In Lecture Notes in Computer Science (including subseries Lecture Notes in Artificial Intelligence and Lecture Notes in Bioinformatics): Vol. 9926 LNCS (pp. 1–291). Springer Verlag. https://www.scopus.com/inward/record.uri?eid=2-s2.0-84990063433&partnerID=40&md5=9f06d735837cd829d5e1b93080a7cf4d | 11. Type of study excluded (review, non-primary references…) |
| Rabiner, D. L., Murray, D. W., Skinner, A. T., & Malone, P. S. (2010). A randomized trial of two promising computer-based interventions for students with attention difficulties. Journal of Abnormal Child Psychology, 38(1), 131–142. https://doi.org/10.1007/s10802-009-9353-x | 8. Training oriented to special population (other than dyslexia) |
| Rachmawati, D., & Asmara, C. C. H. (2017). Reading And Writing: Development Of Project-based Learning (PBL) Approach. In Rakhmawati (Ed.), PROCEEDINGS OF THE INTERNATIONAL CONFERENCE ON ENGLISH LANGUAGE TEACHING (ICONELT 2017) (Vol. 145, pp. 48–53). | 10. Educational-related but it is not about reading |
| Radecki, A., Bujacz, M., Skulimowski, P., & Strumiłło, P. (2019). Interactive sonification of images in serious games as an education aid for visually impaired children. British Journal of Educational Technology. https://doi.org/10.1111/bjet.12852 | 8. Training oriented to special population (other than dyslexia) |
| Radner, W., Diendorfer, G., Kainrath, B., & Kollmitzer, C. (2017). The accuracy of reading speed measurement by stopwatch versus measurement with an automated computer program (RAD-RD (c)). ACTA OPHTHALMOLOGICA, 95(2), 211–216. https://doi.org/10.1111/aos.13201 | 2. It is a reading-related technology but it is not a training (test, support…) |
| Radner, W., Diendorfer, G., Kainrath, B., & Kollmitzer, C. (2017). The accuracy of reading speed measurement by stopwatch versus measurement with an automated computer program (rad-rd©). Acta Ophthalmologica, 95(2), 211–216. https://doi.org/10.1111/aos.13201 | 2. It is a reading-related technology but it is not a training (test, support…) |
| Radner, W., Diendorfer, G., Kainrath, B., & Kollmitzer, C. (2017). The accuracy of reading speed measurement by stopwatch versus measurement with an automated computer program (rad-rd©). Acta Ophthalmologica, 95(2), 211–216. https://doi.org/10.1111/aos.13201 | 2. It is a reading-related technology but it is not a training (test, support…) |
| Radu, N.-I., Petz, A., & Miesenberger, K. (2013). CAPKOM: A wizard to facilitate the Web experience of users with cognitive disabilities. (E. P., A. L., G. G.J., N. A., & M. N.E., Eds.), Assistive Technology Research Series. Johannes Kepler University of Linz, Institute Integriert Studieren, Linz, Austria. https://doi.org/10.3233/978-1-61499-304-9-976 | 8. Training oriented to special population (other than dyslexia) |
| Rahim, S. K. N. A., Nasrudin, N. H., Azmi, A. Z., Junid, R. A., Mohamed, Z., & Abdullah, I. I. B. (2018). Designing Mobile Application for Dyslexia in Reading Disorder Problem. International Journal of Academic Research in Business and Social Sciences VO  - 8, 1, 628. https://ezproxy.unav.es/login?url=https://search.ebscohost.com/login.aspx?direct=true&AuthType=ip,url&db=edsrep&AN=edsrep.a.hur.ijarbs.v8y2018i1p628.646&lang=es&site=eds-live&scope=site | 7. Technological reading training that do not fulfill methods requirements |
| Rahmawati, A., Kaburuan, E. R., Arifianto, A., & Juniati, N. K. (2019). Ciselexia: Computer-based method for improving self-awareness in children with dyslexia. Advances in Science, Technology and Engineering Systems, 4(5), 258–267. https://doi.org/10.25046/aj040532 | 3. Technological training that is not focus on improving reading |
| Raja, B. W. D., & Kumar, S. P. (2010). Do Multimedia Applications Benefit Learning-Disabled Children? Journal of Educational Technology, 6(4), 1–7. Retrieved from http://ezproxy.lib.swin.edu.au/login?url=http://search.ebscohost.com/login.aspx?direct=true&db=eric&AN=EJ1098361&site=ehost-live&scope=site | 11. Type of study excluded (review, non-primary references…) |
| Rajapakse, S., Polwattage, D., Guruge, U., Jayathilaka, I., Edirisinghe, T., & Thelijjagoda, S. (2018). ALEXZA: A Mobile Application For Dyslexics Utilizing Artificial Intelligence And Machine Learning Concepts. 2018 3RD INTERNATIONAL CONFERENCE ON INFORMATION TECHNOLOGY RESEARCH (ICITR). | 2. It is a reading-related technology but it is not a training (test, support…) |
| Rajapakse, S., Polwattage, D., Guruge, U., Jayathilaka, I., Edirisinghe, T., & Thelijjagoda, S. (2018). ALEXZA: A Mobile Application For Dyslexics Utilizing Artificial Intelligence And Machine Learning Concepts. 2018 3RD INTERNATIONAL CONFERENCE ON INFORMATION TECHNOLOGY RESEARCH (ICITR). | 2. It is a reading-related technology but it is not a training (test, support…) |
| Rajendran, R., Kumar, A., Carter, K. E., Levin, D. T., & Biswas, G. (2018). Predicting learning by analyzing eye-gaze data of reading behavior. In Y. M. Boyer K.E. (Ed.), Proceedings of the 11th International Conference on Educational Data Mining, EDM 2018. International Educational Data Mining Society. https://www.scopus.com/inward/record.uri?eid=2-s2.0-85067790754&partnerID=40&md5=08eaa3e6e65ab6603dad4e776079ab36 | 5. Technology but it is not neither a training or reading-related |
| Ramachandran, S., & Atkinson, R. (2008). An evaluation of intelligent reading tutors. Lecture Notes in Computer Science (Including Subseries Lecture Notes in Artificial Intelligence and Lecture Notes in Bioinformatics). Stottler Henke Associates, Inc, San Mateo, CA, United States. https://doi.org/10.1007/978-3-540-69132-7-92 | 7. Technological reading training that do not fulfill methods requirements |
| Ramdoss, S., Mulloy, A., Lang, R. B., O’Reilly, M. F., Sigafoos, J., Lancioni, G. E., … El Zein, F. (2011). Use of computer-based interventions to improve literacy skills in students with autism spectrum disorders: A systematic review. Research in Autism Spectrum Disorders, 5(4), 1306–1318. https://doi.org/10.1016/j.rasd.2011.03.004 | 8. Training oriented to special population (other than dyslexia) |
| Ramirez Flores, P. G., Mendoza Medina, J. A., Gonzalez Mendivil, E., Villegas Villarreal, A. R., Flores, P. G. R., Medina, J. A. M., Mendivil, E. G., & Villarreal, A. R. V. (2018). Using Augmented Reality and Kinect Technologies to Promote Reading Habits. Lecture Notes of the Institute for Computer Sciences, Social-Informatics and Telecommunications Engineering, LNICST, 213, 75–85. https://doi.org/10.1007/978-3-319-73323-4_8 | 2. It is a reading-related technology but it is not a training (test, support…) |
| Ramli, R., & Yusoff, Y. (2018). E-Iqra’: Mobile Application for Learning Al-Quran Using Voice Recognition. ADVANCED SCIENCE LETTERS, 24(3), 1666–1669. https://doi.org/10.1166/as1.2018.11133 | 2. It is a reading-related technology but it is not a training (test, support…) |
| Ramos-Ramirez, R., & Mauricio, D. (2019). Videogame to support the teaching of reading to deaf children using gamification. RISTI - Revista Iberica de Sistemas e Tecnologias de Informacao, 2019(E23), 145–157. https://www.scopus.com/inward/record.uri?eid=2-s2.0-85076184318&partnerID=40&md5=d2a90da51766ca9052e1f9464fe95fab | 8. Training oriented to special population (other than dyslexia) |
| Rasmusson, M. (2015). Reading paper - Reading screen: A comparison of reading literacy in two different modes. Nordic Studies in Education, 35(1), 3–19. Retrieved from http://ezproxy.si.unav.es:2048/login?url=http://search.ebscohost.com/login.aspx?direct=true&AuthType=ip,url&db=edsdun&AN=edsdun.66768099&lang=es&site=eds-live&scope=site | 2. It is a reading-related technology but it is not a training (test, support…) |
| Rasmusson, M., & Fredriksson, U. (2018). PISA, reading literacy, and computer-based assessment. Mittuniversitetet, Avdelningen för utbildningsvetenskap. https://doi.org/10.6027/TN2018-524 | 5. Technology but it is not neither a training or reading-related |
| Raspin, S., Smallwood, R., Hatfield, S., & Boesley, L. (2019). Exploring the use of the ARROW literacy intervention for looked after children in a UK local authority. EDUCATIONAL PSYCHOLOGY IN PRACTICE, 35(4), 411–423. https://doi.org/10.1080/02667363.2019.1632172 | 7. Technological reading training that do not fulfill methods requirements |
| Rauschenberger, M., Füchsel, S., Rello, L., Bayarri, C., & Thomaschewski, J. (2015). Exercises for German-speaking children with dyslexia. Lecture Notes in Computer Science (including subseries Lecture Notes in Artificial Intelligence and Lecture Notes in Bioinformatics) (Vol. 9296). https://doi.org/10.1007/978-3-319-22701-6_33 | 7. Technological reading training that do not fulfill methods requirements |
| Rauschenberger, M., Lins, C., Rousselle, N., Hein, A., & Fudickar, S. (2019). Designing a new puzzle app to target dyslexia screening in pre-readers. ACM International Conference Proceeding Series, 155–159. https://doi.org/10.1145/3342428.3342679 | 5. Technology but it is not neither a training or reading-related |
| Rauschenberger, M., Rello, L., & Baeza-Yates, R. (2018). A tablet game to target dyslexia screening in pre-readers. MobileHCI 2018 - Beyond Mobile: The Next 20 Years - 20th International Conference on Human-Computer Interaction with Mobile Devices and Services, Conference Proceedings Adjunct, 306–312. https://doi.org/10.1145/3236112.3236156 | 2. It is a reading-related technology but it is not a training (test, support…) |
| Razmi, M., Pourali, S., & Nozad, S. (2014). Digital Storytelling in EFL Classroom (Oral Presentation of the Story): A Pathway to Improve Oral Production. In Sadeghi, K and Modirkhameneh, S and Alavinia, P and Khonbi, ZA (Ed.), Procedia - Social and Behavioral Sciences (Vol. 98, pp. 1541–1544). https://doi.org/10.1016/j.sbspro.2014.03.576 | 9. Training oriented to a second-language learning |
| Rebolledo-Méndez, G., De Freitas, S., Rojano-Caceres, J. R., & Garcia-Gaona, A. R. (2010). An empirical examination of the relation between attention and motivation in computer-based education: A modeling approach. In Proceedings of the 23rd International Florida Artificial Intelligence Research Society Conference, FLAIRS-23 (pp. 74–79). Facultad de Estadística e Informática, Universidad Veracruzana, Jalapa, Mexico. Retrieved from http://www.scopus.com/inward/record.url?eid=2-s2.0-77957884286&partnerID=tZOtx3y1 | 10. Educational-related but it is not about reading |
| Reddy, L., Baghaei, N., Vermeulen, G., Hilton, C., & Steinhorn, G. (2017). Designing Mobile Applications for Improving Positive Behaviour for Learning (PB4L) Pedagogy. In Chen, W and Yang, JC and Ayub, AFM and Wong, SL and Mitrovic, A (Ed.), 25TH INTERNATIONAL CONFERENCE ON COMPUTERS IN EDUCATION (ICCE 2017): TECHNOLOGY AND INNOVATION: COMPUTER-BASED EDUCATIONAL SYSTEMS FOR THE 21ST CENTURY (pp. 990–995). | 10. Educational-related but it is not about reading |
| Reddy, L., Baghaei, N., Vermeulen, G., Hilton, C., & Steinhorn, G. (2017). Designing mobile applications for improving positive behaviour for learning (PB4L) pedagogy. In Y. J.-C. W. S. L. C. W. Mohd Ayub A.F. Mitrovic A. (Ed.), Proceedings of the 25th International Conference on Computers in Education, ICCE 2017 - Main Conference Proceedings (pp. 990–995). Asia-Pacific Society for Computers in Education. https://www.scopus.com/inward/record.uri?eid=2-s2.0-85053919665&partnerID=40&md5=5e4a32e55ce700b2c84a9830cf5553ae | 10. Educational-related but it is not about reading |
| Reed, P., Hughes, A., & Phillips, G. (2013). Rapid recovery in sub-optimal readers in Wales through a self-paced computer-based reading programme. British Journal of Special Education, 40(4), 162–166. https://doi.org/10.1111/1467-8578.12040 | 7. Technological reading training that do not fulfill methods requirements |
| Reeder, K., Shapiro, J., Early, M., Kendrick, M., & Wakefield, J. (2008). A Computer-Based Reading Tutor for Young Language Learners. In Handbook of Research on Computer-Enhanced Language Acquisition and Learning (pp. 159–188). Department of Language and Literacy Education, The University of British Columbia, Canada: IGI Global. https://doi.org/10.4018/978-1-59904-895-6.ch010 | 9. Training oriented to a second-language learning |
| Reitsma, P. (2009). Computer-based exercises for learning to read and spell by deaf children. Journal of Deaf Studies and Deaf Education, 14(2), 178–189. https://doi.org/10.1093/deafed/enn031 | 8. Training oriented to special population (other than dyslexia) |
| Rello, L., Ali, A., Romero, E., Williams, K., White, N. C., Rauschenberger, M., & Bigham, J. P. (2018). Screening dyslexia for english using HCI measures and machine learning. ACM International Conference Proceeding Series, 2018-April, 80–84. https://doi.org/10.1145/3194658.3194675 | 2. It is a reading-related technology but it is not a training (test, support…) |
| Rello, L., Kanvinde, G., & Baeza-Yates, R. (2012). A mobile application for displaying more accessible ebooks for people with dyslexia. Procedia Computer Science, 14(Dsai), 226–233. https://doi.org/10.1016/j.procs.2012.10.026 | 2. It is a reading-related technology but it is not a training (test, support…) |
| Richardson, J., Lenarcic, J., Mckay, E., & Craig, C. (2008). Pull with pearson’s trigger word tool to enable simplexity: Student empowerment via a text Messaging system for mobile administration. In Proceedings of the 7th IASTED International Conference on Web-Based Education, WBE 2008 (pp. 206–211). RMIT University, School of Business Information Technology, GPO Box 2476V, Melbourne, VIC 3001, Australia. Retrieved from http://www.scopus.com/inward/record.url?eid=2-s2.0-62949112388&partnerID=tZOtx3y1 | 5. Technology but it is not neither a training or reading-related |
| Ripamonti, L. A., & Maggiorini, D. (2011). Learning in virtual worlds: A new path for supporting cognitive impaired children. In Schmorrow, DD and Fidopiastis, CM (Ed.), Lecture Notes in Computer Science (including subseries Lecture Notes in Artificial Intelligence and Lecture Notes in Bioinformatics) (Vol. 6780 LNAI, pp. 462–471). https://doi.org/10.1007/978-3-642-21852-1_53 | 8. Training oriented to special population (other than dyslexia) |
| Rivera, L. P. R. (2017). Development of a reading-writing tools focused on speed reading for preschool children. In O. C. A. C. Gonzalez-Calleros J.M. Guerrero-Garcia J. (Ed.), ACM International Conference Proceeding Series: Vol. Part F1311. Association for Computing Machinery. https://doi.org/10.1145/3123818.3123819 | 7. Technological reading training that do not fulfill methods requirements |
| Rivero, T. S., N????ez, L. M. H., Pires, E. U., & Bueno, O. F. A. (2016). Corrigendum: ADHD rehabilitation through video gaming: A systematic review using prisma guidelines of the current findings and the associated risk of bias [Front Psychiatry, 6, 151, (2015)] doi: 10.3389/fpsyt.2015.00151. Frontiers in Psychiatry, 7(OCT). https://doi.org/10.3389/fpsyt.2016.00173 | 8. Training oriented to special population (other than dyslexia) |
| Roberts, G. J. G. J., Capin, P., Roberts, G. J. G. J., Miciak, J., Quinn, J. M., & Vaughn, S. (2018). Examining the Effects of Afterschool Reading Interventions for Upper Elementary Struggling Readers. Remedial and Special Education, 39(3), 131–143. https://doi.org/10.1177/0741932517750818 | 7. Technological reading training that do not fulfill methods requirements |
| Roberts-Tyler, E. J., Hughes, J. C., & Hastings, R. P. (2020). Evaluating a computer-based reading programme with children with Intellectual Disabilities: feasibility and pilot research. JOURNAL OF RESEARCH IN SPECIAL EDUCATIONAL NEEDS, 20(1), 14–26. https://doi.org/10.1111/1471-3802.12458 | 8. Training oriented to special population (other than dyslexia) |
| Robles-Bykbaev, V., Guzhñay-Lucero, A., Pulla-Sánchez, D., Pesántez-Avilés, F., Suquilanda-Cuesta, P., & Bernal-Merchán, E. (2018). A Multifunction Braille Trainer based on Embedded Systems, Mobile Apps, Rule-based Reasoning and Data Mining for Children with Visual Impairment [Un entrenador de Braille multifunción basado en sistemas embebidos, aplicaciones móviles, razonamiento basado. Computacion y Sistemas, 22(4), 1487–1502. https://doi.org/10.13053/CyS-22-4-2795 | 8. Training oriented to special population (other than dyslexia) |
| Rocha, J., Magalhaes, L., Alves, N., & Guevara, M. (2019). Inpresso AR: A generic augmented book. In F. M. Melo M.C. Campos P.F. (Ed.), ICGI 2019 - Proceedings of the International Conference on Graphics and Interaction (pp. 1–7). Institute of Electrical and Electronics Engineers Inc. https://doi.org/10.1109/ICGI47575.2019.8955067 | 10. Educational-related but it is not about reading |
| Rodriguez, C. D., Filler, J., Higgins, K., & van den Berg, B. (2012). Using Primary Language Support via Computer to Improve Reading Comprehension Skills of First-Grade English Language Learners. Computers in the Schools, 29(3), 253–267. https://doi.org/10.1080/07380569.2012.702718 | 9. Training oriented to a second-language learning |
| Rolka, E. J., & Silverman, M. J. (2015). A systematic review of music and dyslexia. Arts in Psychotherapy, 46, 24–32. https://doi.org/10.1016/j.aip.2015.09.002 | 11. Type of study excluded (review, non-primary references…) |
| Rosas, R., Escobar, J. P., Ramírez, M. P., Meneses, A., & Guajardo, A. (2017). Impact of a computer-based intervention in Chilean children at risk of manifesting reading difficulties / Impacto de una intervención basada en ordenador en niños chilenos con riesgo de manifestar dificultades lectoras. Infancia y Aprendizaje, 40(1), 158–188. https://doi.org/10.1080/02103702.2016.1263451 | 11. Type of study excluded (review, non-primary references…) |
| Rosas, R., Escobar, J.-P., Ramírez, M.-P., Meneses, A., & Guajardo, A. (2017). Impact of a computer-based intervention in Chilean children at risk of manifesting reading difficulties / Impacto de una intervención basada en ordenador en niños chilenos con riesgo de manifestar dificultades lectoras. Infancia y Aprendizaje, 40(1), 158–188. https://doi.org/10.1080/02103702.2016.1263451 | 12. Other: No access granted, same data… |
| Rosas, R., Escobar, J.-P., Ramirez, M.-P., Meneses, A., & Guajardo, A. (2017). Impact of a computer-based intervention in Chilean children at risk of manifesting reading difficulties. Infancia Y Aprendizaje, 40(1), 158–188. https://doi.org/10.1080/02103702.2016.1263451 | 12. Other: No access granted, same data… |
| Rossetto, A. D. F., & Dutra, A. (2016). The analysis of the use of mobile technology in 6-8 years old children’s literacy process. In GarciaPenalvo, FJ and Mendes, AJ (Ed.), 2016 International Symposium on Computers in Education, SIIE 2016: Learning Analytics Technologies. PPGEN-UTFPR, Londrina, Brazil: Institute of Electrical and Electronics Engineers Inc. https://doi.org/10.1109/SIIE.2016.7751830 | 7. Technological reading training that do not fulfill methods requirements |
| Ruffino, P. (2018). Indie Game Studies workshop. https://ezproxy.unav.es/login?url=https://search.ebscohost.com/login.aspx?direct=true&AuthType=ip,url&db=edsbas&AN=edsbas.509ECC3E&lang=es&site=eds-live&scope=site | 11. Type of study excluded (review, non-primary references…) |
| Rusmanayanti, A., & Hanafi, M. L. (2018). Teaching Reading Comprehension by Using Computer-Based Reading: An Experimental Study in Indonesian English Language Teaching. Arab World English Journal, 202. https://ezproxy.unav.es/login?url=https://search.ebscohost.com/login.aspx?direct=true&AuthType=ip,url&db=edb&AN=130976671&lang=es&site=eds-live&scope=site | 9. Training oriented to a second-language learning |
| Ryan, C. S. (2017). Learning Disabilities: An International Perspective. In IntechOpen. IntechOpen. https://ezproxy.unav.es/login?url=https://search.ebscohost.com/login.aspx?direct=true&AuthType=ip,url&db=eric&AN=ED588815&lang=es&site=eds-live&scope=site | 4. Reading-related but it is not neither a training or technological |
| S, V., L, K., S, K., & D, M. (2019). Typlotic Specification Using Mobile Application. https://ezproxy.unav.es/login?url=https://search.ebscohost.com/login.aspx?direct=true&AuthType=ip,url&db=edsbas&AN=edsbas.5010E775&lang=es&site=eds-live&scope=site | 8. Training oriented to special population (other than dyslexia) |
| Sachdeva, M. S. M. A., Sachdeva, M. S. M. A., Bakshi, M. Y., & Kumar, P. M. (2018). Camlens – An Innovative Android Phone Application To Empower The Blind And Visually Impaired In Reading Any Kind Of Printed Text In Real-Time Using Opencv, Optical Character Recognition And Text-To-Speech. https://doi.org/10.5281/ZENODO.1451741 | 8. Training oriented to special population (other than dyslexia) |
| Sainsbury, M., & Benton, T. (2011). Designing a formative e-assessment: Latent class analysis of early reading skills. British Journal of Educational Technology, 42(3), 500–514. https://doi.org/10.1111/j.1467-8535.2009.01044.x | 2. It is a reading-related technology but it is not a training (test, support…) |
| Sánchez, I. A., Isaías, P., & (IADIS), I. A. for D. of the I. S. (2013). Proceedings of the International Association for Development of the Information Society (IADIS) International Conference on Mobile Learning (Lisbon, Portugal, March 14-16, 2013). International Association for Development of the Information Society. International Association for Development of the Information Society. Retrieved from http://libezproxy.open.ac.uk/login?url=http://search.ebscohost.com/login.aspx?direct=true&db=eric&AN=ED562140&site=ehost-live&scope=site | 11. Type of study excluded (review, non-primary references…) |
| Sargeant, B., & Mueller, F. (2018). How Far is Up? Bringing the counterpointed triad technique to digital storybook apps. Conference on Human Factors in Computing Systems - Proceedings, 2018-April. https://doi.org/10.1145/3173574.3174093 | 2. It is a reading-related technology but it is not a training (test, support…) |
| Savage, R. S., Abrami, P., Hipps, G., & Deault, L. (2009). A randomized controlled trial study of the ABRACADABRA reading intervention program in grade 1. Journal of Educational Psychology, 101(3), 590–604. https://doi.org/10.1037/a0014700 | 12. Other: No access granted, same data… |
| Savindu, H. P., Iroshan, K. A., Panangala, C. D., Perera, W. L. D. W. P. L. D. W. P., & De Silva, A. C. (2017). BrailleBand: Blind support haptic wearable band for communication using braille language. 2017 IEEE INTERNATIONAL CONFERENCE ON SYSTEMS, MAN, AND CYBERNETICS (SMC), 2017-Janua, 1381–1386. https://doi.org/10.1109/SMC.2017.8122806 | 8. Training oriented to special population (other than dyslexia) |
| Scheithauer, M. C., & Tiger, J. H. (2012). a Computer-Based Program To Teach Braille Reading To Sighted Individuals. Journal of Applied Behavior Analysis. The Society for the Experimental Analysis of Behavior. https://doi.org/10.1901/jaba.2012.45-315 | 8. Training oriented to special population (other than dyslexia) |
| Scheithauer, M. C., Tiger, J. H., & Miller, S. J. (2013). On the efficacy of a computer-based program to teach visual braille reading. Journal of Applied Behavior Analysis. https://doi.org/10.1002/jaba.48 | 8. Training oriented to special population (other than dyslexia) |
| Schmidt-Naylor, A. C., Saunders, K. J., & Brady, N. C. (2017). Developing the alphabetic principle to aid text-based augmentative and alternative communication use by adults with low speech intelligibility and intellectual disabilities. American Journal of Speech-Language Pathology, 26(2), 397–412. https://doi.org/10.1044/2016_AJSLP-15-0047 | 8. Training oriented to special population (other than dyslexia) |
| Schrader, J., Stuber, L., & Wedwick, L. (2012). Authenticating Accelerated Reader : Collaborative Goal-setting Within the Context of AR. Illinois Reading Council Journal, 40(3), 14–22. Retrieved from http://ezproxy.si.unav.es:2048/login?url=http://search.ebscohost.com/login.aspx?direct=true&AuthType=ip,url&db=asx&AN=76146232&lang=es&site=eds-live&scope=site | 7. Technological reading training that do not fulfill methods requirements |
| Schrader, P. G., Archambault, L. M., & Oh-Young, C. (2011). Training by Gaming: Preparing Teachers of Today for Tomorrow’s Learning Environments. Journal of Technology and Teacher Education, 19(3), 261–286. Retrieved from http://www.proxy.its.virginia.edu/login?url=http://search.ebscohost.com/login.aspx?direct=true&db=eric&AN=EJ944272&site=ehost-live%5Cnhttp://www.editlib.org/j/JTATE/v/19/n/3 | 10. Educational-related but it is not about reading |
| Securro Jr., S., Jones, J. D., & Cantrell, D. R. (2010). Effects of Extensive Engagement with Computer-Based Reading and Language Arts Instructional Software on Reading Achievement for Sixth Graders. Journal on School Educational Technology, 6(1), 60–69. Retrieved from http://search.ebscohost.com/login.aspx?direct=true&db=eric&AN=EJ1102789&site=ehost-live | 7. Technological reading training that do not fulfill methods requirements |
| Semali, L. M., & Asino, T. I. (2014). Postliteracy in the digital age: The use of mobile phones to support literacy practices in Namibia and Tanzania. Prospects, 44(1), 81–97. https://doi.org/10.1007/s11125-012-9254-6 | 7. Technological reading training that do not fulfill methods requirements |
| Semenova, N. V., & Svyatkina, E. A. (2016). Digital textbook for vocationally-oriented informative reading in the research university. In Chugunov, AV and Bolgov, R and Kabanov, Y and Kampis, G and Wimmer, M (Ed.), Communications in Computer and Information Science (Vol. 674, pp. 363–369). https://doi.org/10.1007/978-3-319-49700-6_34 | 2. It is a reading-related technology but it is not a training (test, support…) |
| Serrano, M. Á., Vidal-Abarca, E., Máñez, I., & Candel, C. (2015). Selection task and computer-based feedback to improve the searching process in task-oriented reading situations. Lecture Notes in Computer Science (including subseries Lecture Notes in Artificial Intelligence and Lecture Notes in Bioinformatics) (Vol. 9112). https://doi.org/10.1007/978-3-319-19773-9_120 | 3. Technological training that is not focus on improving reading |
| Serrano-Laguna, Á., Torrente, J., Moreno-Ger, P., & Manjón, B. F. (2012). Tracing a little for big improvements: Application of learning analytics and videogames for student assessment. In Procedia Computer Science (Vol. 15, pp. 203–209). Department of Artificial Intelligence and Software Engineering, Complutense University, 28040 Madrid, Spain: Elsevier. https://doi.org/10.1016/j.procs.2012.10.072 | 3. Technological training that is not focus on improving reading |
| Shabalina, O., Voronina, A., Davtian, A., Delekelver, J., Peeters, E., & Hensbergen, R. (2018). A Mobile Game for Training Shopping Skills for People With Intellectual Disabilities. In Ciussi, M (Ed.), PROCEEDINGS OF THE 12TH EUROPEAN CONFERENCE ON GAMES BASED LEARNING (ECGBL 2018) (pp. 565–573). | 8. Training oriented to special population (other than dyslexia) |
| Shafiq, S., & Khan, T. A. (2018). Role & value of usability in educational learning via game based apps. International Journal of Scientific and Technology Research, 7(11), 70–77. https://www.scopus.com/inward/record.uri?eid=2-s2.0-85059848798&partnerID=40&md5=37f312afc96077eab6e4c8549b5cb9d8 | 12. Other: No access granted, same data… |
| Shamir, H., & Johnson, E. P. (2012). The effectiveness of computer-based EFL instruction among primary school students in Israel. Educational Media International, 49(1), 49–61. https://doi.org/10.1080/09523987.2012.662624 | 9. Training oriented to a second-language learning |
| Shamir, H., Crowther, M., & Sirrine, C. (2008). Comparing computer-based instruction, preschool instruction, and supplemantry reading material. In Proceedings of the 10th IASTED International Conference on Computers and Advanced Technology in Education (pp. 28–32). Waterford Research Insitute, 55 West 900 South, Salt Lake City, UT 84101, United States. Retrieved from https://www.scopus.com/inward/record.uri?eid=2-s2.0-62449298056&partnerID=40&md5=16b4c6e048d1def7b6252099cb1a59b4 | 9. Training oriented to a second-language learning |
| Shamsuddin, S. N. W., Mat, N. S. F. N., & Makhtar, M. (2017). RELEVANT TEST SET USING FEATURE SELECTION ALGORITHM FOR EARLY DETECTION OF DYSLEXIA. JOURNAL OF FUNDAMENTAL AND APPLIED SCIENCES, 9(6, SI), 886–899. https://doi.org/10.4314/jfas.v9i6s.66 | 2. It is a reading-related technology but it is not a training (test, support…) |
| Sharma, S. (2019). Smartphone based language learning through mobile apps. International Journal of Recent Technology and Engineering, 8(4), 8040–8043. https://doi.org/10.35940/ijrte.D6783.118419 | 9. Training oriented to a second-language learning |
| Shawar, M. (2008). The effect of using computer-based educational games on developing the reading comprehension and attitudes of sixth grade EFL students in Amman fourth directorate towards reading. Retrieved from http://ezproxy.si.unav.es:2048/login?url=http://search.ebscohost.com/login.aspx?direct=true&AuthType=ip,url&db=edsshm&AN=edsshm.24660&lang=es&site=eds-live&scope=site | 9. Training oriented to a second-language learning |
| Shelton, B. E., Neville, D., & McInnis, B. (2008). Cybertext redux: Using interactive fiction to teach german vocabulary, reading, and culture. In Computer-Supported Collaborative Learning Conference, CSCL (pp. 128–129). Utah State University, 2830 Old Main Hill, Logan, UT 84322, United States. Retrieved from https://www.scopus.com/inward/record.uri?eid=2-s2.0-84880410387&partnerID=40&md5=89c80646aed691dab849dbcfacb10140 | 9. Training oriented to a second-language learning |
| Shin, H., Gil, Y.-H., Yu, C., Kim, H.-K., Lee, J., & Jee, H.-K. (2017). Improved and Accessible E-book Reader Application for Visually Impaired People. SIGGRAPH ASIA 2017 POSTERS (SA’17). https://doi.org/10.1145/3145690.3145748 | 8. Training oriented to special population (other than dyslexia) |
| Shudong Wang, Hong Jiao, Young, M. J., Brooks, T., & Olson, J. (2008). Comparability of Computer-Based and Paper-and-Pencil Testing in K–12 Reading Assessments. Educational and Psychological Measurement. https://doi.org/10.1177/0013164407305592 | 2. It is a reading-related technology but it is not a training (test, support…) |
| Shukla, S., Shivakumar, A., Vasoya, M., Pei, Y., & Lyon, A. F. (2019). iLEAP: A human-AI teaming based mobile language learning solution for dual language learners in early and special educations. In I. P. O. G. S. I. A. Ravesteijn P. Rodrigues L. (Ed.), Proceedings of the 15th International Conference on Mobile Learning 2019, ML 2019 (pp. 57–64). IADIS Press. https://www.scopus.com/inward/record.uri?eid=2-s2.0-85065876803&partnerID=40&md5=0be0da8b9342129b755ed464c45f28eb | 9. Training oriented to a second-language learning |
| Singleton, C. (2012). Visual Stress and its Relationship to Dyslexia. Visual Aspects of Dyslexia. Oxford University Press. https://doi.org/10.1093/acprof:oso/9780199589814.003.0006 | 4. Reading-related but it is not neither a training or technological |
| Singleton, C., Horne, J., & Simmons, F. (2009). Computerised screening for dyslexia in adults. Journal of Research in Reading, 32(1), 137–152. https://doi.org/10.1111/j.1467-9817.2008.01386.x | 2. It is a reading-related technology but it is not a training (test, support…) |
| Sisco, H. T. (2008). A correlation of technology implementation and middle school academic achievement in Tennessee’s middle schools. Dissertation Abstracts International Section A: Humanities and Social Sciences. ProQuest Information & Learning, US. Retrieved from http://ezproxy.usherbrooke.ca/login?url=https://search.ebscohost.com/login.aspx?direct=true&db=psyh&AN=2008-99190-465&site=ehost-live | 3. Technological training that is not focus on improving reading |
| Siti Suhaila, A. H., Novia, A., Azrina, K., Abdul Hamid, S. S., Admodisastro, N., & Kamaruddin, A. (2016). A study of computer-based learning model for students with dyslexia. 2015 9th Malaysian Software Engineering Conference, MySEC 2015, 284–289. https://doi.org/10.1109/MySEC.2015.7475234 | 2. It is a reading-related technology but it is not a training (test, support…) |
| Skiada, R., Soroniati, E., Gardeli, A., & Zissis, D. (2014). EasyLexia: A Mobile Application for Children with Learning Difficulties. Procedia Computer Science, 27(Dsai 2013), 218–228. https://doi.org/10.1016/j.procs.2014.02.025 | 7. Technological reading training that do not fulfill methods requirements |
| Smeets, D. J. H., & Bus, A. G. (2012). Interactive electronic storybooks for kindergartners to promote vocabulary growth. Journal of Experimental Child Psychology, 112(1), 36–55. https://doi.org/10.1016/j.jecp.2011.12.003 | 2. It is a reading-related technology but it is not a training (test, support…) |
| Snoussi, S. (2018). Smartphone Arabic Signboards Images Reading. 2nd IEEE International Workshop on Arabic and Derived Script Analysis and Recognition, ASAR 2018, 52–56. https://doi.org/10.1109/ASAR.2018.8480171 | 5. Technology but it is not neither a training or reading-related |
| Snyder, C. K. C. (2013). Effects of training on early childhood special education paraeducators’ use of early literacy strategies during book reading. Dissertation Abstracts International Section A: Humanities and Social Sciences. ProQuest Information & Learning, US. Retrieved from http://search.proquest.com/docview/1492669390?accountid=14182$%5C$nhttp://sfxhostedeu.exlibrisgroup.com/44SUS/?url%7B_%7Dver=Z39.88-2004%7B&%7Drft%7B_%7Dval%7B_%7Dfmt=info:ofi/fmt:kev:mtx:dissertation%7B&%7Dgenre=dissertations+%7B&%7D+theses%7B&%7Dsid=Pro | 1.Non technological reading training |
| Sokal, L. (2010). Long-term effects of male reading tutors, choice of text and computer-based text on boys’ reading achievement. Language and Literacy, 12(1), 116–127. Retrieved from http://ezproxy.si.unav.es:2048/login?url=http://search.ebscohost.com/login.aspx?direct=true&AuthType=ip,url&db=edo&AN=55317444&lang=es&site=eds-live&scope=site | 2. It is a reading-related technology but it is not a training (test, support…) |
| Sokal, L., & Katz, H. (2008). Effects of technology and male teachers on boys’ reading. Australian Journal of Education (ACER Press), 52(1), 81–94. https://doi.org/10.1177/000494410805200106 |  |
| Spektor-Levy, O. (2012). The Impact of Learning with Laptops in 1:1 Classes on the Development of Learning Skills and Information Literacy among Middle School Students. Interdisciplinary Journal of E-Learning & Learning Objects, 8, 83–96. Retrieved from http://ezproxy.si.unav.es:2048/login?url=http://search.ebscohost.com/login.aspx?direct=true&AuthType=ip,url&db=eric&AN=EJ1058333&lang=es&site=eds-live&scope=site | 2. It is a reading-related technology but it is not a training (test, support…) |
| Spezio, R. N. (2013). The impact of a neurofeedback program on primary grade reading scores of children with lead poisoning. Dissertation Abstracts International Section A: Humanities and Social Sciences. ProQuest Information & Learning, US. Retrieved from http://ovidsp.ovid.com/ovidweb.cgi?T=JS&CSC=Y&NEWS=N&PAGE=fulltext&D=psyc8&AN=2013-99010-088%5Cnhttp://link.kib.ki.se?sid=OVID:psycdb&id=pmid:&id=doi:&issn=0419-4209&isbn=9781267246141&volume=73&issue=7-A%28E%29&spage=No&pages=No+Pagination+Specified&date | 8. Training oriented to special population (other than dyslexia) |
| Springer, L. (2009). Computereinsatz in der Sprach-, Sprech- und Schriftsprachtherapie. Sprache · Stimme · Gehör, 33(04), 165–165. https://doi.org/10.1055/s-0029-1243219 | 2. It is a reading-related technology but it is not a training (test, support…) |
| Srivastava, P. (2010). Hypertext reading comprehension in adolescents with typical language development and language-learning disability. ProQuest Dissertations and Theses. ProQuest LLC, US. Retrieved from http://proxy.lib.uiowa.edu/login?url=http://search.proquest.com/docview/759016224?accountid=14663%5Cnhttp://infolink.lib.uiowa.edu/sfx_local?url_ver=Z39.88-2004&rft_val_fmt=info:ofi/fmt:kev:mtx:dissertation&genre=dissertations+%26+theses&sid=ProQ:ProQuest | 2. It is a reading-related technology but it is not a training (test, support…) |
| Srivastava, P., & Gray, S. (2012). Computer-Based and Paper-Based Reading Comprehension in Adolescents With Typical Language Development and Language-Learning Disabilities. Language Speech and Hearing Services in Schools, 43(4), 424. https://doi.org/10.1044/0161-1461(2012/10-0108) | 2. It is a reading-related technology but it is not a training (test, support…) |
| Steinkuehler, C., Compton-Lilly, C., & King, E. (2010). Reading in the context of online games. In 9th International Conference of the Learning Sciences (Vol. 1, pp. 222–229). University of Wisconsin-Madison, 225 North Mills Street, Madison WI 53706, United States. Retrieved from https://www.scopus.com/inward/record.uri?eid=2-s2.0-84874142560&partnerID=40&md5=bfffa4c36293129fa1bb0e5918c5bb10 | 2. It is a reading-related technology but it is not a training (test, support…) |
| Stelzle, F., Ugrinovic, B., Knipfer, C., Bocklet, T., Nöth, E., Schuster, M., … Nkenke, E. (2010). Automatic, computer-based speech assessment on edentulous patients with and without complete dentures - preliminary results. Journal of Oral Rehabilitation, 37(3), 209–216. https://doi.org/10.1111/j.1365-2842.2009.02047.x | 5. Technology but it is not neither a training or reading-related |
| Sterling, T. M. (2013). The effect of reading test mode interchangeability and student assessment preferences on achievement. ProQuest Dissertations and Theses. ProQuest Information & Learning, US. Retrieved from http://proxy.lib.uiowa.edu/login?url=http://search.proquest.com/docview/1220695896?accountid=14663%5Cnhttp://infolink.lib.uiowa.edu/sfx_local?url_ver=Z39.88-2004&rft_val_fmt=info:ofi/fmt:kev:mtx:dissertation&genre=dissertations+&+theses&sid=ProQ:ProQuest+ | 2. It is a reading-related technology but it is not a training (test, support…) |
| Stone, B. (2011). Will “TiVos for Reading” Save Old Media? Bloomberg Businessweek, (4217), 40–42. Retrieved from http://ezproxy.si.unav.es:2048/login?url=http://search.ebscohost.com/login.aspx?direct=true&AuthType=ip,url&db=bth&AN=58665038&lang=es&site=eds-live&scope=site | 2. It is a reading-related technology but it is not a training (test, support…) |
| Štorková, P., & Kysela, J. (2015). Tablet as a New Interactive Tool for Education Paleography. Procedia - Social and Behavioral Sciences, 174(1), 3164–3169. https://doi.org/10.1016/j.sbspro.2015.01.1057 | 10. Educational-related but it is not about reading |
| Stoyanova, D., Stoyanova-Petrova, S., Kafadarova, N., & Mileva, N. (2016). Our Experience in Elaboration of a System of Exercises for Development of Self-Control By Using Mobile Technology. In Chova, LG and Martinez, AL and Torres, IC (Ed.), Edulearn16: 8Th International Conference on Education and New Learning Technologies (pp. 381–386). | 10. Educational-related but it is not about reading |
| Strømsø, H. I., & Bråten, I. (2010). Learning from multiple information sources. In International Encyclopedia of Education (pp. 191–196). University of Oslo, Oslo, Norway: Elsevier Ltd. https://doi.org/10.1016/B978-0-08-044894-7.00496-6 | 10. Educational-related but it is not about reading |
| Strong, G. K., Torgerson, C. J., Torgerson, D., & Hulme, C. (2011). A systematic meta-analytic review of evidence for the effectiveness of the “Fast ForWord” language intervention program. Journal of Child Psychology and Psychiatry, 52(3), 224–235. https://doi.org/10.1111/j.1469-7610.2010.02329.x | 11. Type of study excluded (review, non-primary references…) |
| Stufft, C. J. (2016). Tweens’ perceptions of literacy in relation to videogames: Figured worlds of literacy and gaming. Dissertation Abstracts International Section A: Humanities and Social Sciences. ProQuest Information & Learning, US. Retrieved from http://search.ebscohost.com/login.aspx?direct=true&db=psyh&AN=2016-31151-009&site=ehost-live | 5. Technology but it is not neither a training or reading-related |
| Suazo, F. I., & Graff, B. (2001). Using a Mobile Timed Reading Application to Improve Fluency in ELLs. In Chova, LG and Belenguer, DM and Martinez, AL (Ed.), EDULEARN11: 3RD INTERNATIONAL CONFERENCE ON EDUCATION AND NEW LEARNING TECHNOLOGIES (pp. 3524–3529). | 9. Training oriented to a second-language learning |
| Sudický, P., & Neničková, V. (2014). iPAD in academic settings : A pilot project. Applied Technologies & Innovations, 10(4), 141. https://doi.org/10.15208/ati.2014.21 | 10. Educational-related but it is not about reading |
| Supriyono, H., Adhantoro, M. S., & Rahmadzani, R. F. (2018). Developing Mobile Interactive Learning Media with Educational Game for Supporting Javanese Letters Learning. ADVANCED SCIENCE LETTERS, 24(12), 9173–9177. https://doi.org/10.1166/asl.2018.12119 | 3. Technological training that is not focus on improving reading |
| Szegletes, L., & Forstner, B. (2013). Reusable framework for the development of adaptive games. In 4th IEEE International Conference on Cognitive Infocommunications, CogInfoCom 2013 - Proceedings (pp. 601–606). https://doi.org/10.1109/CogInfoCom.2013.6719173 | 5. Technology but it is not neither a training or reading-related |
| Szelag, E., Dacewicz, A., Szymaszek, A., Wolak, T., Senderski, A., Domitrz, I., & Oron, A. (2015). The application of timing in therapy of children and adults with language disorders. Frontiers in Psychology, 6(NOV). https://doi.org/10.3389/fpsyg.2015.01714 | 8. Training oriented to special population (other than dyslexia) |
| Tabassum, K. (2020). Using wireless and mobile technologies to enhance teaching and learning strategies. Indonesian Journal of Electrical Engineering and Computer Science, 17(3), 1555–1561. https://doi.org/10.11591/ijeecs.v17.i3.pp1555-1561 | 11. Type of study excluded (review, non-primary references…) |
| Tabuenca, B., Kalz, M., Ternier, S., & Specht, M. (2015). Stop and think: Exploring mobile notifications to foster reflective practice on meta-learning. IEEE Transactions on Learning Technologies, 8(1), 124–135. https://doi.org/10.1109/TLT.2014.2383611 | 3. Technological training that is not focus on improving reading |
| Taj, I. H., Ali, F., Sipra, M. A., & Ahmad, W. (2017). Effect of Technology Enhanced Language Learning on EFL Reading Comprehension at Tertiary Level. ARAB WORLD ENGLISH JOURNAL, 8(1), 108–129. https://doi.org/10.24093/awej/vol8no1.9 | 9. Training oriented to a second-language learning |
| Tan, C.-C., Chen, C.-M., & Lee, H.-M. (2013). Using a Paper-based Digital Pen for Supporting English Courses in Regular Classrooms to Improve Reading Fluency. International Journal of Humanities and Arts Computing, 7(supplement), 234–246. https://doi.org/10.3366/ijhac.2013.0073 | 9. Training oriented to a second-language learning |
| Tan, J., Nguyen, C.-T., & Wang, X. (2017). SilentTalk: Lip reading through ultrasonic sensing on mobile phones. Proceedings - IEEE INFOCOM. https://doi.org/10.1109/INFOCOM.2017.8057099 | 2. It is a reading-related technology but it is not a training (test, support…) |
| Tan, W. K., Wang, S. J., & Janet, J. (2017). Pairing craft-making with mandarin eBooks: An investigation into the potential use of craft for language learning by preschoolers. (B. A.L. & B. E., Eds.), Lecture Notes of the Institute for Computer Sciences, Social-Informatics and Telecommunications Engineering, LNICST. Department of Design, Faculty of Art Design & Architecture, Monash University, Melbourne, VIC, Australia: Springer Verlag. https://doi.org/10.1007/978-3-319-55834-9_11 | 1.Non technological reading training |
| Tan, W.-K., Wang, S. J., & Janet, J. (2017). Pairing craft-making with mandarin eBooks: An investigation into the potential use of craft for language learning by preschoolers. Lecture Notes of the Institute for Computer Sciences, Social-Informatics and Telecommunications Engineering, LNICST, 196, 97–104. https://doi.org/10.1007/978-3-319-55834-9_11 | 1.Non technological reading training |
| Tan, W.-K., Wang, S. J., & Janet, J. (2018). Pairing Craft-Making with Mandarin eBooks: An Investigation into the Potential Use of Craft for Language Learning by Preschoolers. In Brooks, AL and Brooks, E (Ed.), INTERACTIVITY, GAME CREATION, DESIGN, LEARNING, AND INNOVATION (Vol. 196, pp. 97–104). https://doi.org/10.1007/978-3-319-55834-9_11 | 4. Reading-related but it is not neither a training or technological |
| Tanaka, K., Hasegawa, K., Makino, Y., & Shinoda, H. (2016). A pocket-size alphabet display with letter trajectories presented to fingers. In Bello, F and Kajimoto, H and Visell, Y (Ed.), Lecture Notes in Computer Science (including subseries Lecture Notes in Artificial Intelligence and Lecture Notes in Bioinformatics) (Vol. 9774, pp. 472–482). https://doi.org/10.1007/978-3-319-42321-0_44 | 2. It is a reading-related technology but it is not a training (test, support…) |
| Tandika, P. B., & Ndijuye, L. G. (2019). Pre-primary teachers’ preparedness in integrating information and communication technology in teaching and learning in Tanzania. INFORMATION AND LEARNING SCIENCES, 121(1/2), 79–94. https://doi.org/10.1108/ILS-01-2019-0009 | 10. Educational-related but it is not about reading |
| Tang, J., Li, R., Han, H., Zhang, H., & Gu, X. (2017). Detecting Permission Over-claim of Android Applications with Static and Semantic Analysis Approach. 2017 16TH IEEE INTERNATIONAL CONFERENCE ON TRUST, SECURITY AND PRIVACY IN COMPUTING AND COMMUNICATIONS / 11TH IEEE INTERNATIONAL CONFERENCE ON BIG DATA SCIENCE AND ENGINEERING / 14TH IEEE INTERNATIONAL CONFERENCE ON EMBEDDED SOFTWARE AND SYSTEMS, 706–713. https://doi.org/10.1109/Trustcom/BigDataSE/ICESS.2017.303 | 5. Technology but it is not neither a training or reading-related |
| Tang, J., Li, R., Han, H., Zhang, H., & Gu, X. (2017). Detecting Permission Over-claim of Android Applications with Static and Semantic Analysis Approach. 2017 16TH IEEE INTERNATIONAL CONFERENCE ON TRUST, SECURITY AND PRIVACY IN COMPUTING AND COMMUNICATIONS / 11TH IEEE INTERNATIONAL CONFERENCE ON BIG DATA SCIENCE AND ENGINEERING / 14TH IEEE INTERNATIONAL CONFERENCE ON EMBEDDED SOFTWARE AND SYSTEMS, 706–713. https://doi.org/10.1109/Trustcom/BigDataSE/ICESS.2017.303 | 5. Technology but it is not neither a training or reading-related |
| Taningco, M. T. V., & Pachon, H. P. (2008). Computer Use, Parental Expectations, & Latino Academic Achievement. Tomas Rivera Policy Institute. Tomas Rivera Policy Institute. Retrieved from http://eric.ed.gov/?id=ED502054 | 3. Technological training that is not focus on improving reading |
| Tapia, R. Á., Martín-Rodilla, P., & Oliva, Á. (2013). Towards a social reader. In GarciaPenalvo, FJ (Ed.), Proceedings of the First International Conference on Technological Ecosystem for Enhancing Multiculturality - TEEM ’13 (pp. 381–384). https://doi.org/10.1145/2536536.2536594 | 2. It is a reading-related technology but it is not a training (test, support…) |
| Tariq, R., & Latif, S. (2015). Designing a learning aid to assit to the dyslexic children with writing difficulties. In I. Chova, LG and Martinez, AL and Torres (Ed.), INTED2015: 9TH INTERNATIONAL TECHNOLOGY, EDUCATION AND DEVELOPMENT CONFERENCE (pp. 6502–6511). | 3. Technological training that is not focus on improving reading |
| Tariq, R., & Latif, S. (2016). A mobile application to improve Learning Performance of Dyslexic Children with Writing Difficulies. Journal of Educational Technology & Society VO - 19, 19(4), 151–166. Retrieved from http://ezproxy.si.unav.es:2048/login?url=http://search.ebscohost.com/login.aspx?direct=true&AuthType=ip,url&db=edsjsr&AN=edsjsr.jeductechsoci.19.4.151&lang=es&site=eds-live&scope=site | 3. Technological training that is not focus on improving reading |
| Taylor, N., & Ayorkor Korsah, G. (2018). Technology-based tutoring to improve second language literacy among children: Proof-of-concept study in Rural Ghana. IEEE International Conference on Adaptive Science and Technology, ICAST, 2018-Augus. https://doi.org/10.1109/ICASTECH.2018.8507123 | 9. Training oriented to a second-language learning |
| Tenemaza, M., Navarrete, R., Jaramillo, E., & Rodriguez, A. (2019). Specific dyslexia exploratory test (TEDE): Two tasks using augmented reality. Advances in Intelligent Systems and Computing, 794, 925–933. https://doi.org/10.1007/978-3-319-94947-5_91 | 2. It is a reading-related technology but it is not a training (test, support…) |
| Thelijjagoda, S., Chandrasiri, M., Hewathudalla, D., Ranasinghe, P., & Wickramanayake, I. (2019). The hope: An interactive mobile solution to overcome the writing, reading and speaking weaknesses of dyslexia. 14th International Conference on Computer Science and Education, ICCSE 2019, 808–813. https://doi.org/10.1109/ICCSE.2019.8845396 | 2. It is a reading-related technology but it is not a training (test, support…) |
| Thi Hien, V. T., Murali, G., Linh, N. K., Yen, N. H., Hien, N. T. T., Abuzied, A. S., Chen, Z., Ma, L., & Wang, L. (2018). Designing an application for learning Chinese. Lecture Notes in Computer Science (Including Subseries Lecture Notes in Artificial Intelligence and Lecture Notes in Bioinformatics), 10912 LNCS, 80–94. https://doi.org/10.1007/978-3-319-92252-2_7 | 7. Technological reading training that do not fulfill methods requirements |
| Tian, Z., Wang, L.-Z., Hu, Y.-H., Zhang, C.-Y., & Li, J. (2017). Improving the teaching quality by multiple tools and technology in oral histopathology experimental course. Shanghai Kou Qiang Yi Xue = Shanghai Journal of Stomatology, 26(2), 237–240. https://www.scopus.com/inward/record.uri?eid=2-s2.0-85045864957&partnerID=40&md5=e2a3bb5c130d092cc5a16d0188816f62 | 10. Educational-related but it is not about reading |
| Tijms, J., Pavlidou, E. V., & Hoette, H. A. I. (2020). Improvements in reading and spelling skills after a phonological and morphological knowledge intervention in Greek children with spelling difficulties: a pilot study. European Journal of Special Needs Education. https://doi.org/10.1080/08856257.2019.1709702 | 7. Technological reading training that do not fulfill methods requirements |
| Tingir, S., Cavlazoglu, B., Caliskan, O., Koklu, O., & Intepe-Tingir, S. (2017). Effects of mobile devices on K-12 students’ achievement: a meta-analysis. JOURNAL OF COMPUTER ASSISTED LEARNING, 33(4), 355–369. https://doi.org/10.1111/jcal.12184 | 11. Type of study excluded (review, non-primary references…) |
| Tirmizi, S. A. U., Iftikhar, Y., Ali, S., Ehsan, A., Ehsan, A., & Shahid, S. (2019). Ustaad: A mobile platform for teaching illiterates. Lecture Notes in Computer Science (Including Subseries Lecture Notes in Artificial Intelligence and Lecture Notes in Bioinformatics), 11747 LNCS, 788–796. https://doi.org/10.1007/978-3-030-29384-0_47 | 2. It is a reading-related technology but it is not a training (test, support…) |
| Torres-Toukoumidis, A., Romero-Rodríguez, L., Pérez-Rodríguez, M. A., & Björk, S. (2016). Development of reading skills through video games: State of the art. Ocnos, 15(2), 37-49. https://doi.org/10.18239/ocnos-2016.15.2.1124 | 11. Type of study excluded (review, non-primary references…) |
| Tyler, E. J. (2013). Improving the reading skills of typically developing children and children with an intellectual disability. Bangor University. Retrieved from https://search.proquest.com/docview/1779545061?accountid=14680%0Ahttp://openurl.ac.uk/?url_ver=Z39.88-2004&rft_val_fmt=info:ofi/fmt:kev:mtx:dissertation&genre=dissertations+%26+theses&sid=ProQ:ProQuest+Dissertations+%26+Theses+Global&atitle=&title=Improvi | 8. Training oriented to special population (other than dyslexia) |
| Tyler, E. J., Hughes, J. C., Beverley, M., & Hastings, R. P. (2015). Improving early reading skills for beginning readers using an online programme as supplementary instruction. European Journal of Psychology of Education, 30(3), 281–294. https://doi.org/10.1007/s10212-014-0240-7 | 8. Training oriented to special population (other than dyslexia) |
| U.S. Department of Education. (2009). Accelerated Reader. What Works Clearinghouse Intervention Report. What Works Clearinghouse. | 11. Type of study excluded (review, non-primary references…) |
| U.S. Department of Education. (2010). Herman Method [TM]. What Works Clearinghouse Intervention Report. What Works Clearinghouse. | 11. Type of study excluded (review, non-primary references…) |
| U.S. Department of Education. (2012). Success for All. What Works Clearinghouse Intervention Report. What Works Clearinghouse. | 11. Type of study excluded (review, non-primary references…) |
| Valeeva, N. G., Pavlova, E. B., & Zakirova, Y. L. (2019). M-learning in Teaching ESP: Case Study of Ecology Students. EUROPEAN JOURNAL OF CONTEMPORARY EDUCATION, 8(4), 920–930. https://doi.org/10.13187/ejced.2019.4.920 | 10. Educational-related but it is not about reading |
| Valero, A. L., Fern??ndez, E. E., & Mart??nez, I. J. (2011). Competencia digital y literacidad: Nuevos formatos narrativos en el videojuego ??dragon Age: Or??genes?? Comunicar, 18(36), 165–171. https://doi.org/10.3916/C36-2011-03-08 | 5. Technology but it is not neither a training or reading-related |
| Van Laere, E., Rosiers, K., Van Avermaet, P., Slembrouck, S., & van Braak, J. (2017). What can technology offer to linguistically diverse classrooms? Using multilingual content in a computer-based learning environment for primary education. JOURNAL OF MULTILINGUAL AND MULTICULTURAL DEVELOPMENT, 38(2), 97–112. https://doi.org/10.1080/01434632.2016.1171871 | 2. It is a reading-related technology but it is not a training (test, support…) |
| van Staden, A., & le Roux, N. A. (2010). The Efficacy of Fingerspell Coding and Visual Imaging Techniques in Improving the Spelling Proficiency of Deaf Signing Elementary-Phase Children: A South African Case Study. Journal of Developmental and Physical Disabilities, 22(6), 581–594. https://doi.org/10.1007/s10882-010-9196-y | 8. Training oriented to special population (other than dyslexia) |
| Vangeli, P., & Stage, J. (2018). Literature Survey on Interaction Design and Existing Software Applications for Dyslectic Users. In Auer, ME and Tsiatsos, T (Ed.), INTERACTIVE MOBILE COMMUNICATION TECHNOLOGIES AND LEARNING (Vol. 725, pp. 331–344). https://doi.org/10.1007/978-3-319-75175-7_34 | 2. It is a reading-related technology but it is not a training (test, support…) |
| Vangeli, P., & Stage, J. (2018). Literature Survey on Interaction Design and Existing Software Applications for Dyslectic Users. In Auer, ME and Tsiatsos, T (Ed.), INTERACTIVE MOBILE COMMUNICATION TECHNOLOGIES AND LEARNING (Vol. 725, pp. 331–344). https://doi.org/10.1007/978-3-319-75175-7_34 | 11. Type of study excluded (review, non-primary references…) |
| Vangeli, P., & Stage, J. (2018). Participatory design with dyslectics: Design and evaluation of an enhancing reading skills tool. Advances in Intelligent Systems and Computing, 725, 317–330. https://doi.org/10.1007/978-3-319-75175-7_33 | 2. It is a reading-related technology but it is not a training (test, support…) |
| Vassileva J. Ochoa S.F., I. T. G. C. (Ed.). (2017). 23rd International Conference on Collaboration Technologies, CRIWG 2017. Lecture Notes in Computer Science (Including Subseries Lecture Notes in Artificial Intelligence and Lecture Notes in Bioinformatics), 10391 LNCS, 1–259. https://www.scopus.com/inward/record.uri?eid=2-s2.0-85026206831&partnerID=40&md5=f371a7d24dabc6e23c2e1c443b257ba0 | 11. Type of study excluded (review, non-primary references…) |
| Vernucci, S., Canet-Juric, L., Andrés, M. L., & Burin, D. I. (2017). Reading comprehension and mathematical computation: The role of working memory in school-age children [Comprensión lectora y cálculo matemático: El rol de la memoria de trabajo en niños de edad escolar]. Psykhe, 26(2). https://doi.org/10.7764/psykhe.26.2.1047 | 4. Reading-related but it is not neither a training or technological |
| Versfeld, E., Foster, J., & Kuttel, M. (2015). Comparison of effectiveness of two mobile application designs for encouraging children to read. In C. L., K. D.G., W. B.W., B. R.J., & le R. D.B. (Eds.), Proceedings of the 2015 Annual Research Conference on South African Institute of Computer Scientists and Information Technologists - SAICSIT ’15 (Vol. 28-30-Sept, pp. 1–6). Department of Computer Science, University of Cape Town, Private Bag X3, Rondebosch, South Africa: Association for Computing Machinery. https://doi.org/10.1145/2815782.2815796 | 7. Technological reading training that do not fulfill methods requirements |
| Villányi, D., Martin, R., Sonnleitner, P., Siry, C., Fischbach, A., Villanyi, D., Martin, R., Sonnleitner, P., Siry, C., & Fischbach, A. (2018). A tablet-computer-based tool to facilitate accurate self-assessments in third- and fourth-graders. International Journal of Emerging Technologies in Learning, 13(10), 225–251. https://doi.org/10.3991/ijet.v13i10.8876 | 3. Technological training that is not focus on improving reading |
| Vullamparthi, A. J., Nelaturu, S. C. B., Mallaya, D. D., & Chandrasekhar, S. (2013). Assistive learning for children with autism using augmented reality. In Proceedings - 2013 IEEE 5th International Conference on Technology for Education, T4E 2013 (pp. 43–46). Centre for Development of Advanced Computing(C-DAC), Bangalore, India: IEEE Computer Society. https://doi.org/10.1109/T4E.2013.18 | 8. Training oriented to special population (other than dyslexia) |
| Vurdien, R. (2017). Mobile Assisted Vocabulary Acquisition and Wikis to Enhance Writing Skills. International Journal of Computer-Assisted Language Learning and Teaching, 7(2), 1–21. https://doi.org/10.4018/IJCALLT.2017040101 | 3. Technological training that is not focus on improving reading |
| Wade, E., Boon, R. T., & Spencer, V. G. (2010). Use of Kidspiration[C] Software to Enhance the Reading Comprehension of Story Grammar Components for Elementary-Age Students with Specific Learning Disabilities. Learning Disabilities: A Contemporary Journal, 8(2), 31–41. Retrieved from http://search.proquest.com/docview/854554445/1410F2A07886E2BC5B/60?accountid=14026#%5Cnhttp://search.proquest.com/docview/854554445/1410F2A07886E2BC5B/60?accountid=14026## | 3. Technological training that is not focus on improving reading |
| Walles, R. L. (2009). The road to mathematics in elementary school: Social and cognitive influences on performance and response to intervention 1193. Dissertation Abstracts International: Section B: The Sciences and Engineering. ProQuest Information & Learning, US. Retrieved from http://ezproxy.si.unav.es:2048/login?url=http://search.ebscohost.com/login.aspx?direct=true&AuthType=ip,url&db=psyh&AN=2009-99040-497&lang=es&site=eds-live&scope=site | 6. Training that is not neither technological or reading-related. |
| Walsh, M. (2017). MULTILITERACIES, MULTIMODALITY, NEW LITERACIES AND ... WHAT DO THESE MEAN FOR LITERACY EDUCATION? In Milton, M (Ed.), INCLUSIVE PRINCIPLES AND PRACTICES IN LITERACY EDUCATION (Vol. 11, pp. 19–33). https://doi.org/10.1108/S1479-363620170000011002 | 2. It is a reading-related technology but it is not a training (test, support…) |
| Walters, J. L. (2013). English language learners’ reading self-efficacy and achievement using 1:1 Mobile learning devices. Dissertation Abstracts International Section A: Humanities and Social Sciences. ProQuest Information & Learning, US. Retrieved from http://gateway.proquest.com/openurl?url_ver=Z39.88-2004&rft_val_fmt=info:ofi/fmt:kev:mtx:dissertation&res_dat=xri:pqm&rft_dat=xri:pqdiss:3503627%5Cnhttp://ovidsp.ovid.com/ovidweb.cgi?T=JS&PAGE=reference&D=psyc10&NEWS=N&AN=2013-99030-414 | 9. Training oriented to a second-language learning |
| Wang, I.-T., Wu, T.-T., Chien, Y.-C., & Huang, Y.-M. (2017). Integrating SQ4R and Student Team Achievement Division (STAD) Teaching Strategies with e-Books to Enhance Students’ English Reading Comprehension. In Lin, FT and Chen, SJ and Wang, DW and Chen, LJ (Ed.), PROCEEDINGS OF THE 2017 PACIFIC NEIGHBORHOOD CONSORTIUM ANNUAL CONFERENCE AND JOINT MEETINGS (PNC) (pp. 187–192). | 3. Technological training that is not focus on improving reading |
| Wang, N., Yu, K., Li, J., Zhang, R., & Ren, F. (2016). Readful-U. In Proceedings of the 2016 CHI Conference Extended Abstracts on Human Factors in Computing Systems - CHI EA ’16 (Vol. 07-12-May-, pp. 80–85). School of Information, University of Michigan, 105 S State, St. Ann Arbor, MI, United States: Association for Computing Machinery. https://doi.org/10.1145/2851581.2890639 | 2. It is a reading-related technology but it is not a training (test, support…) |
| Wang, X., Shi, M., & Li, C. (2019). Implementation of Elementary Chinese Language Learning Application in WeChat Mini Programs. 2019 4TH IEEE INTERNATIONAL CONFERENCE ON BIG DATA ANALYTICS (ICBDA 2019), 394–398. | 2. It is a reading-related technology but it is not a training (test, support…) |
| Wang, Y. H. (2014). Use of interactive web-based exercises for english as a foreign language learning: Learners’ perceptions. Teaching English with Technology, 14(3), 16–29. Retrieved from http://www.scopus.com/inward/record.url?eid=2-s2.0-84922803581&partnerID=40&md5=9845f8bb56f000aabc963bf8cfa4ea91 | 9. Training oriented to a second-language learning |
| Warner-Griffin, C., Liu, H., Tadler, C., Herget, D., Dalton, B., (ED), N. C. for E. S., & International, R. T. I. (2017). Reading Achievement of U.S. Fourth-Grade Students in an International Context: First Look at the Progress in International Reading Literacy Study (PIRLS) 2016 and ePIRLS 2016. NCES 2018-017. In National Center for Education Statistics. National Center for Education Statistics. https://ezproxy.unav.es/login?url=https://search.ebscohost.com/login.aspx?direct=true&AuthType=ip,url&db=eric&AN=ED578163&lang=es&site=eds-live&scope=site | 4. Reading-related but it is not neither a training or technological |
| Waseem Zia, M., & Ashfaq, N. (2013). Comparative Study of Children’s Usage of Media and Printed Material In Schools of Karachi. Pakistan Library & Information Science Journal, 44(1), 13–23. Retrieved from http://search.ebscohost.com/login.aspx?direct=true&db=lxh&AN=87672748&site=ehost-live | 5. Technology but it is not neither a training or reading-related |
| Wass, M., Lyxell, B., Sahlén, B., Asker-Árnason, L., Ibertsson, T., Mäki-Torkko, E., … Larsby, B. (2010). Reading strategies and cognitive skills in children with cochlear implants. Acta Neuropsychologica, 8(2), 142–180. Retrieved from http://www.embase.com/search/results?subaction=viewrecord%7B&%7Dfrom=export%7B&%7Did=L361298655%5Cnhttp://1035.indexcopernicus.com/fulltxt.php?ICID=932771%5Cnhttp://sfx.library.uu.nl/utrecht?sid=EMBASE%7B&%7Dissn=17307503%7B&%7Did=doi:%7B&%7Datitle=Readin | 8. Training oriented to special population (other than dyslexia) |
| Whitcomb, S. A., Bass, J. D., & Luiselli, J. K. (2011). Effects of a Computer-Based Early Reading Program (Headsprout??) on Word List and Text Reading Skills in a Student with Autism. Journal of Developmental and Physical Disabilities. https://doi.org/10.1007/s10882-011-9240-6 | 8. Training oriented to special population (other than dyslexia) |
| Whyte, E. M., Smyth, J. M., & Scherf, K. S. (2015). Designing Serious Game Interventions for Individuals with Autism. Journal of Autism and Developmental Disorders, 45(12), 3820–3831. https://doi.org/10.1007/s10803-014-2333-1 | 8. Training oriented to special population (other than dyslexia) |
| Winders, D., Logsdon, M. C., Vogt, K., Rushton, J., Myers, J., Lauf, A., & Hogan, F. (2017). Parent EDUCATION is CHANGING: A REVIEW OF SMARTPHONE APPS. MCN-THE AMERICAN JOURNAL OF MATERNAL-CHILD NURSING, 42(5), 248–256. https://doi.org/10.1097/NMC.0000000000000353 | 11. Type of study excluded (review, non-primary references…) |
| Wirawan, C., Qingyao, H., Yi, L., Yean, S., Lee, B.-S., & Ran, F. (2018). Pholder: An Eye-Gaze Assisted Reading Application on Android. In N. N. Y. K. G. L. Dipanda A. Chbeir R. (Ed.), Proceedings - 13th International Conference on Signal-Image Technology and Internet-Based Systems, SITIS 2017 (Vols. 2018-Janua, pp. 350–353). Institute of Electrical and Electronics Engineers Inc. https://doi.org/10.1109/SITIS.2017.64 | 2. It is a reading-related technology but it is not a training (test, support…) |
| Wirawan, C., Qingyao, H., Yi, L., Yean, S., Lee, B.-S., & Ran, F. (2018). Pholder: An Eye-Gaze Assisted Reading Application on Android. In N. N. Y. K. G. L. Dipanda A. Chbeir R. (Ed.), Proceedings - 13th International Conference on Signal-Image Technology and Internet-Based Systems, SITIS 2017 (Vols. 2018-Janua, pp. 350–353). Institute of Electrical and Electronics Engineers Inc. https://doi.org/10.1109/SITIS.2017.64 | 5. Technology but it is not neither a training or reading-related |
| Wołk, K., Wołk, A., & Glinkowski, W. (2017). A Cross-Lingual Mobile Medical Communication System Prototype for Foreigners and Subjects with Speech, Hearing, and Mental Disabilities Based on Pictograms. Computational and Mathematical Methods in Medicine, 2017. https://doi.org/10.1155/2017/4306416 | 8. Training oriented to special population (other than dyslexia) |
| Wołk, K., Wołk, A., & Marasek, K. (2017). Implementing statistical machine translation into mobile augmented reality systems. Advances in Intelligent Systems and Computing, 506, 61–73. https://doi.org/10.1007/978-3-319-43982-2_6 | 5. Technology but it is not neither a training or reading-related |
| Woodhead 2 ), Z. V. J. ( 1, Aguilar 4,5 ), O. M. ( 1, Leff 3,4 ), A. P. ( 1, Kerry, S. J. ( 3 ), Pappa, K. ( 3 ), Crinion, J. T. ( 3 ), Ong, Y.-H. Y.-H. Y.-H. ( 4 ), Hogan, J. S. ( 6 ), Woodhead, Z. V. J., Kerry, S. J. ( 3 ), Aguilar, O. M., Ong, Y.-H. Y.-H. Y.-H. ( 4 ), Hogan, J. S. ( 6 ), Pappa, K. ( 3 ), Leff, A. P., & Crinion, J. T. ( 3 ). (2018). Randomized trial of iReadMore word reading training and brain stimulation in central alexia. Brain, 141(7), 2127–2141. https://doi.org/10.1093/brain/awy138 | 8. Training oriented to special population (other than dyslexia) |
| Worrell, J. L. (2011). Effects of an interactive computer-based reading strategy on student comprehension [ProQuest LLC]. In Dissertation Abstracts International, A: The Humanities and Social Sciences. http://search.proquest.com/docview/1081898074?accountid=14548%255Cnhttp://metadata.lib.hku.hk/hku?url_ver=Z39.88-2004&rft_val_fmt=info:ofi/fmt:kev:mtx:dissertation&genre=dissertations+%2526+theses&sid=ProQ:Linguistics+and+Language+Behavior+Abstracts+%2528 | 2. It is a reading-related technology but it is not a training (test, support…) |
| Wu, J. (2015). Effects of CALL on Self-directed FL Vocabulary Learning. Studies in Self-Acess Learning Journal, 6(2), 191–215. | 9. Training oriented to a second-language learning |
| Xiao, Y., Liu, Y., & Hu, J. (2019). Regression Analysis of ICT Impact Factors on Early Adolescents’ Reading Proficiency in Five High-Performing Countries. FRONTIERS IN PSYCHOLOGY, 10. https://doi.org/10.3389/fpsyg.2019.01646 | 2. It is a reading-related technology but it is not a training (test, support…) |
| Xie, D. X., Wang, R. Y., & Chinnadurai, S. (2018). Readability of online patient education materials for velopharyngeal insufficiency. INTERNATIONAL JOURNAL OF PEDIATRIC OTORHINOLARYNGOLOGY, 104, 113–119. https://doi.org/10.1016/j.ijporl.2017.09.016 | 8. Training oriented to special population (other than dyslexia) |
| Xu, Z., Wijekumar, K. (Kay), Ramirez, G., Hu, X., & Irey, R. (2019). The effectiveness of intelligent tutoring systems on K-12 students’ reading comprehension: A meta-analysis. BRITISH JOURNAL OF EDUCATIONAL TECHNOLOGY, 50(6), 3119–3137. https://doi.org/10.1111/bjet.12758 | 11. Type of study excluded (review, non-primary references…) |
| Yagci, T. (2015). Blended Learning via Mobile Social Media &amp; Implementation of “EDMODO” in Reading Classes. Advances in Language and Literary Studies, 6(4), 41–47. https://doi.org/10.7575/aiac.alls.v.6n.4p.41 | 9. Training oriented to a second-language learning |
| Yamamoto, K., He, Q., Shin, H. J., & von Davier, M. (2017). Developing a Machine-Supported Coding System for Constructed-Response Items in PISA. Research Report. ETS RR-17-47. ETS Research Report Series. https://ezproxy.unav.es/login?url=https://search.ebscohost.com/login.aspx?direct=true&AuthType=ip,url&db=eric&AN=EJ1168681&lang=es&site=eds-live&scope=site | 2. It is a reading-related technology but it is not a training (test, support…) |
| Yang, H. L., Yang, Y., & Yue, Z. (2012). Research and Design of Electronic Speech Reader Based on Text-to-Speech Technology. In Zhang, CS (Ed.), Advanced Materials Research (Vols. 433–440, pp. 4883–4887). https://doi.org/10.4028/www.scientific.net/AMR.433-440.4883 | 2. It is a reading-related technology but it is not a training (test, support…) |
| Yanuardi, A. W., Johannes, A. P., & Alfeus, C. W. (2011). Multimedia sign language dictionary for the deaf and hard of hearing. 81–84. https://www.scopus.com/inward/record.uri?eid=2-s2.0-84255197925&partnerID=40&md5=e06a9c9c41df2836e980c449c36481e4 | 8. Training oriented to special population (other than dyslexia) |
| Yassin, S. Z., & Almegren, A. (2010). Out-Campus Students’ Perceptions & Attitudes Towards Reading English Materials Via Internet in the Esl Classroom. In Chova, LG and Belenguer, DM and Torres, IC (Ed.), 3Rd International Conference of Education, Research and Innovation (Iceri2010) (pp. 6286–6297). | 9. Training oriented to a second-language learning |
| Yaw, J. S., Skinner, C. H., Parkhurst, J., Taylor, C. M., Booher, J., & Chambers, K. (2011). Extending Research on a Computer-Based Sight-Word Reading Intervention to a Student with Autism. Journal of Behavioral Education, 20(1), 44–54. https://doi.org/10.1007/s10864-010-9118-1 | 8. Training oriented to special population (other than dyslexia) |
| Yaw, J., Skinner, C. H., Delisle, J., Skinner, A. L., Maurer, K., Cihak, D., Wilhoit, B., & Booher, J. (2014). Measurement scale influences in the evaluation of sight-word reading interventions. Journal of Applied Behavior Analysis, 47(2), 360–379. https://doi.org/10.1002/jaba.126 | 2. It is a reading-related technology but it is not a training (test, support…) |
| Yaw, J., Skinner, C. H., Orsega, M. C., Parkhurst, J., Booher, J., & Chambers, K. (2012). Evaluating a Computer-Based Sight-Word Reading Intervention in a Student With Intellectual Disabilities. Journal of Applied School Psychology, 28(4), 354–366. https://doi.org/10.1080/15377903.2012.722181 | 8. Training oriented to special population (other than dyslexia) |
| Yeh, C. F., Lee, H. Y., & Lee, L. S. (2013). Speaking rate normalization with lattice-based context-dependent phoneme duration modeling for personalized speech recognizers on mobile devices. In Bimbot, F and Cerisara, C and Fougeron, C and Gravier, G and Lamel, L and Pellegrino, F and Perrier, P (Ed.), Proceedings of the Annual Conference of the International Speech Communication Association, INTERSPEECH (pp. 1741–1745). | 2. It is a reading-related technology but it is not a training (test, support…) |
| Yeh, H.-Y., Tsai, Y.-T., & Chang, C.-K. (2017). Android app development for teaching reduced forms of EFL listening comprehension to decrease cognitive load. In Liu, J and Nishimura, S and Zhang, H and Jin, Q (Ed.), 2017 6TH INTERNATIONAL CONFERENCE OF EDUCATIONAL INNOVATION THROUGH TECHNOLOGY (EITT) (pp. 316–321). https://doi.org/10.1109/EITT.2017.82 | 9. Training oriented to a second-language learning |
| Yeh, M. K. C., Toshtzar, A., Guertin, L., & Yan, Y. (2016). Using spaced repetition and gamification to enhance K-12 student science literacy with on-demand mobile short reads. Proceedings - Frontiers in Education Conference, FIE, 2016-Novem. https://doi.org/10.1109/FIE.2016.7757361 | 3. Technological training that is not focus on improving reading |
| Yenpoeng, V., Krootjohn, S., & Sintanakul, K. (2018). CONCEPTUAL FRAMEWORK FOR DEVELOPMENT OF LEARNING ACTIVITY IN ORDER TO SUPPORT LINGUISTIC EXPERIENCE FOR KINDERGARTEN 2 STUDENTS. https://ezproxy.unav.es/login?url=https://search.ebscohost.com/login.aspx?direct=true&AuthType=ip,url&db=edsbas&AN=edsbas.9C30DAB6&lang=es&site=eds-live&scope=site | 3. Technological training that is not focus on improving reading |
| Yeo, K. J., & Lim, W. W. (2019). A systematic review of learning application for students with dyslexia. Indian Journal of Public Health Research and Development, 10(9), 1871–1874. https://doi.org/10.5958/0976-5506.2019.02727.X | 11. Type of study excluded (review, non-primary references…) |
| Ying, C., & Fu-Hong, Z. (2010). Study on anti-collision Q algorithm for UHF RFID. 2010 WRI International Conference on Communications and Mobile Computing, CMC 2010, 3, 168–170. https://doi.org/10.1109/CMC.2010.245 | 5. Technology but it is not neither a training or reading-related |
| Ying, L. (2015). Design of a Mobile Reading Room Application Program Under Android Platform. INTERNATIONAL CONFERENCE ON COMPUTER SCIENCE AND ENVIRONMENTAL ENGINEERING (CSEE 2015), 677–682. | 2. It is a reading-related technology but it is not a training (test, support…) |
| Yingchun, L. (2017). An Integration of Multiple Teaching Methods in the Compilation of College English Courses Based on New Technology. In Haskell, JA and Bo, H (Ed.), PROCEEDINGS OF THE 2017 NORTHEAST ASIA INTERNATIONAL SYMPOSIUM ON LINGUSTICS, LITERATURE AND TEACHING (NALLTS), VOLS A-C (pp. 602–607). | 2. It is a reading-related technology but it is not a training (test, support…) |
| Yokus, G., & Yelken, T. Y. (2017). The Adoption of Mobile Devices as Digital Tools for Seamless Learning. In DIGITAL TOOLS FOR SEAMLESS LEARNING (pp. 297–324). https://doi.org/10.4018/978-1-5225-1692-7.ch015 | 4. Reading-related but it is not neither a training or technological |
| Young, J. (2014). A study of print and computer-based reading to measure and compare rates of comprehension and retention. New Library World, 115(7/8), 376–393. https://doi.org/10.1108/NLW-05-2014-0051 | 2. It is a reading-related technology but it is not a training (test, support…) |
| YOUSEFZADEH, M. (2013). Effect of a Computer Software on Disabled Second Language Learners’Oral Reading Fluency. International Journal on New Trends in Education, 4(3), 21. http://www.ijonte.org/FileUpload/ks63207/File/ijonte_complete.pdf#page=28 | 9. Training oriented to a second-language learning |
| Yu, C. ., & Cheng, Y.-S. . (2012). The study of performance and satisfaction in acquiring credentials at workplace with the application of mobile learning through E-books. International Journal of Digital Content Technology and Its Applications, 6(1), 49–56. https://doi.org/10.4156/jdcta.vol6.issue1.7 | 2. It is a reading-related technology but it is not a training (test, support…) |
| Yu, C.-H., & Miller, R. C. (2011). Enhancing mobile browsing and reading. Proceedings of the 2011 Annual Conference Extended Abstracts on Human Factors in Computing Systems - CHI EA ’11, 1783. https://doi.org/10.1145/1979742.1979845 | 2. It is a reading-related technology but it is not a training (test, support…) |
| Yu, L. (2016). Interactive Research on WeChat Public Platform and Mobile Library Application in Colleges and Universities. In Yu, J and Liu, Q (Ed.), PROCEEDINGS OF THE 2016 4TH INTERNATIONAL CONFERENCE ON ELECTRICAL & ELECTRONICS ENGINEERING AND COMPUTER SCIENCE (ICEEECS 2016) (Vol. 50, pp. 1073–1076). | 2. It is a reading-related technology but it is not a training (test, support…) |
| Zagal, J. P., Tomuro, N., & Shepitsen, A. (2012). Natural Language Processing in Game Studies Research. Simulation & Gaming, 43(3), 356–373. https://doi.org/10.1177/1046878111422560 | 2. It is a reading-related technology but it is not a training (test, support…) |
| Zain, N. Z. M., Mahmud, M., & Hassan, A. (2013). Utilization of mobile apps among student with learning disability from Islamic perspective. 2013 5th International Conference on Information and Communication Technology for the Muslim World, ICT4M 2013. https://doi.org/10.1109/ICT4M.2013.6518889 | 2. It is a reading-related technology but it is not a training (test, support…) |
| Zain, Z. N. M. (2014). Understanding the needs and challenges of mobile applications for students with dyslexia. In Chova, LG and Martinez, AL and Torres, IC (Ed.), EDULEARN14: 6th International Conference on Education and New Learning Technologies (p. 3678). | 2. It is a reading-related technology but it is not a training (test, support…) |
| Zain, Z. N. M. (2014). Using English Learning Application in Mobile Environment To Support Learning of Dyslexic Students. In Chova, LG and Martinez, AL and Torres, IC (Ed.), Edulearn14: 6Th International Conference on Education and New Learning Technologies (p. 4388). | 9. Training oriented to a second-language learning |
| Zehner, F., Goldhammer, F., Lubaway, E., & Sälzer, C. (2019). Unattended consequences: how text responses alter alongside PISA’s mode change from 2012 to 2015. Education Inquiry, 10(1), 34–55. https://doi.org/10.1080/20004508.2018.1518080 | 10. Educational-related but it is not about reading |
| Zhang, J., Xie, M., Yuan, B., & Wang, M. (2019). Design and Implementation of Adaptive Push APP Based on Android for Fragmented English Reading Resources. Lecture Notes of the Institute for Computer Sciences, Social-Informatics and Telecommunications Engineering, LNICST, 299, 3–18. https://doi.org/10.1007/978-3-030-35095-6_1 | 10. Educational-related but it is not about reading |
| Zhou, N., & Yadav, A. (2017). Effects of multimedia story reading and questioning on preschoolers’ vocabulary learning, story comprehension and reading engagement. ETR&D-EDUCATIONAL TECHNOLOGY RESEARCH AND DEVELOPMENT, 65(6), 1523–1545. https://doi.org/10.1007/s11423-017-9533-2 | 2. It is a reading-related technology but it is not a training (test, support…) |
| Zhou, N., & Yadav, A. (2017). Effects of multimedia story reading and questioning on preschoolers’ vocabulary learning, story comprehension and reading engagement. In Educational Technology Research and Development (pp. 1–23). Springer New York LLC. https://doi.org/10.1007/s11423-017-9533-2 | 2. It is a reading-related technology but it is not a training (test, support…) |
| Zhou, N., & Yadav, A. (2017). Effects of multimedia story reading and questioning on preschoolers’ vocabulary learning, story comprehension and reading engagement. In Educational Technology Research and Development (pp. 1–23). Springer New York LLC. https://doi.org/10.1007/s11423-017-9533-2 | 2. It is a reading-related technology but it is not a training (test, support…) |
| Zulfiani, Z., Suwarna, I. P., & Miranto, S. (2018). SCIENCE EDUCATION ADAPTIVE LEARNING SYSTEM AS A COMPUTER-BASED SCIENCE LEARNING WITH LEARNING STYLE VARIATIONS. JOURNAL OF BALTIC SCIENCE EDUCATION, 17(4), 711–727. | 3. Technological training that is not focus on improving reading |
| Zurati, N., Abdullah, S. A. C., Zaman, F. H. K., Abidin, H. Z., & Soh, Z. H. C. (2019). Mobile App For Speed Reading Test In Bahasa Malaysia. 2019 IEEE 9TH SYMPOSIUM ON COMPUTER APPLICATIONS & INDUSTRIAL ELECTRONICS (ISCAIE), 193–197. | 2. It is a reading-related technology but it is not a training (test, support…) |
| Zurita, G., Baloian, N., Jerez, O., & Peñafiel, S. (2017). Practice of skills for reading comprehension in large classrooms by using a mobile collaborative support and microblogging. Lecture Notes in Computer Science (Including Subseries Lecture Notes in Artificial Intelligence and Lecture Notes in Bioinformatics), 10391 LNCS, 81–94. https://doi.org/10.1007/978-3-319-63874-4_7 | 2. It is a reading-related technology but it is not a training (test, support…) |
| Zurita, G., Baloian, N., Jerez, O., & Peñafiel, S. (2017). Practice of skills for reading comprehension in large classrooms by using a mobile collaborative support and microblogging: Vol. 10391 LNCS (V. J., O. S.F., I. T., & G. C. (eds.); pp. 81–94). Springer Verlag. https://doi.org/10.1007/978-3-319-63874-4_7 | 2. It is a reading-related technology but it is not a training (test, support…) |
| Zurita, G., Baloian, N., Peñafiel, S., & Jerez, O. (2019). Applying Pedagogical Usability for Designing a Mobile Learning Application that Support Reading Comprehension. Proceedings VO  - 31, 1, 6. https://doi.org/10.3390/proceedings2019031006 | 2. It is a reading-related technology but it is not a training (test, support…) |
| 孟志远, 谭维智, Zhi-yuan, M., & Wei-zhi, T. A. N. (2019). 移动终端进课堂:价值、挑战与应对策略 / Mobile Devices Entering Classroom: Values, Challenges and Countermeasures. 现代教育技术 / Modern Educational Technology, 2, 33. https://doi.org/10.3969/j.issn.1009-8097.2019.02.005 | 11. Type of study excluded (review, non-primary references…) |

# Supplementary table 2.1 Items of the PEDro scale

| 1 | Eligibility criteria were specified |
| --- | --- |
| 2 | Subjects were randomly allocated to groups (in a crossover study, subjects were andomly allocated an order in which treatments were received) |
| 3 | Allocation was concealed |
| 4 | The groups were similar at baseline regarding the most important prognostic indicators |
| 5 | There was blinding of all subjects |
| 6 | There was blinding of all therapists who administered the therapy |
| 7 | There was blinding of all assessors who measured at least one key outcome |
| 8 | Measures of at least one key outcome were obtained from more than 85% of the subjects initially allocated to groups |
| 9 | All subjects for whom outcome measures were available received the treatment or control condition as allocated or, where this was not the case, data for at least one key outcome was analysed by “intention to treat” |
| 10 | The results of between-group statistical comparisons are reported for at least one outcome |
| 11 | The study provides both point measures and measures of variability for at least one key outcome |

# Supplementary table 2.2. Risk of bias of included studies

Fulfillment of each item and final score of the PEDro scale for each of the studies finally included in the systematic review. 0-3, low risk of bias; 4-7, medium risk; 8-11, high risk. The description of each item of the PEDro scale can be found below the table

| ID | 1 | 2 | 3 | 4 | 5 | 6 | 7 | 8 | 9 | 10 | 11 | Total (risk of bias) |
| --- | --- | --- | --- | --- | --- | --- | --- | --- | --- | --- | --- | --- |
| Arvans 2010 | Yes | Yes | Yes | Yes | No | No | Yes | Yes | Yes | Yes | Yes | **9 (low)** |
| Beaundry 2016 | No | No | No | Yes | No | No | No | Yes | Yes | Yes | Yes | **5 (medium)** |
| Borman 2008 | Yes | Yes | Yes | Yes | No | No | Yes | No | Yes | Yes | Yes | **8 (low)** |
|  | Yes | Yes | Yes | Yes | No | No | Yes | No | Yes | Yes | Yes | **8 (low)** |
| De Primo 2016 | Yes | No | No | No | No | No | Yes | Yes | Yes | Yes | Yes | **6 (medium)** |
| Deault 2009 | Yes | Yes | Yes | Yes | No | No | Yes | Yes | Yes | Yes | Yes | **9 (low)** |
| Deshpande 2017 | Yes | Yes | No | Yes | No | No | No | Yes | Yes | Yes | Yes | **7 (medium)** |
| Ecalle 2009 | Yes | Yes | Yes | Yes | Yes | No | Yes | Yes | Yes | Yes | Yes | **6 (medium)** |
| Falke 2012 | Yes | No | Yes | No | No | No | Yes | Yes | Yes | Yes | Yes | **7 (medium)** |
| Given 2008 | Yes | Yes | Yes | Yes | No | No | Yes | Yes | Yes | Yes | Yes | **9 (low)** |
|  | Yes | Yes | Yes | Yes | No | No | Yes | Yes | Yes | Yes | Yes | **9 (low)** |
| Heikkila 2013 | Yes | Yes | Yes | No | No | No | Yes | Yes | Yes | Yes | Yes | **8 (low)** |
| Hill-Stephens 2013 | Yes | No | No | No | No | No | Yes | Yes | Yes | Yes | Yes | **6 (medium)** |
| Huffstetter 2010 | Yes | Yes | Yes | Yes | No | No | Yes | Yes | Yes | Yes | Yes | **9 (low)** |
| Jackson 2016 | Yes | No | No | No | No | No | Yes | Yes | Yes | Yes | Yes | **6 (medium)** |
| Jimenez 2010 | Yes | Yes | Yes | Yes | No | No | Yes | Yes | Yes | Yes | Yes | **9 (low)** |
|  | Yes | Yes | Yes | Yes | No | No | Yes | Yes | Yes | Yes | Yes | **9 (low)** |
|  | Yes | Yes | Yes | Yes | No | No | Yes | Yes | Yes | Yes | Yes | **9 (low)** |
| Jimenez 2008 | Yes | Yes | Yes | Yes | No | No | No | Yes | Yes | Yes | Yes | **8 (low)** |
| Kamykowska 2014 | Yes | Yes | Yes | Yes | No | No | Yes | Yes | Yes | Yes | Yes | **9 (low)** |
| Messer 2017 | Yes | Yes | No | Yes | No | No | No | No | No | Yes | Yes | **5 (medium)** |
| Moser 2017 | Yes | Yes | Yes | Yes | No | No | Yes | Yes | Yes | Yes | Yes | **9 (low)** |
| NeeChee 2017 | No | No | No | Yes | No | No | Yes | Yes | Yes | Yes | Yes | **6 (medium)** |
| OCallaghan 2016 | Yes | Yes | Yes | Yes | No | No | Yes | Yes | Yes | Yes | Yes | **9 (low)** |
| Pindiprolu 2009 | Yes | No | Yes | No | No | No | No | No | Yes | Yes | Yes | **5 (medium)** |
| Plony 2014 | Yes | No | No | Yes | No | No | Yes | Yes | Yes | Yes | Yes | **7 (medium)** |
| Ponce 2012 | Yes | No | No | No | No | No | No | Yes | Yes | Yes | Yes | **5 (medium)** |
| Ponce 2013 | Yes | Yes | No | Yes | No | No | No | No | Yes | Yes | Yes | **6 (medium)** |
| Rasinski 2011 | Yes | No | No | No | No | No | No | Yes | Yes | Yes | Yes | **5 (medium)** |
| Reed 2013 | Yes | No | No | No | No | No | No | Yes | Yes | Yes | Yes | **5 (medium)** |
|  | Yes | No | No | No | No | No | No | Yes | Yes | Yes | Yes | **5 (medium)** |
| Rello 2015 | Yes | Yes | Yes | Yes | No | No | Yes | Yes | Yes | Yes | Yes | **9 (low)** |
| Rogowsky 2011 | Yes | Yes | No | Yes | No | No | Yes | Yes | Yes | Yes | Yes | **8 (low)** |
| Rosas 2017 | Yes | Yes | Yes | Yes | No | No | Yes | Yes | Yes | Yes | Yes | **9 (low)** |
| Saine 2011 | Yes | Yes | Yes | Yes | No | No | Yes | Yes | Yes | Yes | Yes | **9 (low)** |
|  | Yes | Yes | Yes | Yes | No | No | Yes | Yes | Yes | Yes | Yes | **9 (low)** |
| Shannon 2015 | Yes | Yes | No | Yes | No | No | Yes | Yes | Yes | Yes | Yes | **8 (low)** |
| Soboleski 2011 | Yes | No | No | No | No | No | No | Yes | Yes | Yes | Yes | **5 (medium)** |
| Tijms 2011 | Yes | Yes | Yes | Yes | No | No | Yes | Yes | Yes | Yes | Yes | **9 (low)** |
| Watson 2008 | Yes | No | No | Yes | No | No | No | Yes | Yes | Yes | Yes | **6 (medium)** |
|  | Yes | No | No | Yes | No | No | No | Yes | Yes | Yes | Yes | **6 (medium)** |
| Williams 2012 | Yes | No | No | No | No | No | No | No | Yes | Yes | Yes | **4 (medium)** |
| Wolgemuth 2011 | Yes | No | No | Yes | No | No | No | No | Yes | Yes | Yes | **5 (medium)** |
| Wolgemuth 2013 | Yes | Yes | Yes | No | No | No | Yes | Yes | Yes | Yes | Yes | **8 (low)** |
| DiStasio 2012 | Yes | Yes | Yes | Yes | No | No | No | Yes | Yes | Yes | Yes | **8 (low)** |
| Franceschini 2013 | Yes | Yes | Yes | Yes | Yes | No | Yes | Yes | Yes | Yes | Yes | **10 (low)** |
| Maracuso 2008 | Yes | No | No | Yes | No | No | No | Yes | Yes | Yes | Yes | **6 (medium)** |
| Maracuso 2009 | Yes | Yes | No | Yes | No | No | No | Yes | Yes | Yes | Yes | **7 (medium)** |
| Maracuso 2011 | Yes | No | No | Yes | No | No | No | Yes | Yes | Yes | Yes | **6 (medium)** |
| McMurray 2013 | Yes | Yes | No | No | No | No | Yes | Yes | Yes | Yes | Yes | **7 (medium)** |
| Savage 2013 | Yes | Yes | Yes | Yes | No | No | Yes | Yes | Yes | Yes | Yes | **9 (low)** |
| Shelley-Tremblay 2009 | Yes | Yes | Yes | No | No | No | Yes | No | Yes | Yes | Yes | **7 (medium)** |
| Wild 2009 | Yes | Yes | Yes | Yes | No | No | Yes | Yes | Yes | Yes | Yes | **9 (low)** |
| Daleen 2018 | Yes | Yes | Yes | Yes | No | No | No | Yes | Yes | Yes | Yes | **9 (low)** |
| Flis 2018 | Yes | No | Yes | Yes | No | No | Yes | Yes | Yes | Yes | Yes | **9 (low)** |

# Supplementary table 3. PRISMA 2009 Checklist

| **Section/topic** | **#** | **Checklist item** |  |
| --- | --- | --- | --- |
| **TITLE** | | |  |
| Title | 1 | Identify the report as a systematic review, meta-analysis, or both. | Yes |
| **ABSTRACT** | | |  |
| Structured summary | 2 | Provide a structured summary including, as applicable: background; objectives; data sources; study eligibility criteria, participants, and interventions; study appraisal and synthesis methods; results; limitations; conclusions and implications of key findings; systematic review registration number. | Yes |
| **INTRODUCTION** | | |  |
| Rationale | 3 | Describe the rationale for the review in the context of what is already known. | Yes |
| Objectives | 4 | Provide an explicit statement of questions being addressed with reference to participants, interventions, comparisons, outcomes, and study design (PICOS). | Yes |
| **METHODS** | | |  |
| Protocol and registration | 5 | Indicate if a review protocol exists, if and where it can be accessed (e.g., Web address), and, if available, provide registration information including registration number. | Yes |
| Eligibility criteria | 6 | Specify study characteristics (e.g., PICOS, length of follow-up) and report characteristics (e.g., years considered, language, publication status) used as criteria for eligibility, giving rationale. | Yes |
| Information sources | 7 | Describe all information sources (e.g., databases with dates of coverage, contact with study authors to identify additional studies) in the search and date last searched. | Yes |
| Search | 8 | Present full electronic search strategy for at least one database, including any limits used, such that it could be repeated. | Yes |
| Study selection | 9 | State the process for selecting studies (i.e., screening, eligibility, included in systematic review, and, if applicable, included in the meta-analysis). | Yes |
| Data collection process | 10 | Describe method of data extraction from reports (e.g., piloted forms, independently, in duplicate) and any processes for obtaining and confirming data from investigators. | Yes |
| Data items | 11 | List and define all variables for which data were sought (e.g., PICOS, funding sources) and any assumptions and simplifications made. | Yes |
| Risk of bias in individual studies | 12 | Describe methods used for assessing risk of bias of individual studies (including specification of whether this was done at the study or outcome level), and how this information is to be used in any data synthesis. | Yes |
| Summary measures | 13 | State the principal summary measures (e.g., risk ratio, difference in means). | NA |
| Synthesis of results | 14 | Describe the methods of handling data and combining results of studies, if done, including measures of consistency (e.g., I^2^) for each meta-analysis. | NA |

| Risk of bias across studies | 15 | Specify any assessment of risk of bias that may affect the cumulative evidence (e.g., publication bias, selective reporting within studies). | NA |
| --- | --- | --- | --- |
| Additional analyses | 16 | Describe methods of additional analyses (e.g., sensitivity or subgroup analyses, meta-regression), if done, indicating which were pre-specified. | NA |
| **RESULTS** | | |  |
| Study selection | 17 | Give numbers of studies screened, assessed for eligibility, and included in the review, with reasons for exclusions at each stage, ideally with a flow diagram. | Yes |
| Study characteristics | 18 | For each study, present characteristics for which data were extracted (e.g., study size, PICOS, follow-up period) and provide the citations. | Yes |
| Risk of bias within studies | 19 | Present data on risk of bias of each study and, if available, any outcome level assessment (see item 12). | Yes |
| Results of individual studies | 20 | For all outcomes considered (benefits or harms), present, for each study: (a) simple summary data for each intervention group (b) effect estimates and confidence intervals, ideally with a forest plot. | NA |
| Synthesis of results | 21 | Present results of each meta-analysis done, including confidence intervals and measures of consistency. | NA |
| Risk of bias across studies | 22 | Present results of any assessment of risk of bias across studies (see Item 15). | NA |
| Additional analysis | 23 | Give results of additional analyses, if done (e.g., sensitivity or subgroup analyses, meta-regression [see Item 16]). | NA |
| **DISCUSSION** | | |  |
| Summary of evidence | 24 | Summarize the main findings including the strength of evidence for each main outcome; consider their relevance to key groups (e.g., healthcare providers, users, and policy makers). | Yes |
| Limitations | 25 | Discuss limitations at study and outcome level (e.g., risk of bias), and at review-level (e.g., incomplete retrieval of identified research, reporting bias). | Yes |
| Conclusions | 26 | Provide a general interpretation of the results in the context of other evidence, and implications for future research. | Yes |
| **FUNDING** | | |  |
| Funding | 27 | Describe sources of funding for the systematic review and other support (e.g., supply of data); role of funders for the systematic review. | Yes |

*From:*  Moher D, Liberati A, Tetzlaff J, Altman DG, The PRISMA Group (2009). Preferred Reporting Items for Systematic Reviews and Meta-Analyses: The PRISMA Statement. PLoS Med 6(7): e1000097. doi:10.1371/journal.pmed1000097
